# Improved cytosine base editors generated from TadA variants

**DOI:** 10.1038/s41587-022-01611-9

**Published:** 2023-01-09

**Authors:** Dieter K. Lam, Patricia R. Feliciano, Amena Arif, Tanggis Bohnuud, Thomas P. Fernandez, Jason M. Gehrke, Phil Grayson, Kin D. Lee, Manuel A. Ortega, Courtney Sawyer, Noah D. Schwaegerle, Leila Peraro, Lauren Young, Seung-Joo Lee, Giuseppe Ciaramella, Nicole M. Gaudelli

**Affiliations:** Beam Therapeutics, Cambridge, MA USA

**Keywords:** Protein design, Targeted gene repair, Molecular evolution

## Abstract

Cytosine base editors (CBEs) enable programmable genomic C·G-to-T·A transition mutations and typically comprise a modified CRISPR–Cas enzyme, a naturally occurring cytidine deaminase, and an inhibitor of uracil repair. Previous studies have shown that CBEs utilizing naturally occurring cytidine deaminases may cause unguided, genome-wide cytosine deamination. While improved CBEs that decrease stochastic genome-wide off-targets have subsequently been reported, these editors can suffer from suboptimal on-target performance. Here, we report the generation and characterization of CBEs that use engineered variants of TadA (CBE-T) that enable high on-target C·G to T·A across a sequence-diverse set of genomic loci, demonstrate robust activity in primary cells and cause no detectable elevation in genome-wide mutation. Additionally, we report cytosine and adenine base editors (CABEs) catalyzing both A-to-I and C-to-U editing (CABE-Ts). Together with ABEs, CBE-Ts and CABE-Ts enable the programmable installation of all transition mutations using laboratory-evolved TadA variants with improved properties relative to previously reported CBEs.

## Abstract (web summary)

Improved cytosine base editors are generated by directed evolution of an adenosine deaminase.

## Main

Cytosine base editors (CBEs) are gene-editing enzymes capable of programmably introducing C·G-to-T·A base pair changes in the genomic DNA of living cells. This chemical conversion is achieved through enzyme-mediated hydrolytic deamination of cytosine to uracil, which is interpreted as thymine by DNA polymerases^1^. To date, CBEs are typically composed of four distinct components: a naturally occurring cytidine deaminase (such as APOBEC, AID or CDA)^2^, an impaired form of Cas9 capable of nicking the non-base-edited strand of DNA, one or more units of uracil glycosylase inhibitor (UGI) peptide and a nuclear localization sequence (NLS)^1–3^. These components are typically covalently fused but may also be noncovalently assembled^4^. CBEs have been widely exploited for gene reversion and cellular engineering and have the potential to provide therapeutic benefits to patients living with debilitating genetic diseases or malignancies^5^.

Although high on-target DNA editing efficiency can be achieved with current CBE base editing tools^2^, they can also cause genome-wide, stochastic, guide RNA (gRNA)-independent off-target editing^6,7^. Next-generation CBE editors such as YE1 (ref. 8), BE4-PpAPOBEC^9^ and others^10^ have been reported to mitigate gRNA-independent off-target outcomes, but these editors use natural or lightly engineered variants of APOBEC deaminase and may suffer from decreased on-target editing performance^2,9^. Additionally, in some sequence-specific contexts, APOBEC-based CBEs may lead to proximal editing adjacent to the targeted genomic sequence due to APOBEC’s inefficient, but measurable, ability to accept double-stranded DNA (dsDNA) as a substrate^11^.

Adenine base editors (ABEs) are gene-editing enzymes that programmably install A·T to G·C point mutations at targeted loci via a laboratory-evolved TadA deaminase that chemically converts adenine to inosine^12^. Inosine base pairs with cytosine within the active site of DNA polymerases resulting in an inosine to guanine mutation following DNA replication. Notably, ABEs cause low to no gRNA-independent off-targets and edit genomic DNA (gDNA) within a more precise window (positions ~3–8, PAM 21–23), which may result in fewer guide-dependent off-targets as well as fewer bystander edits, relative to CBEs^6,13^. Additionally, ABEs have not been reported to act on dsDNA.

To confer the favorable attributes of ABEs upon a CBE, we envisioned transforming TadA into an enzyme capable of robust cytidine deamination and subsequently generated an improved class of CBEs that uses TadA instead of a naturally occurring cytidine deaminase. Encouragingly, previous investigations have demonstrated ABEs’ malleability toward low, but detectable C to T editing through the inclusion of UGI^14^. Indeed, through structure-guided design, an ABE variant has been reported, ABE-P48R-UGI, that enabled enhanced cytosine activity, relative to ABE7.10, but with high TC sequence specificity^15^. While these advancements represented progress toward our aims, we recognized that further engineering and evolution of TadA would be required to achieve therapeutically relevant C·G-to-T·A editing efficiencies with high product purity and without substrate sequence restrictions.

Starting with ABE8.20-m^13^ as a template for library generation, we conducted two rounds of directed evolution to generate base editor variants with improved C·G to T·A editing efficiencies and retention of adenine editing. We refer to these cytosine and adenine base editors (CABEs) utilizing TadA as ‘CABE-Ts’, and further developed and characterized these editors for C·G-to-T·A and A·T-to-G·C editing efficiencies in mammalian cells. With CABE-Ts in hand, we determined crystal structures of the TadA deaminase variants associated with these editors and performed structure-guided mutagenesis to create CBE-Ts, a distinct class of CBEs that use engineered TadA deaminases for high C·G to T·A conversion in gDNA with no appreciable levels of A·T to G·C editing. Relative to BE4, our CBE-Ts demonstrated comparable on-target editing efficiencies, had a more precise editing window, reduced guide-dependent off-target editing, and showed no detectable gRNA-independent genome-wide off-target editing. Furthermore, CBE-Ts demonstrated compatibility with orthogonal Cas enzymes, allowing for their potential application across a broader range of target sites. Finally, our CBE-Ts were highly active in primary cell types such as T cells and hepatocytes, thus validating their potential as an attractive gene-editing tool for therapeutic applications.

## Results

### Directed evolution of ABE for C-to-T editing

**Fig. 1:**
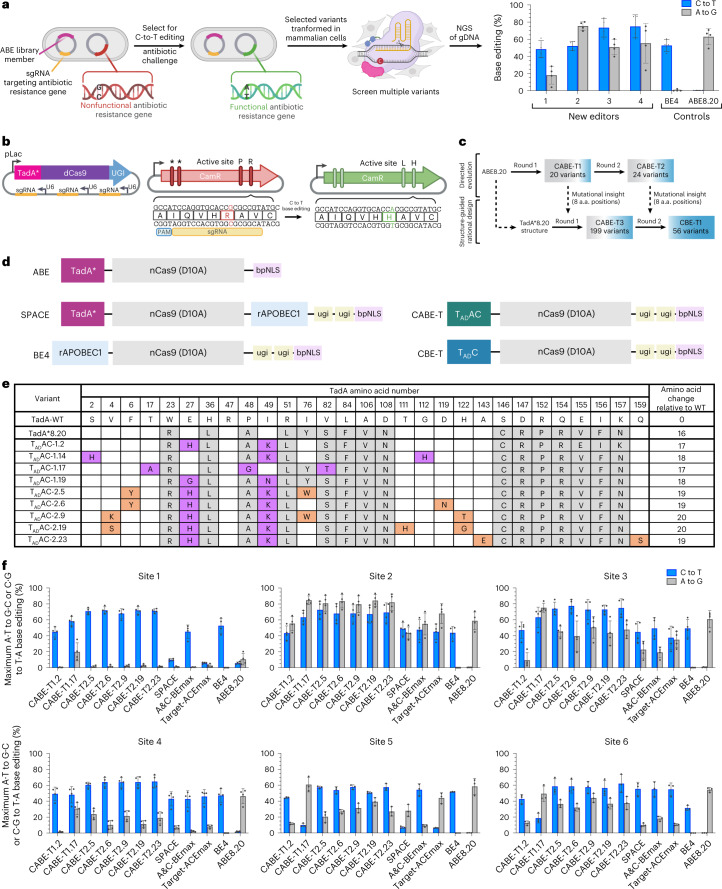
Directed evolution of CABE-T1 and CABE-T2. **a**, Schematic overview of directed evolution workflow to identify TadA variants capable of C-to-U deamination. **b**, Schematic representation of expression and selection plasmids used in directed evolution campaigns. Left: base editor expression plasmid encoding library member and sgRNA, right: selection plasmid encoding a nonfunctional antibiotic resistance gene. Reversion at targeted sites restores gene function. **c**, Overview of CBE-T editor development through directed evolution and protein engineering. **d**, Schematic representation of select base editor architectures. ABE^12^ contains a laboratory-evolved TadA* deaminase, nCas9 (D10A) and a nuclear localization tag (bpNLS). Dual editor (for example, SPACE^16^) is comprised of an evolved TadA* deaminase, nCas9 (D10A), cytidine deaminase rAPOBEC1 along with two units of UGI and a bpNLS tag. CBEs (for example, BE4 (ref. 3)) are comprised of a naturally occurring cytidine deaminase (for example, rAPOBEC1), nCas9 D10A, two units of UGI and a bpNLS tag. CABE-Ts, reported here, are comprised of a TadA variant capable of A-to-G and C-to-T editing (T_AD_AC), nCas9 (D10A), two units of UGI and a bpNLS tag. CBE-Ts, reported here, are comprised of a TadA variant capable of C-to-T editing (T_AD_C), nCas9 (D10A), two units of UGI and a bpNLS tag. **e**, Substitutions incorporated into TadA in selected CABE-T editors from directed evolution round 1 (CABE-T1) and round 2 (CABE-T2). Substitutions incorporated in TadA*8.20 related to wild-type (WT) TadA are highlighted in gray, and those identified in directed evolution round 1 and 2 are highlighted in violet and orange, respectively. Values in the last column represent the number of substitutions added to each variant compared to WT TadA. **f**, Maximum C·G to T·A and A·T to G·C conversion in HEK293T cells transfected with human expression plasmids encoding CABE-T1 and CABE-T2 editors or controls. Values and error bars reflect the mean and s.d. of *n* = 3 (sites 1–4) or 4 (sites 5 and 6) independent biological replicates performed on different days. Source data

To alter the nucleobase substrate tolerance of ABE, we reasoned that we could selectively pressure ABE to increase its low, but detectable^13,14^, C·G-to-T·A base editing capability through directed evolution and inclusion of UGI (to inhibit uracil repair by UNG^2^). First, we generated an ABE library chemically randomized in the TadA region of the editor (ABE8.20-m^13^ used as a template) or randomized via error-prone PCR (ABE8.19-m^13^ used as a template). The resulting ~10-million-member library contained an average of three amino acid substitutions per member. *Escherichia coli* were co-transformed with the ABE library, gRNA and a selection plasmid and were later challenged with lethal doses of antibiotic that were selected for ABE library members that performed C·G to T·A edits within a corresponding antibiotic resistance, restoring gene function (Fig. 1a–d and Supplementary Sequence 1). Sanger sequence analysis of surviving library members (Fig. 1e and Supplementary Fig. 1) revealed that the majority of antibiotic-selected clones contained amino acid substitutions at positions 27 and 49 of TadA. Because 19 of 20 variants contain at least one substitution in either position, we hypothesized that substitutions at these positions (E27H and I49K) located near the substrate binding pocket would induce conformational changes rendering TadA capable of binding and deaminating the cytosine nucleobase, which is notably smaller in size than adenine.

Of the surviving library members, 20 variants were characterized in mammalian cells for base editing outcomes and many variants identified from the first round of evolution demonstrated appreciable levels of C·G to T·A editing (for example, CABE-T1.2, avg 32.1%; CABE-T1.17, avg 34.9% across 22 genomic sites), with varying degrees of A·T to G·C editing retained (Fig. 1f and Supplementary Figs. 2–4). Architecturally, these base editors are comprised of a TadA variant covalently fused to the N-terminal end of a Cas9 nickase (nCas9, D10A) followed by two C-terminal UGI units and a nuclear localization tag (Fig. 1d). Accordingly, we refer to these dual A·T to G·C and C·G to T·A editors as CABE-T1s (Fig. 1c). CABE-T1s elicited an average of >25-fold C·G to T·A editing increase over ABE8.20-m at genomic sites tested (Fig. 1f and Supplementary Figs. 2–4).

To further increase the overall editing efficiency of CABE-T1, we created an ~10-million-member CABE-T library on the background sequence of CABE-T1.2, a CABE-T1 that demonstrated robust C·G to T·A editing in mammalian cells (Fig. 1f). We required CABE-T1.2 library members to create two C·G to T·A reversions, in addition to two A·T to G·C edits for increased stringency in the selection, to survive antibiotic exposure at higher concentrations than in the previous round of directed evolution (Fig. 1b and Supplementary Sequence 2). The surviving library members, referred to as CABE-T2 variants, were sequence identified and evaluated for base editing efficiency and nucleobase substrate bias in mammalian cells (Fig. 1e,f and Supplementary Fig. 5). Overall, mammalian transfection experiments revealed an improvement in our CABE-T2s over CABE-T1s. For example, representative variants CABE-T2.6, CABE-T2.9 and CABE-T2.19 were able to achieve average maximum C·G to T·A editing rates of 53.0%, 53.6% and 49.4% across 22 genomic sites, respectively, while also maintaining various levels A·T to G·C editing (Fig. 1f and Supplementary Figs. 2, 3 and 6).

While CABEs have previously been reported in the literature, these tools have required the inclusion of both TadA*7.10 and rAPOBEC1 deaminases to enable adenine and cytosine base editing from a single full-length editor^16–19^. The creation of CABE-Ts that use one TadA variant that acts on both DNA adenines and cytosines (T_AD_AC) resulted in the generation of a more compact base editor (~700 bp smaller) with superior dual base editing outcomes relative to previously described CABEs. For instance, CABE-T2.6 demonstrated ~1.6-fold higher maximum C·G to T·A and ~2.6-fold higher maximum A·T to G·C relative to SPACE^16^, A&C-BEmax^17^ and TargetACEmax^18^ (Fig. 1f and Supplementary Figs. 2 and 3).

### Structural basis for T_AD_AC substrate tolerance

To illuminate how amino acid substitutions identified from directed evolution affect T_AD_AC’s substrate tolerance, we determined crystal structures of TadA*8.20 from ABE8.20-m^13^, the template used to evolve CABE-T1, and T_AD_AC-1 variants from CABE-T1 reported here. Using structural insights, we aimed to optimize C·G-to-T·A editing efficiency and substrate specificity through structure-guided library design.

**Fig. 2:**
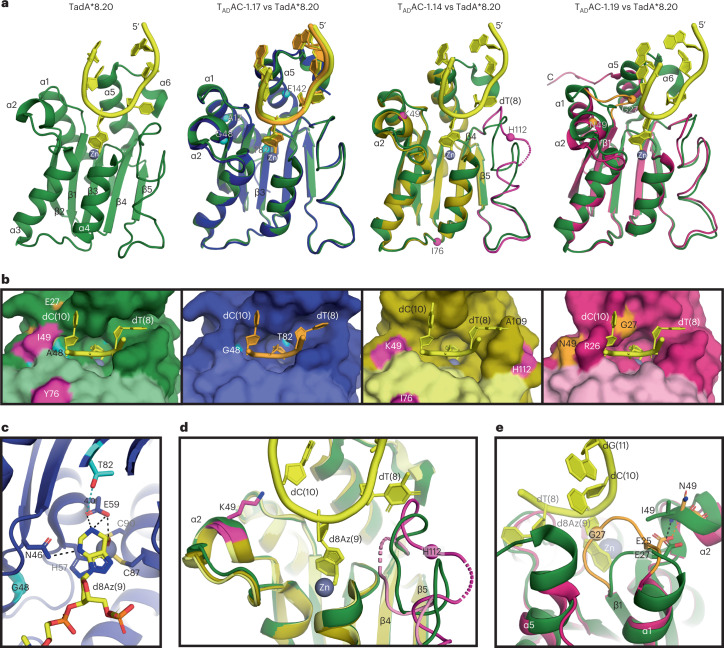
Crystal structures of TadA*8.20 and T_AD_AC-1 variants. **a**, Superpositions between monomers of TadA^*^8.20 and T_AD_AC-1 variants (RMSD between 0.4 Å and 0.9 Å for all of the Cα atoms). T_AD_AC-1.17 (protein—blue, ssDNA—orange) and TadA*8.20 (protein—green, ssDNA—yellow) structures are similar (RMSD of 0.4 Å for all of the Cα atoms), showing that the substitutions (cyan spheres) neither change the protein structure nor the ssDNA binding mode but may impact catalysis due to the active site substitution S82T. T_AD_AC-1.14 structure (yellow) has a different conformation for the loop between β4 and β5 (magenta) than TadA*8.20 due to the substitution G112H (magenta sphere). T_AD_AC-1.19 structure (pink) has an extended loop between α1 and β1 (orange) with a different conformation than TadA*8.20 due to the substitution E27G (orange sphere). C represents the C-terminus. **b**, Surface of the active site cavities of TadA*8.20 and T_AD_AC-1 variants. T_AD_AC-1.17 (second panel) shows no difference compared to TadA*8.20 (first panel). T_AD_AC-1.14 loop between β4 and β5 (third panel) alters the shape of the active site cavity, making A109 steric clash with dT(8) of TadA*8.20-ssDNA. T_AD_AC-1.19 loop between α1 and β1 (fourth panel) alters the shape of the active site cavity, making the residue R26 steric clash with dC(10) of TadA^*^8.20-ssDNA. **c**, T_AD_AC-1.17 active site with adenine transition-state analog 2-deoxy-8-azanebularine (d8Az; yellow) coordinated to the zinc ion (gray sphere). T82 is 4 Å (cyan dashed line) from the catalytic residue E59. Black dashed lines indicate hydrogen bonds. **d**, Superposition between substrate-free T_AD_AC-1.14 monomers (light and dark yellow) and ssDNA-bound TadA*8.20 monomer (protein—green, ssDNA—yellow) showing two different conformations for the T_AD_AC-1.14 loop between β4 and β5 (pink and magenta) compared to TadA^*^8.20. This loop contains the substitution G112H, and its new conformations would make steric clashes with dT(8). K49 is near the ssDNA backbone (~4.5 Å from dC(10) backbone) and may contribute to stabilizing the protein–DNA complex. **e**, Superposition between substrate-free T_AD_AC-1.19 (pink) and ssDNA-bound TadA^*^8.20 monomers showing that E27G substitution places E25 in T_AD_AC-1.19 (orange) at a similar position to E27 in TadA^*^8.20 (green) and induces a new conformation for the T_AD_AC-1.19 loop between α1 and β1 (orange). Dashed lines indicate hydrogen bonds.

Following the first round of directed evolution, we structurally characterized three deaminases corresponding to CABE-T1 (T_AD_AC-1.17, T_AD_AC-1.14 and T_AD_AC-1.19) that generated appreciable levels of C·G to T·A base editing. Although the overall structures of these variants are similar to that of TadA*8.20 (Fig. 2a), structural analyses revealed local structural changes that may explain the observed expanded substrate tolerance exhibited by CABE-T1 variants.

**Extended Data Fig. 1:**
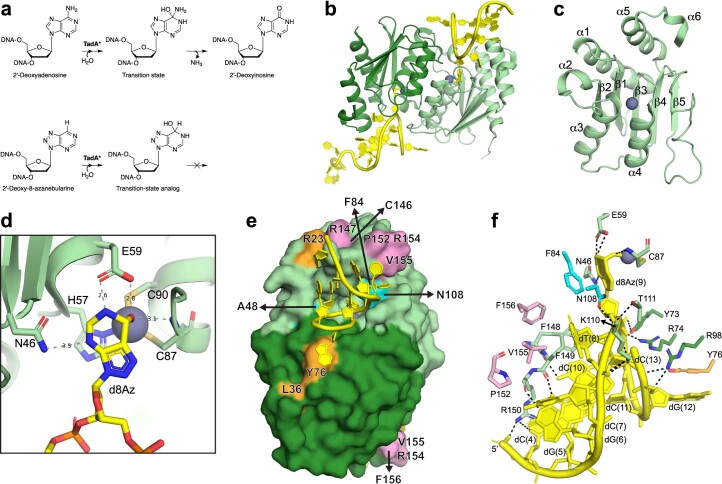
Crystal structure of TadA*8.20 in a complex with ssDNA containing the adenine transition-state analog 2-deoxy-8-azanebularine. **a**, The top panel depicts hydrolytic deamination of adenosine catalyzed by TadA. The bottom panel shows the hydration of the adenine analog, 2’-deoxy-8-azanebularine (d8Az), forming the transition-state analog that gets trapped in the active site by coordinating with zinc. **b**, The overall structure of the TadA*8.20 functional homodimer (chain A in dark green; chain B in light green) bound to ssDNA (yellow). **c**, The overall structure of TadA*8.20 monomer. The monomer (light green) contains five β-strands (β1 to β5) and six α-helices (α1 to α6) that fold into a single domain with a central five-stranded β-sheet surrounded by α-helices. The zinc ion is shown as a gray sphere. **d**, TadA*8.20 active site with ssDNA-d8Az transition-state analog bound. The catalytic zinc (gray) ion is coordinated to a histidine residue (H57), two cysteine residues (C87 and C90), and the d8Az transition-state analog (yellow). The hydrogen bonds are shown as gray dashed lines. **e**, The surface of TadA*8.20 dimer (dark and light green) bound to ssDNA (yellow), showing that ssDNA is bound in the active site deep cavity located at the protein dimer interface and interacts with residues from both monomers, including the substitutions I76Y, L84F, D108N, R152P, E155V, and I156F relative to the wild-type TadA. The substitutions at the protein surface are shown in orange (W23R, H36L, R51L, I76Y, and A106V), at the C-terminal in pink (S146C, D147R, R152P, Q154R, E155V, I156F, and K157N), and at the active site in cyan (P48A, V82S, L84F, and D108N). **f**, Interactions between ssDNA (yellow) and TadA*8.20 active site residues. The residues from chains A and B are shown in dark and light green, respectively. The protein surface, C-terminal, and active site substitutions are shown in orange (chain A), pink (chain B), and cyan (chain B), respectively. The zinc ion is shown in a gray sphere. The hydrogen bonds are shown as black dashed lines. A stereo view is shown in Supplementary Fig. 7. Source data

**Extended Data Fig. 2:**
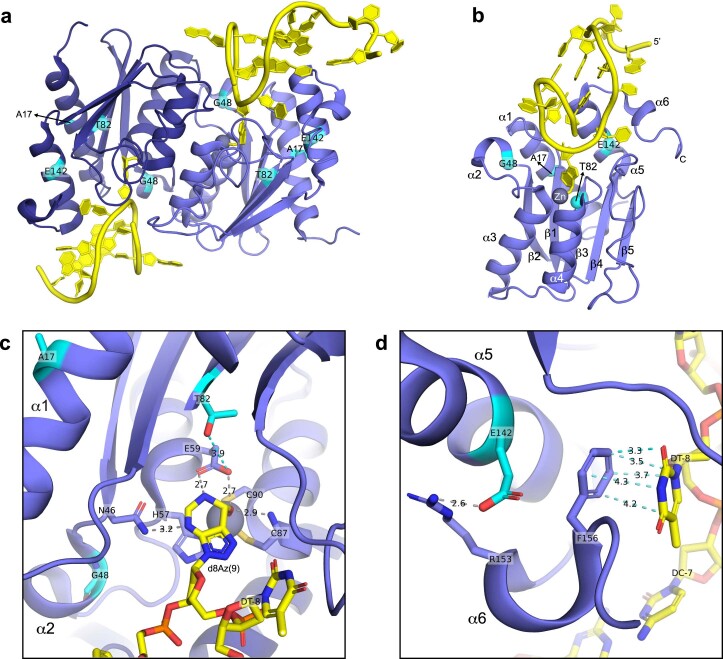
Crystal structure of T_AD_AC-1.17 in a complex with ssDNA containing the adenine transition-state analog 2-deoxy-8-azanebularine. **a**, The overall structure of the T_AD_AC-1.17 functional homodimer (chain A in dark blue; chain B in slate blue) with ssDNA (yellow) bound. The substitutions (T17A, A48G, S82T, and A142E) relative to TadA*8.20 are shown in cyan spheres. **b**, The overall structure of T_AD_AC-1.17 monomer (slate blue) in a complex with ssDNA (yellow). The monomer contains five β-strands (β1 to β5) and six α-helices (α1 to α6) that fold into a single domain with a central five-stranded β-sheet surrounded by α-helices. C and 5′ represent the C-terminus and 5′-end of the ssDNA, respectively. **c**, T_AD_AC-1.17 active site with ssDNA-d8Az transition-state analog bound. The catalytic zinc ion (gray sphere) coordinates H57, C87, C90, and the d8Az transition-state analog (yellow). The T82 side chain (cyan) is near the catalytic E59 side chain (3.9-Å; cyan dashed line) and may play a role in deamination by donating/accepting a proton to/from E59. The residue A17 (cyan) is in α1-helix at the protein surface. The residue G48 (cyan) is in α2-helix at the substrate binding pocket. The H-bonds between the d8Az transition-state analog and protein residues are shown as gray dashed lines. **d**, The side chain of the E142, located in α5-helix, H-bonds (gray dashed line) to the R153 side chain, located in α6-helix, and helps stabilize the C-terminal α6-helix to position the F156 side chain to interact (cyan dashed lines) with the pyrimidine base of dT(8).

Crystal structures of TadA*8.20 and T_AD_AC-1.17 were determined in a complex with ssDNA substrate containing the adenine transition-state analog 2-deoxy-8-azanebularine (d8Az) (Fig. 2, Extended Data Figs. 1 and 2 and Supplementary Figs. 7–10). These two structures are highly similar, and the four T_AD_AC-1.17 substitutions (T17A, A48G, S82T and A142E) derived from evolution do not drastically alter substrate tolerance by changing the protein structure or the ssDNA binding mode (Fig. 2a,b). These findings correlate with T_AD_AC-1.17’s relatively low C·G to T·A reversions at genomic site 5 compared to other variants in CABE-T1 (Supplementary Fig. 4). We hypothesize that the T82 side chain near the catalytic E59 residue (~4 Å) in the active site may have a role in increasing cytosine deamination by modulating proton transfer to or from E59 (Fig. 2c). Additionally, the hydrogen bonding between E142 and R153 may modulate ssDNA binding by stabilizing the α6-helix, as exemplified by the interactions between F156 and dT(8) in the T_AD_AC-1.17 structure (Extended Data Fig. 2d).

**Extended Data Fig. 3:**
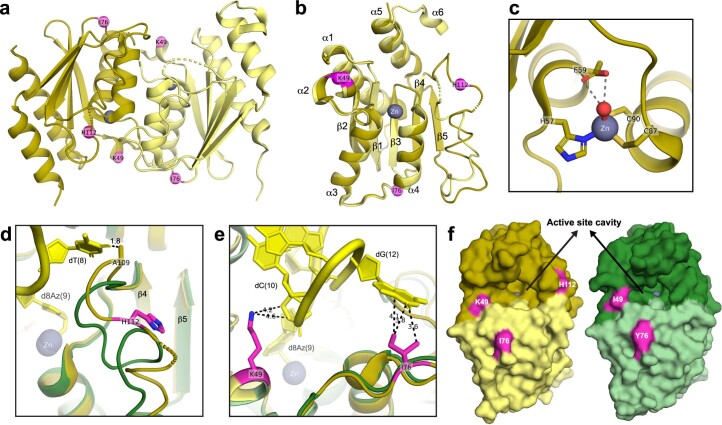
Crystal structure of T_AD_AC-1.14 without ssDNA and structural comparisons with TadA*8.20. **a**, The overall structure of the T_AD_AC-1.14 functional homodimer (chain A in dark yellow; chain B in light yellow). The substitutions (I49K, Y76I, and G112H) relative to TadA*8.20 are shown in magenta spheres. The residue H2 is disordered and not visualized in the structure. The zinc ion is shown as a gray sphere. The dashed line represents the partially disordered (A109 to A114) loop between β4 and β5 (R107 to V130). **b**, The overall structure of T_AD_AC-1.14 monomer. The superposition between chains A (dark yellow) and B (light yellow) shows two different conformations for the loop between β4 and β5 (R107 to V130). **c**, T_AD_AC-1.14 active site with water (red sphere) bound to the zinc ion (gray sphere). The residues H57, C87, and C90 coordinate with the zinc ion. The water molecule (red sphere) H-bonds (gray dashed lines) to the catalytic residue E59. In the first step of the TadA reaction, this water is added to the substrate to form a transition state specie (Extended Data Fig. 1a). **d–f**, Structural comparisons between substrate-free T_AD_AC-1.14 (dark yellow) and ssDNA-bound TadA*8.20 (dark green and yellow) structures. The T_AD_AC-1.14 loop between β4 and β5, which contains the substitution G112H (magenta), has a different conformation than TadA*8.20 and may impact ssDNA (yellow) binding by making steric clashes between the residue A109 and the base dT(8) (1.8-Å**)**, which is adjacent to the target base d8Az(9) (**d**). The substitution Y76I (magenta) may not affect ssDNA binding by conserving interactions (black dashed lines) with the base dG(12) (**e**). The substitution I49K positions the K49 side chain nearby the dC(10) backbone (~4.5-Å; black dashed lines) and may contribute to stabilizing the protein-DNA complex (**e**). The surface of T_AD_AC-1.14 (dark and light yellow) shows that the novel conformation of the loop between β4 and β5 alters the shape of the active site cavity compared to TadA*8.20 (dark and light green) (**f**).

Notably, the crystal structure of T_AD_AC-1.14 containing four substitutions (S2H, I49K, Y76I and G112H) reveals a structural difference in the loop between strands β4 and β5 (R107 to V130) on the right side of the active site cavity (Fig. 2a,d, Extended Data Fig. 3 and Supplementary Fig. 11). This loop contains a G112H substitution that dramatically alters its flexibility and conformation relative to TadA*8.20 by introducing a bulky positively charged residue (Extended Data Fig. 3d). These structural changes may reshape the T_AD_AC-1.14 active site cavity to accommodate both adenines and cytosines (Fig. 2b and Extended Data Fig. 3f). Indeed, a comparison with the structure of TadA*8.20 bound to ssDNA substrate shows that T_AD_AC-1.14 may engage ssDNA differently than TadA variants with strict adenine specificity (Fig. 2b and Extended Data Fig. 3d). We hypothesize that residue K49 within T_AD_AC-1.14 may contribute to the stabilization of protein–DNA interactions required for binding cytosine-containing ssDNA substrates due to its repositioning near nucleobase dC(10) (~4.5 Å) (Fig. 2d and Extended Data Fig. 3e).

**Extended Data Fig. 4:**
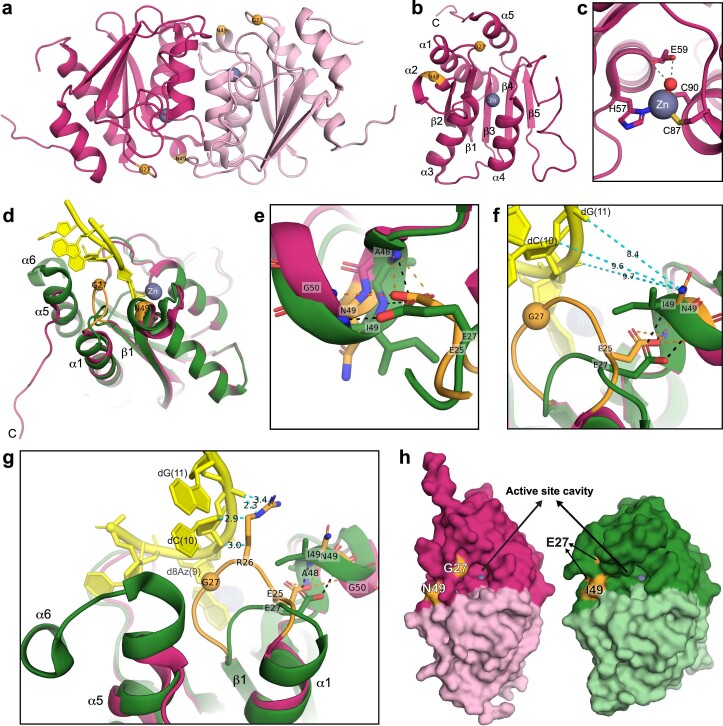
Crystal structure of T_AD_AC-1.19 without ssDNA and structural comparisons with TadA*8.20. **a**, Overall structure of the T_AD_AC-1.19 functional homodimer (dark and light pink). E27G and I49N substitutions relative to TadA*8.20 are shown in orange spheres. Zinc ion is shown as a gray sphere. **b**, Overall structure of T_AD_AC-1.19 monomer. C represents C-terminus. **c**, T_AD_AC-1.19 active site with water (red sphere) bound to the zinc ion. H57, C87, and C90 coordinate with the zinc ion. The water molecule H-bonds (dashed lines) to the catalytic residue E59. **d–h**, Structural comparisons between T_AD_AC-1.19 and TadA*8.20 structures. **(d)** Superposition between substrate-free T_AD_AC-1.19 (pink) and ssDNA-bound TadA*8.20 (green, yellow) monomers, showing high structural similarity (RMSD of ~0.9-Å for all of the Cα atoms). The main structural differences are in α1-helix, the loop between α1 and β1, and C-terminal α5- and α6- helices. **(e)** TadA*8.20 has E27 side chain H-bonding (black dashed lines) to the main chains of A48, I49, and G50, and E27G substitution removes these interactions. To compensate for these critical contacts, T_AD_AC1.19 places E25 at a similar position to the formerly occupied by E27 in TadA*8.20 to make the same H-bonds (orange dashed lines) with A48, I49, and G50. E25 displacement shortens α1-helix, and the loop between α1 and β1 (orange), containing E27G substitution, is extended and adapts into a different conformation compared to TadA*8.20 **(d, f**, and **g**). This results in partial unfolding of α5-helix to prevent steric clash with this loop conformation and complete unfolding of α6-helix **(d** and **g)**. These structural changes alter the shape of the T_AD_AC-T1.19 active site cavity **(h)**, affecting substrate binding in the active site (Fig. 2b). R26 present in T_AD_AC-T1.19-loop (orange) would make close contacts (cyan dashed lines) with dC(10), adjacent to the target base d8Az(9), and dG(11) of TadA*8.20-ssDNA (**g)**. I49N substitution positions the N49 side chain far from the ssDNA backbone (~9-Å from dG(11); cyan dashed lines) **(f)**, suggesting that a residue with a longer positively charged side chain like lysine would create additional contacts with the ssDNA, as observed in the T_AD_AC-T1.14 structure (Extended Data Fig. 3e).

In addition to the perturbations on the right side of the T_AD_AC-1.14 active site cavity, evaluation of the T_AD_AC-1.19 structure reveals that other substitutions (E27G and I49N) from our evolution caused major structural changes on the left side of the active site cavity (Fig. 2a,b, Extended Data Fig. 4 and Supplementary Fig. 12). These structural changes are likely caused by the E27G substitution, which results in the loss of essential hydrogen bonds between E27 and A48, I49 and G50. Because of these hydrogen bond losses, a conformational change occurred that reoriented residue E25 of T_AD_AC-1.19 to a similar position that was formerly occupied by residue E27 in TadA*8.20 (Fig. 2e and Extended Data Fig. 4e). The displacement of E25 shortens the α1-helix, changes the length and conformation of the loop between α1 and β1 (D24 to P29) containing the E27G substitution and unfolds α5- and α6-helices (Fig. 2a,e and Extended Data Fig. 4), reshaping the T_AD_AC-T1.19 active site cavity and potentially impacting target nucleobase binding within the active site (Fig. 2b).

### Structure-guided design of CABE-T3s and CBE-Ts

**Fig. 3:**
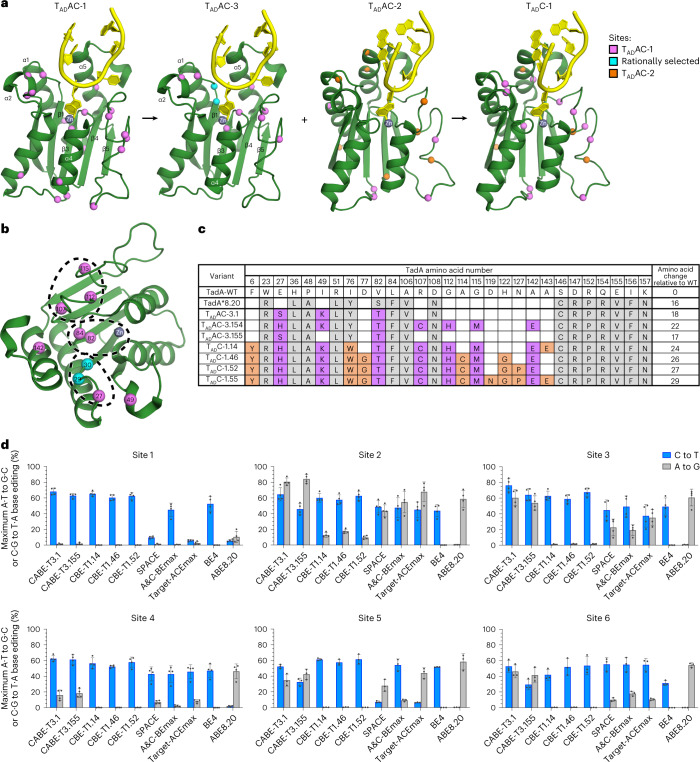
Structure-guided combinatorial screens. **a**, Structure-guided combinatorial library design workflow. For clarity, only one TadA*8.20 monomer (green) with ssDNA (yellow) is shown. The 23 substitution sites identified in the first round of evolution are shown as violet spheres in the first panel. Based on the structures of T_AD_AC-1 variants (Fig. 2), 10 sites were selected, 8 from T_AD_AC-1 (violet) plus 2 rationally selected (cyan), in the loop between α1 and β1, α2-helix, active site, the loop between β4 and β5 and α5-helix, to generate the T_AD_AC-3 variants (second panel). The substitution sites in T_AD_AC-2 from the second round of evolution are shown as violet (same sites identified in T_AD_AC-1) and orange (new sites) spheres in the TadA*8.20 structure (third panel). To generate the T_AD_C-1 variants, eight substitutions from the second round of evolution were added to T_AC_AC-3.154 (fourth panel). **b**, Another view of the TadA*8.20 structure (green) showing the substitution sites selected to generate T_AD_AC-3 (violet and cyan spheres). The dashed circles highlight the three regions that we hypothesized to be critical for altering substrate tolerance. **c**, Substitutions incorporated into selected TadAs from structure-guided screen round 1 (CABE-T3 editors; T_AD_AC-3 deaminases) and round 2 (CBE-T1 editors; T_AD_C-1 deaminases). Substitutions incorporated in TadA*8.20 relative to WT TadA are highlighted in gray, and those identified in directed evolution campaigns 1 and 2 are highlighted in violet and orange, respectively. Values in the last column represent the number of substitutions added to each variant compared to WT TadA. **d**, Maximum C·G to T·A and A·T to G·C conversion at targeted genomic loci in HEK293T cells transfected with human expression plasmids encoding CABE-T3 and CBE-T1 base editors or controls. Values and error bars reflect the mean and s.d. of *n* = 3 (sites 1–4) or 4 (sites 5 and 6) independent biological replicates performed on different days. Source data

Informed by the crystal structures described here (Fig. 2), we speculated that structural changes induced by substitutions in one of three distinct regions of TadA*8.20 (E27G, S82T and G112H) were sufficient to alter substrate tolerance toward a cytosine (Fig. 3b). Thus, we hypothesized that combining amino acid substitutions from all three regions would yield a synergistic improvement in enhancing C·G to T·A editing. To test this hypothesis, eight sites within the deaminase of CABE-T1 were selected for library construction, including substitutions at positions 27, 49, 82, 112 and 142 discussed above, plus two rationally selected sites at or near the active site of TadA*8.20 (Fig. 3a,b) to generate the first combinatorial library containing 199 variants (CABE-T3). Each library member had 2–10 amino acid substitutions (~5.3 on average) in the deaminase of CABE-T3, and most library members encoded at least one amino acid substitution in all three of these regions (Fig. 3b,c and Supplementary Figs. 13 and 14). We identified several CABE-T3 variants, notably CABE-T3.1 and CABE-T3.155, that demonstrate dual C·G-to-T·A and A·T-to-G·C base editing activity at levels comparable to or higher than those from CABE-T1 or CABE-T2 (Fig. 3d and Supplementary Figs. 2, 3 and 15). Notably, by screening library members directly in mammalian cells for relative base editing activity, we were able to identify editors with a broad range of C·G to T·A and A·T to G·C editing ratios, including several variants (for example, CABE-T3.55, T3.153 and T3.154) capable of robust in C·G to T·A editing with minimal A·T to G·C editing (Supplementary Fig. 15).

Concordantly, to further increase overall C·G-to-T·A editing efficiency and optimize substrate specificity toward cytosine, we took CABE-T3.154, a base editor showing a strong preference for C·G to T·A editing (Supplementary Figs. 14 and 15) and combinatorially layered eight additional substitutions selected from the deaminases of CABE-T2s (Fig. 3 and Supplementary Figs. 5 and 6). These substitutions are located proximal to the DNA-binding pocket of the deaminase and their inclusion in CABE-T2s caused an overall increase in editing efficiency compared to CABE-T1s. We generated a 56-member library (Supplementary Fig. 16), screened them in mammalian cells via plasmid transfection, and found that all 56 variants achieved substantial C·G to T·A editing (69.2% averaged across all variants) but caused only low to undetectable levels of A·T to G·C editing (1.8% averaged across all variants at all sites tested; Fig. 3d and Supplementary Figs. 17 and 18). Therefore, we designate these CBEs containing TadAs acting on DNA cytosines (T_AD_C) as CBE-Ts (Fig. 1d).

After observing the robust activity of our CBE-Ts in Hek293Ts, we were curious to evaluate how cytosine base editing outcomes of a representative subset of our 56 CBE-Ts compared to the previously published ABE-P48R-UGI^15^ editor at six genomic sites given the high degree of amino acid substitution per variant that was required to access our editors (Fig. 1e and Supplementary Fig 16). Indeed, we found that our CBE-Ts greatly outperformed ABE-P48R-UGI in C·G to T·A editing efficiency, relative cytosine to adenine base editing product purity and substrate tolerance (Supplementary Fig. 19). Relative to ABE-P48R-UGI, CBE-Ts were not restricted to TC motifs, a limitation of the ABE-P48R-UGI editor, and therefore, we envision the CBE-Ts reported here to be more universally applicable (Supplementary Fig 19).

To determine whether the T_AD_Cs present in our CBE-Ts were compatible with orthogonal Cas enzymes, we screened the base editing activity of a representative subset of our CBE-Ts, replacing the *Streptococcus* *pyogenes* D10A Cas9 nickase with *Staphylococcus aureus* Cas9 nickase (SaCas9, PAM: NGGRRT), shown previously to have compatibility with ABE editors in mammalian cells^13,20^. Indeed, we observed that T_AD_Cs are modular enzymes and are compatible with SaCas9 but elicit only modest C·G-to-T·A editing efficiencies, similar to BE4-SaCas9 variants, across the six genomic sites tested (Supplementary Fig. 20).

### On-target characterization of CABE-Ts and CBE-Ts

**Fig. 4:**
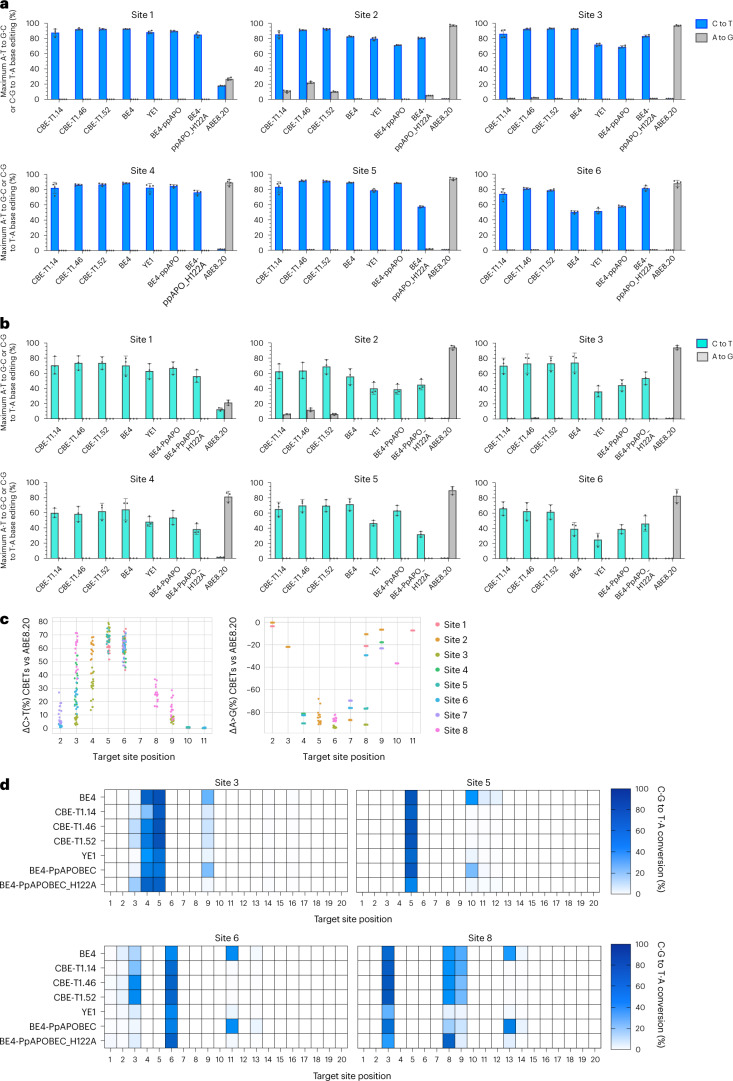
CBE-Ts elicit robust C·G to T·A conversions in human cells at levels comparable to or higher than BE4 with a narrower editing window. **a**,**b**, Maximum C·G to T·A and A·T to G·C conversion in HEK293T cells transfected with mRNA encoding core CBE-T variants, plus controls, across eight targeted genomic loci via synthetic gRNAs at saturating (500 ng mRNA) (**a**) and subsaturating conditions (62.5 ng construct mRNA + 437.5-ng nontranslated carrier mRNA) (**b**). **c**, Percent change in C·G to T·A (left) and A·T to G·C (right) editing rates between CBE-T1 variants and ABE8.20 at each target site position (PAM = positions 21–23) across eight genomic sites tested (sites 1 to 8; Supplementary Table 3). **d**, Median C·G to T·A conversion at each target window position as specified on the *x*-axis, with position numbering defined as the PAM designated as positions 21–23. Values for color maps were determined from mRNA transfections at saturating conditions. Values and error reflect the mean and s.d. of *n* = 4 (saturating conditions) or 3 (subsaturating conditions) independent biological replicates performed on different days. Source data

To more deeply characterize CABE-Ts and CBE-Ts, we chemically synthesized gRNAs and in vitro transcribed (IVT) mRNAs encoding a representative subset of CABE-T and CBE-T editors and transfected them into HEK293T cells at both saturating and subsaturating doses of mRNA encoding the editor (Fig. 4a,b). For the CABE-T2s and T3s tested, we observed an average of 1.53-fold and 1.03-fold increase in maximum C·G to T·A editing and an average of 2.18-fold and 1.67-fold improvement in A·T to G·C editing relative to SPACE and A&C-BEmax, respectively (Supplementary Fig. 21). Across all sites tested, we observed no significant difference in maximum editing outcomes for our characterized CBE-Ts relative to BE4 (*P* = 0.30, two-tailed Wilcoxon–Mann–Whitney *U* test) and remarkable differentiation from editing outcomes relative to the parent editor ABE8.20. Across all sites tested, our CBE-Ts resulted in an average 262-fold increase in C·G to T·A editing and a concordant 13-fold decrease in A·T to G·C editing relative to ABE8.20 across the editing window (Fig. 4a–c and Supplementary Figs. 2 and 3).

**Extended Data Fig. 5:**
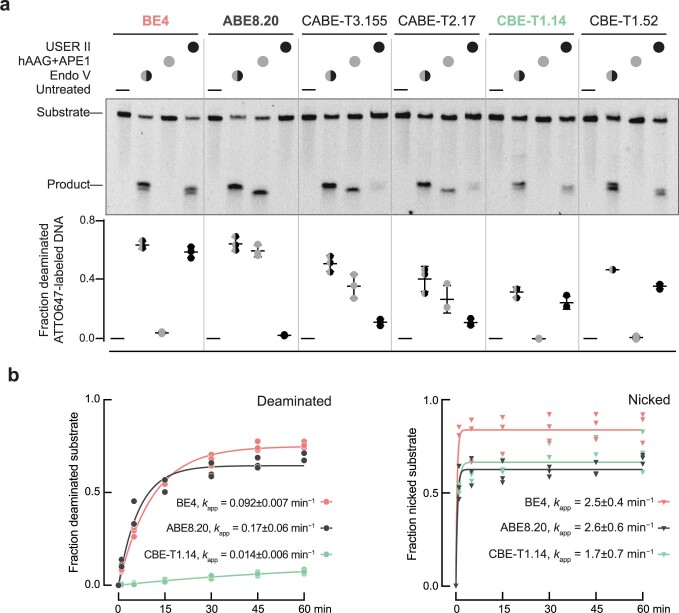
Biochemical characterization of CABE-Ts and CBE-Ts. **a**, *In vitro* 24-hour end-point deamination assay to detect relative A-to-I (hAAG + APE1) and C-to-U (USERII) deamination by BE4, ABE8.20, CABE-Ts, and CBE-Ts programmed with the same guide and acting on the same dsDNA substrate. Endonuclease V, Endo V; human Alkyl Adenine DNA glycosylase, hAAG; Apurinic/apyrimidinic Endonuclease 1, APE1. Error bars represent standard deviation from the mean (plotted) of three independent replicates. Data were normalized to untreated sample. Endo V detects both A-to-I and C-to-U deamination. **b**, Left, Single-turnover rates of A-to-I or C-to-U deamination of the same dsDNA substrate by BE RNP. Right, Single-turnover rates of nicking by BE RNP in the same experiment as shown on left. Pseudo-first order *k*_app_ rate constants obtained by fitting to single exponential are reported (mean ± s.d., n = 3 independent replicates). Source data

To confirm that our CABE-Ts and CBE-Ts proceeded through a C-to-U deamination mechanism, we employed an in vitro end-point deamination assay to evaluate a subset of editors as gRNA-programmed ribonucleoprotein (RNP) complexes acting on dsDNA substrate. In this assay, CABE-Ts and CBE-Ts resulted in an average of ~30% C-to-U substrate deamination after 24 h at the on-target site, compared to ~58% for BE4, with no detectable A-to-I deamination for the CBE-Ts evaluated (Extended Data Fig. 5a). In addition to C-to-U substrate deamination, CABE-Ts also produced up to 35% on-target A-to-I deamination. Altogether, these data provide orthogonal biochemical support for the C-to-U deamination activity of our CABEs and CBEs utilizing TadA variants. In a separate experiment, the C-to-U apparent deamination rate constant (*k*_app_, also referred to as rate) of CBE-T1.14 RNP on dsDNA substrate was measured to be 0.014 ± 0.006 min^−1^, much slower than the rate of A-to-I deamination for ABE8.20 RNP on dsDNA (0.17 ± 0.06 min^−1^; Extended Data Fig. 5b), while their nicking rate for the nontarget strand remained nearly identical (Extended Data Fig. 5b and Supplementary Data Figs. 22 and 23).

Despite having a slower deamination rate in vitro, CBE-T’s and BE4 produced comparable total deamination of target sites in cellular transfections conducted over 5 days (Fig. 4a,b). We hypothesized that, given enough time, total C·G to T·A editing or C-to-U deamination by CBE-Ts would reach levels comparable to the kinetically faster BE4. In agreement with this observation, extending deamination time to 24 h in vitro led to comparable total C-to-U deamination by CBE-Ts and BE4 (Extended Data Fig. 5a).

**Extended Data Fig. 6:**
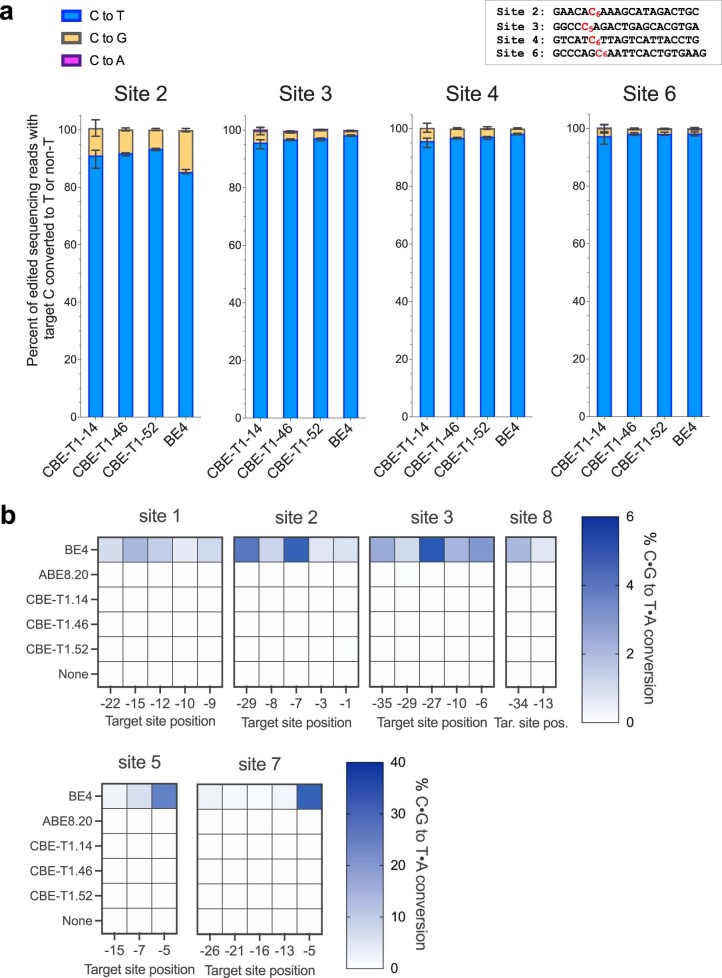
Product purity of CBE-Ts relative to BE4. **a**, Product distribution of sequencing reads mapped as edited for core CBE-Ts and BE4, in which the specified target cytosine (highlighted in red) is mutated. Values were determined from transfection of HEK293T with mRNA at saturating conditions. Values and error bars reflect the mean and SD at n = 3 independent biological replicates performed on different days. **b**, Color map of maximum C·G to T·A conversions outside and 5′ of the protospacer target window. Target site positions where >0.8% C-to-T editing was detected for any editor are included. Values were determined from transfection of HEK293T with mRNA encoding editors or controls at saturating conditions, with n = 4 independent biological replicates performed on different days. Source data

We next evaluated how the dose of delivered mRNA affects cytosine base editing outcomes by conducting mammalian cell transfections at subsaturating levels of mRNA (Supplementary Fig. 24). Under these conditions, CBE-Ts retained 55% to 70% maximum editing efficiency compared to saturating conditions and performed similarly or better relative to APOBEC-based CBEs on a per-site basis. CBE-T representative editors CBE-T1.14, CBE-T1.46 and CBE-T1.52 achieved average maximum C·G to T·A rates of 66% across eight genomic sites, compared to an average 59% C·G to T·A achieved by BE4, and an average of ~35% C·G to T·A achieved by YE1 (Fig. 4b and Supplementary Fig. 25). We also find that CBE-Ts cause similar levels of indel formation and C- to non-T edits (Supplementary Fig. 26 and Extended data Fig. 6a). Comparable product purity and indel outcomes relative to CBEs utilizing cytidine deaminases is likely due to the mechanisms of genomic uracil lesion repair, which is agnostic to how the lesion was created.

Like ABEs, we show that CABE-Ts and CBE-Ts have a narrower editing window relative to BE4, with base edits restricted roughly to positions 3–8 in the protospacer (Fig. 4c and Supplementary Fig. 27). Additionally, we observe that CBE-Ts, relative to APOBEC-based CBEs, generate fewer bystander mutations because on average fewer Cs exist in the narrowed targetable window of the editor (Fig. 4d and Supplementary Fig. 27). For therapeutic applications, we note this increase in base editing precision is an attractive feature when considering disease targets. Relatedly, while BE4 has been characterized to act on dsDNA proximal to the protospacer due to APOBEC’s low, but detectable tolerances for dsDNA as a substrate, we do not observe this dsDNA editing activity with our CBE-Ts (Extended Data Fig. 6b).

### Off-target evaluation of CABE-Ts and CBE-Ts on DNA

**Extended Data Fig. 7:**
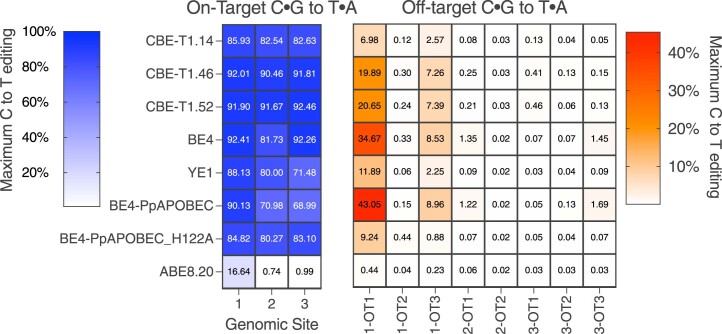
Guide-dependent off-target evaluation of CBE-T. Color map of % maximum on-target C·G to T·A conversion at genomic sites and % maximum C·G to T·A conversion at their corresponding off-target sites in HEK293T cells transfected with mRNA encoding editor (or control) plus synthetic sgRNA at saturating conditions. Median values were derived from n = 3 independent biological replicates performed on different days. Source data

To characterize the gRNA-dependent DNA off-target editing of CBE-Ts and CABE-Ts, we performed mRNA transfections in cells with several gRNAs for which the gRNA off-target profile has been previously characterized with Cas9 and base editors^1,12,13,21^. We find that CBE-Ts and CABE-Ts have lower gRNA-dependent off-target base editing frequencies at all sites examined relative to BE4 and BE4-PpAPOBEC, with 3.06-fold and 3.53-fold decreases in maximum C·G to T·A editing, respectively, and similar levels relative to that of mitigated off-target editors YE1 and BE4-PpAPOBEC-H122A (Extended Data Fig. 7 and Supplementary Figs. 28 and 29).

**Fig. 5:**
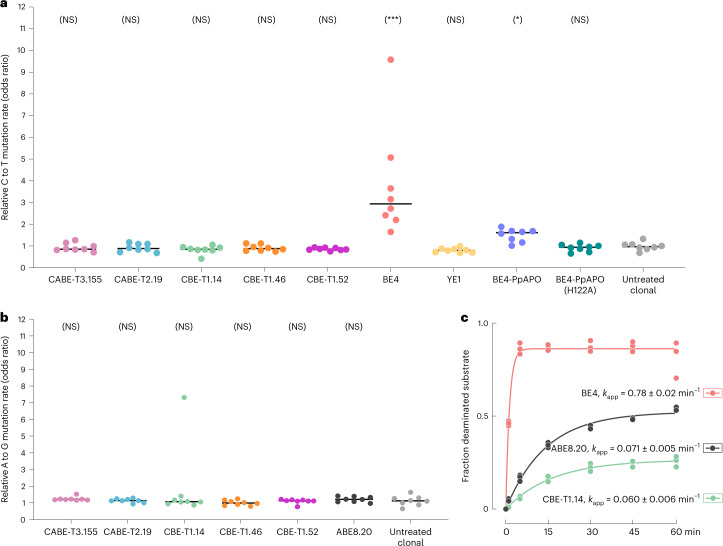
Guide-independent off-target evaluation of CABE-T and CBE-Ts. **a**, Odds ratio plot for C to T mutations relative to all other mutation types in cells edited with CABE-T3.155, CABE-T2.19, CBE-T1.14, CBE-T1.46 and CBE-T1.52, BE4, YE1, BE4-PpAPO and BE4-PpAPO-H122A compared to untreated clonally expanded cells, with black bars representing the median odds-ratio for that treatment group (*P* = 0.8359, 0.7473, 0.9476, 0.8089, 0.9751, 7.770 × 10^−5^, 0.9859, 0.00148 and 0.7473, respectively; one-sided Mann–Whitney *U* test). All *n* = 8 biologically independent single-cell expanded cell populations are shown for each condition. **b**, Odds ratio plot for A to G mutations relative to all other mutation types in cells edited with CABE-T33.155, CABE-T2.19, CBE-T1.14, CBE-T1.46 and CBE-T1.52 and ABE8.20 compared to untreated clonally expanded cells, with black bars representing the median odds-ratio for that treatment group (*P* = 0.1641, 0.4796, 0.5204, 0.8607, 0.5204 and 0.2527, respectively; one-sided Mann–Whitney *U* test). All *n* = 8 biologically independent single-cell expanded cell populations are shown for each condition. **c**, In vitro kinetics of A-to-I or C-to-U deamination of the same substrate presented as ssDNA to BE4, ABE8.20 and CBE-T1.14 in the absence of gRNA. Pseudo first-order apparent rate constants (*k*_app_) obtained by fitting to a single exponential fit are reported (mean ± s.d., *n* = 3 independent replicates). See gel source data. Source data

To evaluate the ratio of guide-independent base editing caused by CABE-Ts and CBE-Ts to BE4 (for C to T editing) or ABE8.20 (for A to G editing), we performed whole genome sequencing (WGS) of clonally expanded cells treated with mRNA encoding a base editor and quantified the relative C·G to T·A or A·T to G·C mutation rate as described before^13^ (Supplementary Fig. 30). We found that both CABE-Ts and CBE-Ts caused no significant elevation in genome-wide C·G to T·A SNVs relative to untreated samples, a pattern that was also reported for YE1 and BE4-ppAPOBEC-H122A (all *P* > 0.05; one-sided Mann–Whitney *U* test). In contrast, BE4 caused a mean fold-enrichment of 3.8 times for C·G to T·A edits over control (*P* = 7.770e−05; one-sided Mann−Whitney *U* test) and BE4-PpAPOBEC caused 1.5 times the mean fold-enrichment of C·G to T·A (*P* = 0.00147; one-sided Mann−Whitney *U* test) (Fig. 5a)^6,13^. CABE-Ts and CBE-Ts also did not cause a significant elevation in genomic A·T to G·C SNVs (all *P* > 0.05; one-sided Mann−Whitney *U* test) (Fig. 5b) and stochastic deamination genome-wide was indistinguishable from untreated cells.

To illuminate the kinetic differences in ssDNA deamination, we measured single turnover, pseudo-first-order apparent deamination rate constants (*k*_app_) of base editors lacking a guide RNA on ssDNA substrate in vitro. We measured the rate of C-to-U deamination by BE4 to be 0.78 ± 0.02 min^−1^, ~11-fold higher than the rate of A-to-I deamination elicited by ABE8.20 (*k*_app_ = 0.071 ± 0.005 min^−1^) for the same ssDNA substrate (Fig. 5c and Supplementary Fig. 22). This difference in deamination rate of ssDNA further supports previous observations, and observations reported here, that APOBEC-based CBEs can stochastically deaminate single-stranded regions of the genome. We found that CBE-T1.14 catalyzed C-to-U deamination with *k*_app_ of 0.060 ± 0.006 min^−1^, a rate indistinguishable from that measured for A-to-I deamination by ABE8.20 (Fig. 5c and Supplementary Fig. 22) on the same ssDNA substrate. Notably, the catalytic residue remains unchanged between ABE8.20 and CBE-Ts (Figs. 2c, 3a and Extended Data Figs. 1d, 2, 3c).

### Application of CBE-Ts in primary cells

**Fig. 6:**
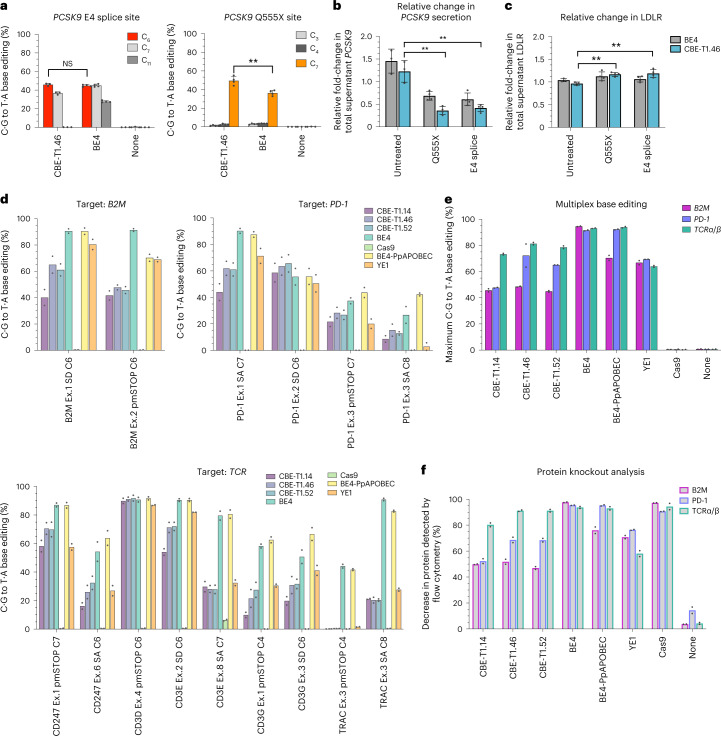
Evaluation of CABE-Ts and CBE-Ts in therapeutically relevant cell contexts. **a**, Gene-editing outcomes of primary human hepatocytes transfected with mRNAs encoding CBE-T1.46 and BE4 at three sites within the *PCSK9* gene (left, *P* = 0.2882 (NS); right, *P* = 0.0017 (double asterisk)). Positional edit within the protospacer indicated. **b**, Evaluation of relative change in *PCSK9* secretion between day 9 (collection time point) and day 0 (transfection time point) through ELISA. The targeted *PCSK9* site is indicated on the *x* axis. *P* values are as follows: Q555X versus untreated, *P* = 0.001001 (double asterisk); E4 splice versus untreated, *P* = 0.001295 (double asterisk). **c**, Relative change in LDL-R present in supernatant between day 9 and day 0 assessed by ELISA. *P* values are as follows: Q555X versus untreated, *P* = 0.001116 (double asterisk), E4 splice versus untreated, *P* = .009481 (double asterisk). **d**, Gene-editing efficiencies from sgRNA screens in primary human T cells using CBE-Ts. *X* axis label indicates the targeted gene and target base within sgRNA. **e**,**f**, Percent C·G to T·A conversion (**e**) and surface protein loss (**f**) achieved by each base editor or control in multiplex-edited primary human T cells. Primary hepatocyte data were generated from *n* = 3 (‘none’/untreated samples) or 4 (BE4 and CBE-T1.46 samples) independent biological replicates. T cell data were generated from *n* = 2 independent donors. Where applicable, statistical significance was computed via two-tailed unpaired *t* tests: NS, *P* ≥ 0.05; *P < 0.05; **P < 0.01. Source data

CBE-Ts have substantial potential for therapeutic use in gene reversion and silencing due to their improved properties relative to CBEs utilizing naturally occurring cytidine deaminases. To evaluate the editing potential of CBE-Ts in primary cells, we first assessed the ability of CBE-Ts to silence the expression of *PCSK9*, a target relevant to the therapeutic treatment of hypercholesterolemia^22^, in a long-lived primary human hepatocyte coculture system^23^. Knock-down or knock-out of *PCSK9* gene results in lower levels of low-density lipoprotein (LDL) cholesterol in the blood and subsequently lowers the risk of heart disease^24,25^. Indeed, promising results with splice-site targeting of *PCSK9* have been achieved with ABE8.8 in vivo^26^. Similarly, we found that mRNA transfection of CBE-T1.46 with synthetic guide targeting the *PCSK9* gene in primary human hepatocytes achieved C·G to T·A base editing efficiencies that are comparable to or greater than BE4 at two *PCSK9* target sites that introduce the stop codon Q555X or disrupt pre-mRNA splicing at exon 4 (E4 splice; Fig. 6a). Evaluation of *PCSK9* protein levels via ELISA shows knockdown of *PCSK9* by CBE-T1.46 at levels comparable to or greater than BE4 at both target sites tested (Fig. 6b). Concordantly, increases in total LDL receptor (LDLR) were observed for the CBE-T1.46 treated samples at both sites (*P* < 0.05, <0.01), demonstrating the potential for CBE-T to generate a therapeutically relevant phenotypic effect (Fig. 6c).

We next evaluated the application of CBE-Ts to therapeutic T cell engineering. Autologous T cell therapies derived from TCRαβ-expressing T cells are effective in treating some cancers, although the manufacture of these cell therapies on a per-patient basis can result in inconsistent products, high cost of goods and significant delays in patient treatment. Gene editing can be used to create universally compatible T cell therapies, generated from single donors for the treatment of many patients^27^. Universally compatible T cell therapies require multigene silencing to eliminate expression of the T cell receptor to reduce the potential for graft-versus-host-disease (GvHD), and editing strategies to reduce or eliminate host rejection of the allogeneic T cells^28^. To determine whether CBE-Ts could be used for T cell editing, we electroporated T cells with mRNA encoding CBE-Ts and gRNAs targeting genes coding for components of the T cell receptor, *B2M* or *CIITA* and found that CBE-Ts yielded comparable or only slightly lower editing efficiencies compared to BE4 controls (Fig. 6d). Multiplexed CBE-T editing demonstrated comparable editing efficiencies compared to single-plex editing, which resulted in corresponding levels of protein knock-down (Fig. 6e,f and Supplementary Fig. 31), demonstrating the potential of the CBE-T platform for therapeutic cellular engineering.

## Discussion

Here we describe the development of two families of base editors, CABE-Ts and CBE-Ts, which use variants of TadA to catalyze the deamination of cytosines with either retention (CABE-Ts) or loss (CBE-Ts) of adenine deamination.

Over the course of ten total rounds of directed evolution and additional rounds of structure-guided design, TadA has matured to include over 29 substitutions in our most engineered CBE-Ts (Fig. 3c). Through X-ray crystallography, we show how the accumulation of substitutions impacts the shape of the active site cavity and may contribute to the accommodation of cytosine as substrate and the subsequent shift in specificity toward C-to-U deamination. The structures developed herein illuminate how amino acid substitutions in TadA influence gene-editing outcomes observed in cells.

The CABE-Ts and CBE-Ts reported here are precision base editors with highly mitigated guide-independent DNA off-target outcomes, fewer bystander edits and fewer guide-dependent DNA off-targets relative to previously reported CBEs due to the difference in kinetics of deamination of ssDNA by the TadA variant used in our CBE-T constructs. TadA-based CBE-Ts and CABE-Ts retain high on-target editing activity, enabling high gene editing efficiencies both in single- and multiplexed applications.

Finally, we show our CBE-Ts are active in therapeutically relevant cell types, including primary hepatocytes and primary T-cells, with editing outcomes similar or superior to what can be achieved with BE4. We demonstrate the ability of CBE-Ts to edit target sites in the *PCSK9* locus to reduce levels of secreted PCSK9 protein, as well as achieve high levels of multiplexed editing at T cell targets relevant for the generation of allogeneic CAR-T cells.

In summary, the development of TadA for use in highly efficient cytosine base editing represents an impactful advancement in the development of CBEs as therapeutic tools. Together with ABEs, CABE-Ts and CBE-Ts enable the programmable installation of all DNA transition mutations within living cells, separately or concurrently, through the use of laboratory-evolved and highly engineered TadA deaminases and consequently extend the potential therapeutic applications of cytosine base editing.

## Methods

### General methods

All molecular biology methods and cloning steps were performed as previously described^13^, including the utilization of USER enzyme (New England Biolabs, NEB, M5505L), Phusion U DNA Polymerase Green Multiplex PCR Master Mix (Thermo Fisher Scientific, F564L), Q5 Hot Start High-Fidelity 2X Master Mix (NEB, M0494L), Mach T1 competent cells (Thermo Fisher Scientific, C8681201) and ZymoPURE II Plasmid Midiprep kits (Zymo Research Corporation, D4201) in accordance with manufacturers’ protocols. Amino acid sequences for base editors highlighted in this study can be found in Supplementary Sequences 3–31. Sequences of sgRNAs used to target genomic sites can be found in Supplementary Table 3. Representative CABE-Ts and CBE-Ts used in this study have been deposited on Addgene.

### Generation of TadA* and T_AD_AC libraries for directed evolution

Synthetic libraries for directed evolution rounds one and two were obtained from Ranomics with the following specifications: evolution round one TadA*8.20 library—each amino acid position of the TadA*8.20 (from ABE8.20) sequence to be represented by all 20 amino acid substitutions at a frequency of 1–3 substitutions per library member (~10 million members). This library excluded all stop sequences and used only one codon per amino acid. This synthetic library was combined with a randomized library generated with error-prone PCR using TadA*8.19 (ref. 13) as a template as previously reported in ref. 12. Evolution round two synthetic library—each amino acid position of the T_AD_AC1.02 sequence to be represented by all 20 amino acid substitutions via at a frequency of 2–3 substitutions per library member (~10 million members). These libraries were cloned into a bacterial expression plasmid containing dead Cas9 (dCas9 D10A and H840A) along with gRNAs targeting the chloramphenicol resistance gene through USER cloning.

### Bacterial evolution of TadA variants

Directed evolution of TadA8.19 and TadA8.20 library (directed evolution round one) and T_AD_AC1.02 library (directed evolution round 2) was conducted as previously described in ref. 13 with the following changes: libraries of various TadA* deaminase variants that are included in a bacterial plasmid containing TadA*-dCas9-UGI editor architecture were challenged to revert edits in the chloramphenicol resistance gene to survive treatment with lethal doses of antibiotic drug. In the first round of directed evolution, the evolution library was a combination of an error-prone ABE8.19m TadA* library and a synthetic ABE8.20m TadA* library where each amino acid position is represented by all 20 substitutions at a frequency of 1–3 substitutions per library member. To overcome the antibiotic challenge, 2 C-to-T reversions (proline reversion and active site His reversion) were needed. In the second round of evolution, a synthetic library of CABE-T1.2 was used, which was generated with the specifications as the ABE8.20 TadA* library but with 2–3 substitutions per library member. To overcome the antibiotic challenge, the same 2 C-to-T reversions plus 2 A-to-G STOP codon reversions were needed.

### General HEK293T mammalian cell culture conditions

HEK293T cells (ATCC, CRL-3216) were cultured in DMEM + GlutaMAX (Gibco, 10569) supplemented with 10% (vol/vol) fetal bovine serum (Gibco, 10437) at 37 °C and 5% CO_2_ in accordance with standard protocols from ATCC and as previously described.^13^

### General HEK293T transfection conditions

For all transfections, HEK293T cells were seeded at a density of 3.0 × 10^5^ cells per well in BioCoat poly-d-lysine coated 48-well plates (Corning, 356509) 16–22 h before transfection. Plasmid transfections were performed using Lipofectamine 2000 (Invitrogen, 11668-019) as previously described.^13^ Transfections with mRNA were performed using Lipofectamine MessengerMAX in accordance with manufacturer protocols, with the following specifics: 500 ng (for saturating conditions) or 62.5 ng (subsaturating conditions) of mRNA encoding for editor or control and 100 ng of synthetic gRNA were combined in 12.5 μl total volume of OptiMEM serum reduced medium (Gibco, 31985). A 12.5 μl 1:12.5 (Lipo:OptiMEM) MessengerMAX mixture was then added to the mRNA/gRNA solution, and the entire contents were left to rest at ambient temperature for 15 min. For mRNA transfections at subsaturating conditions, 437.5 ng of carrier mRNA was also added to maintain equivalent amounts of transfected material. The entire 25 μl mixture was then used to treat the preseeded HEK293T cells. The sequences of sgRNAs used in this study are specified in Supplementary Table 3. Synthetic gRNAs for mRNA transfections have 5′/3′ end-modifications as previously described.^13^

### Targeted amplicon next-generation sequencing of DNA samples

After 4 d of incubation, gDNA from HEK293T cells was harvested from the cells using 100 μl of Quick Extract DNA Extraction Buffer (Lucigen, QE09050) in accordance with manufacturer protocols. For allogeneic T cells, 50 μl of Quick Extract DNA Extraction Buffer was used on 1 × 10^5^ cells at 5–6 d post-transfections. Genomic DNA samples from mammalian cell samples were amplified with primers for site-specific genomic DNA amplification containing adapter sequences compatible with Illumina’s TruSeq HT system (Adapter Read 1 sequence, AGATCGGAAGAGCACACGTCTGAACTCCAGTCA; Adapter Read 2, sequence AGATCGGAAGAGCGTCGTGTAGGGAAAGAGTGT). The sequences of these primers are listed in Supplementary Table 4. Specifically, 2 μl of gDNA was added to a PCR reaction mixture containing Phusion U Green Multiplex Master Mix (Thermo Fisher Scientific, F564L) and 0.5 μM of each forward and reverse primer. These amplicons were then barcoded using Q5 Hot Start High-Fidelity 2X Master Mix, where 2 μl of amplicon from the first round of PCR was added to the master mix containing 0.5 μM of each unique combination of forward and reverse barcode primer. Thermocycling conditions are as follows: 95 °C × 2 min of initial denaturation; 95 °C × 15 s of cycle denaturation; 62 °C × 20 s of annealing; 72 °C × 20 s of extension, with cycle repeats of 30 for the initial amplicon generation and 10 for barcoding. Barcoded amplicons were purified, size selected via gel electrophoresis and gel extracted using the Qiaquick Gel Extraction Kit (Qiagen, 28706×4), and the resultant DNA concentrations were evaluated with a NanoDrop 1000 Spectrophotometer (Thermo Fisher Scientific).

### Data analysis of targeted amplicon next-generation sequencing

All targeted amplicon NGS data were analyzed using methods previously described, including the use of the following tools/software: trimmomatic (v0.39), bowtie2 (v2.35), samtools (v1.9) and bam-readcounts (v0.8).^13^

### Data analysis of WGS data for guide-independent deamination

FASTQ files were aligned to the human genome (Gencode GRCh38v31 primary assembly) using BWA mem2 (bwa-mem2-2.2.1). Alignments were sorted by coordinates, merged if necessary, and duplicates were marked using Picard (v2.21.7) on default settings. Base-quality score recalibration was then performed using GATK (v4.1.4.1) to create a BAM file for input into LoFreq (v2.1.5) for variant calling. Bulk sample 1 was used as the normal sample and each clonally expanded cell was run as a separate tumor sample to identify somatic mutations specific to each cell. LoFreq was run with the ‘–min-cov 10’ flag to require a minimum of ten times coverage at the variant site and somatic variants were analyzed from the somatic_final_minus-dbsnp.snvs output file, to remove common variants that were likely false positives.

For the odds ratio plots, a single representative cell from the untreated clonally expanded cells is required as a reference point to compare with both the treated and untreated cells for both C-to-T and A-to-G deaminations. This cell was selected by ordering the untreated cells by proportion of A-to-G mutations and proportion of C-to-T mutations and selecting the one cell closest to the median for both metrics. N1 was in position 5/8 for C-to-T mutations and position 3/8 for A-to-G mutations, making it the best candidate for the reference cell across both CABE-T and CBE-T treatments.

### Protein expression and purification

TadA*8.20 protein was cloned into a pET51b^+^ vector with His and SUMO tags at the N-terminus and expressed in *E. coli* BL21 Star (DE3) cells (NEB, C2527I) in LB media. Cell cultures were grown at 37 °C with shaking at 240 rpm, and protein expression was induced by 0.5 mM IPTG when OD_600_ reached 0.6. Cell culture was incubated with shaking at 18 °C overnight. Harvested cells were lysed by a high-pressure homogenizer in lysis buffer (25 mM Bis-Tris, 500 mM NaCl, 1 mM TCEP, 10% (vol/vol) glycerol, pH 6.0 and 1 mM PMSF), and the cell lysate was clarified by ultracentrifugation. Clarified lysates were loaded onto Ni-NTA agarose resin by batch binding for 1 h at 4 °C. The resin was washed with lysis buffer with 20 mM imidazole on a gravity flow column followed by elution with the lysis buffer supplemented with 50/100/250 mM imidazole. The eluted sample was incubated with Ulp1 while dialyzed in 25 mM Bis-Tris, 300 mM NaCl, 1 mM TCEP, 10% (vol/vol) glycerol and pH 6.0 overnight. The dialyzed sample was loaded onto Ni-NTA resin to remove uncleaved protein and Ulp1. The flowthrough from reverse Ni-NTA was loaded on a 5 ml Heparin HP column (Cytiva) and eluted using a 0–2 M NaCl gradient. Fractions containing TadA*8.20 protein were further purified by size exclusion chromatography on Superdex75 10/300 in 25 mM Bis-Tris, 300 mM NaCl, 1 mM TCEP, 10% (vol/vol) glycerol, pH 7.0. T_AD_AC-1.14 protein was expressed with N-terminal His-tag in pET51b^+^ vector and purified as described above, except that Ulp1 tag cleavage and reverse Ni-NTA steps were omitted. T_AD_AC-1.17 and T_AD_AC-1.19 were cloned in pD881 vector (ATUM) with N-terminal His-tag and SUMO tag and expressed in *E. coli* BL21 cells (NEB). Protein expression was induced by 0.2% (wt/vol) rhamnose at OD_600_ of 0.6, followed by incubation at 37 °C for 4 h. Purification was performed as described above. These deaminase variants were used for X-ray crystallography studies. All CBE-T base editor proteins used for biochemical studies were expressed and purified as described above with slight modifications.

### Crystallization of TadA*8.20 with ssDNA

The crystallization condition of TadA*8.20 with ssDNA containing the adenine analog 2-deoxy-8-azanebularine (d8Az), 5′-G(1)C(2)T(3)C(4)G(5)G(6)C(7)T(8)d8Az(9)C(10)G(11) G(12)A(13)-3′, was identified and optimized using a Mosquito robot (SPT LabTech) at 20 °C. Drops were prepared by mixing 1 μl of protein plus ssDNA solution (0.15 mM TadA*8.20 in 25 mM Bis-Tris, 300 mM NaCl, 1 mM TCEP, 10% (vol/vol) glycerol, pH 7 and 0.22 mM ssDNA with d8Az) and 1 μl of reservoir solution (27–29% (vol/vol) PEG 3,350, 0.22–0.26 M ammonium acetate, 0.1 M Tris pH 8.5), and equilibrated against 70 μl of reservoir solution. The crystals were transferred to a cryoprotectant solution (15% (vol/vol) glycerol, 29% (vol/vol) PEG 3,350, 0.26 M ammonium acetate, 0.1 M Tris pH 8.5) and flash-cooled in liquid nitrogen.

### Crystallization of T_AD_AC-1.17 with ssDNA

The crystallization condition of T_AD_AC-1.17 with ssDNA containing the adenine analog 2-deoxy-8-azanebularine (d8Az), 5′-G(1)C(2)T(3)C(4)G(5)G(6)C(7)T(8)d8Az(9)C(10)G(11) G(12)A(13)-3′, was identified and optimized using a Mosquito robot (SPT LabTech) at 20 °C. Drops were prepared by mixing 1 μl of protein plus ssDNA solution (0.15 mM T_AD_AC-1.17 in 25 mM Bis-Tris, 300 mM NaCl, 1 mM TCEP, 10% (vol/vol) glycerol, pH 7 and 0.22 mM ssDNA with d8Az) and 1 μl of reservoir solution (4–8% (vol/vol) PEG 3,350, 8–10% Tacsimate pH 6) and equilibrated against 200 μl of reservoir solution. The crystals were transferred to a cryoprotectant solution (12% (vol/vol) PEG 3,350, 10% (vol/vol) Tacsimate pH 6, 25% (vol/vol) glycerol) and flash-cooled in liquid nitrogen.

### Crystallization of T_AD_AC-1.14 without ssDNA

The crystallization condition of T_AD_AC-1.14 without ssDNA (T_AD_AC-1.14-holo) was identified and optimized using a Mosquito robot (SPT LabTech) at 20 °C. Drops were prepared by mixing 1 μl of protein solution (0.18 mM T_AD_AC-1.14 in 25 mM Bis-Tris, 450 mM NaCl, 1 mM TCEP, 10% (vol/vol) glycerol, pH 7) and 1 μl of reservoir solution (1.8–2.0 M ammonium sulfate, 0.1 M HEPES pH 7.5) and equilibrated against 200 μl of reservoir solution. The crystals were transferred to a cryoprotectant solution (1.8 M ammonium sulfate, 0.1 M HEPES pH 7.5, 20% (vol/vol) glycerol) and flash-cooled in liquid nitrogen.

### Crystallization of T_AD_AC-1.19 without ssDNA

The crystallization condition of T_AD_AC-1.19 without ssDNA (T_AD_AC-1.19-holo) was identified and optimized using a Mosquito robot (SPT LabTech) at 20 °C. Drops were prepared by mixing 1 μl of protein solution (0.3 mM T_AD_AC-1.19 in 25 mM Bis-Tris, 300 mM NaCl, 1 mM TCEP, 10% (vol/vol) glycerol, pH 7) and 1 μl of reservoir solution (6–12% (vol/vol) PEG 3,350, 0.3–0.5 M ammonium citrate tribasic pH 7.0) and equilibrated against 200 μl of reservoir solution. The crystals were transferred to a cryoprotectant solution (16% (vol/vol) PEG 3,350, 0.6 M ammonium citrate tribasic pH 7.0, 20% (vol/vol) glycerol) and flash-cooled in liquid nitrogen.

### Data collection and structure determination of TadA*8.20 and T_AD_AC-1 variants

Data collections were performed at the Frontier Microfocusing Macromolecular Crystallography (FMX) beamline of the National Synchrotron Light Source II or the ID30B beamline of the European Synchrotron Radiation Facility, or the BL13-XALOC beamline of the ALBA Synchrotron or the P13 beamline of the EMBL Hamburg at the PETRA III storage ring (DESY). Diffraction data were processed using XDS^29^ and scaled using AIMLESS^30^. The crystal structures of TadA*8.20, T_AD_AC-1.17, T_AD_AC-1.14 and T_AD_AC-1.19 without or with ssDNA were determined by molecular replacement techniques implemented in Phaser^31^. For the TadA*8.20 structure, the coordinates of the *E. coli* TadA structure (Protein Data Bank (PDB) code: 1Z3A)^32^ were used to obtain the initial phases. For T_AD_AC-1.17, T_AD_AC-1.14 and T_AD_AC-1.19 structures, the coordinates of the TadA*8.20 (this study) were used to obtain the initial phases. Following molecular replacement, simulated annealing was performed in phenix.refine^33^ to remove model bias. The models were refined by iterative rounds of model building and the addition of water molecules using Coot^34^. Refinement of the structures in phenix.refine used noncrystallographic symmetry restraints, positional and B-factor refinement, and TLS (translation, libration and screw) (except for T_AD_AC-1.17 and T_AD_AC-1.14). The crystals of TadA*8.20 and T_AD_AC-1.17 are merohedrally twinned with twin fractions of 0.375 and 0.246 by Britton analyses (phenix.xtriage), respectively, and the twin law -h,-k,l was used in refinement. The data collection and refinement statistics are summarized in Supplementary Table 2. The residues and nucleotides visualized in the structures, of 167 residues and 13 nucleotides, are listed in Supplementary Table 5. Figures were created with PyMol Software (Schrodinger, 2010. The PyMOL Molecular Graphics System, Version 2.4.1.).

### Biochemical characterization of deamination by ABEs, CABEs and CBEs

An sgRNA (mG*mA*mA*CACAAAGCAUAGACUGCGUUUUAGAGCUAGAAAUAGC

AAGUUAAAAUAAGGCUAGUCCGUUAUCAACUUGAAAAAGUGGCACCGAGUCGGUGCU*mU*mU*mU; modifications: m, 2′-*O*-methyl, and *, phosphorothioate linkage) was synthesized at Agilent Technologies and Integrated DNA Technologies (IDT). Substrate DNA was synthesized at IDT: DNA strand undergoing deamination (TTCGGTGGCTCCGTCCGTGAACACAAAGCATAGACTGCCGGCGTTTTGGTTGCTCTTCG) was labeled with 5′ ATTO-647 fluorophore and a complementary DNA strand undergoing nicking by D10A-Nickase (CGAAGAGCAACCAAAACGCCGGCAGTCTATGCTTTGTGTTCACGGACGGAGCCACCGAA) was labeled with 5′ 6-FAM fluorophore. For guide RNA-independent deamination, the ATTO-647 labeled single-strand DNA was used as is. For guide RNA-dependent deamination, dsDNA substrate was prepared by annealing the two strands, with twofold excess of the strand undergoing nicking (1:2 nmol). The duplexed DNA was purified by 7.5% Native-PAGE (29:1, acrylamide:bisacrylamide; Sigma). The acrylamide band containing the dsDNA was excised, crushed and rotated overnight in crush-and-soak buffer (400 mM NaCl and 25 mM EDTA) to elute the dsDNA. The eluted dsDNA was precipitated at –20 °C for 2 h after adding 1 volume of 100% 2-propanol, followed by centrifugation at 20,000*g* for 30 min at 4 °C. The DNA pellet was washed with 1 volume of 70% vol/vol ethanol and centrifuged at 20,000*g* for 30 min at 4 °C. The pellet was air-dried at room temperature for 30 min and resuspended in water.

RNP complexes were formed by mixing the sgRNA and the appropriate base editor protein in a 1.5:1 molar ratio in ‘RNP assembly and reaction buffer’ (20 mM HEPES-KOH pH 7.4, 100 mM KCl, 5 mM MgCl_2_, 5% vol/vol glycerol, 2 mM TCEP) and incubating at room temperature for 20 min.

For single turnover kinetics of guide RNA-dependent dsDNA deamination in vitro, to 1-µM final concentration of RNP, a final concentration of 10 nM dsDNA substrate (prepared at 100 nM in RNP assembly and reaction buffer) was added to initiate deamination. The reaction was incubated at 37 °C and aliquots of 5 µl were withdrawn at the indicated time intervals. The reactions were quenched in 50-µl quenching buffer (50 mM Tris–Cl, pH 8.5, 400 mM NaCl, 25 mM EDTA, 0.1% SDS, 1 µl thermolabile proteinase K (New England Biolabs, NEB P8111S) and 1 µl 15 mg ml^−1^ coprecipitant Glycoblue (Thermo Fisher Scientific, A9515)) for 15 min at 37 °C. The thermolabile proteinase K was inactivated at 75 °C for 15 min.

The quenched reaction time points were then precipitated with 2-propanol as described above. For detecting deaminated adenine (inosine) catalyzed by ABEs, the precipitated time points were treated with Endonuclease V as described previously in refs. 20,35. For detecting deaminated cytidine (deoxy-uridine) catalyzed by CBEs, the precipitated time points were treated with USER II (NEB, M5508L) according to manufacturer guidelines. The samples were mixed with equal volume of formamide gel loading buffer (95% formamide, 25 mM EDTA, 0.025% SDS and 0.025% bromophenol blue), heated to 98 °C for 5 min and resolved on denaturing 7.5% Urea-PAGE (19:1, acrylamide:bisacrylamide; National Diagnostics). The reaction was monitored by scanning the gel sequentially with FAM followed by Alexa-647 settings using ChemiDoc Imaging System (Bio-Rad). The intensities of the un-cleaved and cleaved DNA were quantified using ImageJ 1.53 K. Data were fit to a single exponential decay in Prism 9 (GraphPad Prism, v9.4.0) to calculate apparent deamination rates (*k*_app_). Nicking of the substrate DNA by D10A-Nickase of base editor, constant across all base editors assayed, was detected with the 6-FAM fluorophore and used as control to ensure uniformly active recombinant proteins.

For single turnover kinetics of guide RNA-independent ssDNA deamination in vitro, the reaction was set up as described above but with the following modifications: the base editor was not programmed with sgRNA and was incubated with the ATTO-647 labeled ssDNA strand.

For in vitro end-point deamination assay to compare deamination by ABE 8.20, BE4, CABE-T2.17, CABE-T3.155, CBE-T1.14 and CBE-T1.52, the deamination reaction was set up with 1-µM BE RNP and 10-nM dsDNA substrate as described above. Instead of time points, the whole reaction was quenched after 24 h and precipitated as described above. The precipitated reaction was resuspended in water and split into four equal parts: untreated, treated with Endonuclease V as described, treated with USER II as described and treated with human Alkyl Adenine Glycosylase (hAAG; NEB 0313S) followed by AP Endonuclease 1 (APE1; NEB M0282L) according to manufacturer’s instructions. The combination of hAAG and APE1 was used because of our experimental observation that G:U (product of cytosine deamination) is a substrate for EndoV, which was confirmed by NEB (https://www.neb.com/tools-and-resources/selection-charts/dna-repair-enzymes-on-damaged-and-non-standard-bases). EndoV, therefore, could not be used when comparing ABEs, CABEs and CBEs for relative A-to-I and C-to-U deamination activities. hAAG is more specific, and only produced detectable cleavage product for A-to-I but not for C-to-U deamination under the same experimental conditions and thus was used for such comparisons. Following these treatments, the samples were resolved on Urea-PAGE and data were quantified as described above.

### mRNA production of CABE-T, CBE-T and controls used in HEK293T, T cells and primary human hepatocytes

The mRNAs used in this study were produced through in vitro transcription of expression plasmids encoding our editors and controls, in accordance with protocols previously described in ref. 13.

### Isolation of single cells by FACS and whole-genome sequencing

HEK293T cells were transfected via Lipofectamine MessengerMAX (Thermo Fisher Scientific, LMRNA001) with control (Cas9, SPACE, etc.) or editor-encoding mRNA along with synthetic gRNA (special order from Axolabs) targeting a region in β-2-microglobulin (B2M). The sequence of this synthetic guide is as follows (Axolabs-specific syntax): ascsusCACGCUGGAUAGCCUCCGUUUUAGAGCUAGAAAUAGCAAGUUAAAAUAAGGCUAGUCCGUUAUCAACUUGAAAAAGUGGCACCGAGUCGGGUGCusususU

The disruption of B2M upon successful targeting by ABE, CBE or Cas9 at this site has been internally validated. Three days after transfection, cells were dissociated with TrypLE Express, washed with cell staining buffer (Biolegend, 420201) via centrifugation, and resuspended in cell staining buffer containing 1:100 of PE-conjugated antihuman B2M antibody (Biolegend, 316306). After 30 min of incubation on ice in the dark, cells were washed three times with cell staining buffer via centrifugation and strained into standard 5-ml FACS tubes.

Single cells gated as PE-negative were sorted into 96-well plates containing DMEM + 20% FBS + 100 units per ml penicillin/streptomycin (Thermo Fisher Scientific, 15140122). For untreated control, single cells were sorted by live only. Representative gating strategies are provided in Supplementary Fig. 30. After 12 d of culture, gDNA was harvested from cells using the Agencourt DNAdvanced kit (Beckman-Coulter, A48705) in accordance with manufacturer protocols. Confirmation of successful editing of each clone was achieved through targeted amplicon sequencing of the B2M amplicon encompassing the target site. Sequence confirmed gDNA was then submitted to Novogene for library preparation and WGS.

### Isolation and culture of allogeneic human T cells

Human T cells were isolated from leukapheresis products (Leukopaks, HemaCare) by positive selection using CD4 and CD8 MicroBeads (Miltenyi, 130045101 and 130045201). T cells were frozen at 25–50 × 10^6^ cells per ml of Cryostor CS10 (Stemcell Technologies, 1001061). For editing experiments, T cells were thawed in a water bath at 37 °C and then allowed to rest overnight in ImmunoCult-XF T Cell Expansion Medium containing (Stemcell Technologies, 10981) 5% CTS Immune Cell SR, Glutamax, 10 mM HEPES, 1% Penicillin/Streptomycin (Thermo Fisher Scientific, 15140122). The next day, T cells were activated using 25 μl of ImmunoCult Human CD3/CD28/CD2 T Cell Activator (Stemcell Technologies, 10970) per ml of cells at 1 × 10^6^ cells per ml plus 300 IU ml^−1^ of IL-2 (CellGenix, 1420050). Fresh IL-2 was added to T cells every 2–3 d. T cells were cultured at 37 °C and 5% CO_2_.

### Electroporation of human T cells

T cells were transfected 72 h after activation. Cells were resuspended in P3 Primary Cell Nucleofector Solution containing Supplement 1 (Lonza, V4SP-3960). 1 × 10^6^ T cells were edited with 1 μg of synthetic sgRNA (IDT) and 2 μg of editor mRNA in a total volume of 20 µl using P3 96-well Nucleocuvette kit (Lonza, V4SP-3960). The three sgRNAs used are as follows: B2M Exon 2 (B2M Ex.2), pmSTOP C6, CD247 pomSTOP C7 and PD-1 Ex.1 SA C7 are specified in Supplementary Table 3. T cells were electroporated with the 4D-Nucleofector system (Lonza, AAF-1003B and AAF-1003S) using program DH-102. All experiments were performed with two independent T cell donors. For NGS analysis, 1 × 10^5^ T cells per condition at each timepoint were pelleted, supernatant was removed and pellets were resuspended in 50 ml of QuickExtract DNA Extraction buffer (Lucigen, QE09050) and transferred to a PCR plate for targeted amplicon sequencing.

### Flow cytometry of human T cells

Protein knockout was evaluated by flow cytometry 5–6 d post-editing. T cells were stained with fluorophore-conjugated antibodies for TCRα/β (Biolegend, 306718), β2M (Biolegend, 316304) and PD-1 (Biolegend, 367422) via 1:33 dilution in standard PBS. For PD-1 analysis by flow cytometry, T cells were treated with Cell Activation Cocktail (without brefeldin A) (Biolegend) overnight before staining. Events were collected using a MACSQuant Analyzer 16 (Miltenyi). Data were analyzed using the FlowJo software (v10.8.1)

### Generation and maintenance of primary human hepatocytes

Cryogenically frozen primary human hepatocytes (BioIVT) were thawed and plated at a density of 3.5 × 10^5^ cells per well on BioCoat Collagen I 24-well plates (Corning, 354408) and maintained in CP Media supplemented with Torpedo Antibiotic Mix (BioIVT) in accordance with protocols provided by BioIVT. Once PHH monocultures were established overnight, generation of long-lived PHH cultures involved the additional coculturing of 3T3-J2 murine fibroblasts (Kerafast, EF3003) at 2.0 × 10^4^ cells per well to the established PHH monocultures. PHH cocultures were maintained with media changes every 48 h throughout the duration of the study.

### Transfection of primary human hepatocytes

PHH cocultures were transfected 48 h after coculture generation with 3T3-J2 murine fibroblasts. Transfections with mRNA were performed using Lipofectamine MessengerMax (Thermo Fisher Scientific, LMRNA003) in accordance with manufacturers’ protocols, with the following optimized specifics: 1 µg (for saturating conditions) of mRNA encoding for editor and 333 ng of synthetic gRNA (Synthego) were combined in 30 µl of OptiMEM serum reduced medium (Gibco, 31985). A 30 µl 1:15 (Lipofectamine:OptiMEM) mixture was added to the mRNA/gRNA solution with the resulting final mixture left to rest at ambient temperature for 15 min. The entire 60-µl solution was used to treat a well of cocultured primary human hepatocytes. Each study condition was run in triplicate and transfection amounts used were scaled up accordingly. At 9 d post-transfection, the PHH cocultures were lysed with a solution of 10 mM Tris–HCl pH8.0 (Thermo Fisher Scientific, 15568025), 0.05% SDS (Thermo Fisher Scientific, 15553027) and 500 µg proteinase K (Thermo Fisher Scientific, EO0491) at a total of 200 µl per well. Once lysed, lysate was treated at 85 °C for 15 min to inactivate proteinase K. The sequences of sgRNAs used in this study are specified in Supplementary Table 3.

### Protein assays of transfected primary human hepatocytes

PCSK9 protein knockdown quantification was assessed using a Human PCSK9 SimpleStep ELISA kit (Abcam, ab209884) by measuring secreted PCSK9 concentration in supernatant collected every 48 h. Supernatant was ten times diluted using assay buffer, and the assay protocol was run in accordance with the manufacturer’s protocol. LDL-R quantification was assessed using a Human LDL-R SimpleStep ELISA kit (Abcam, ab209884) by measuring secreted LDL-R protein in supernatant collected every 48 h. Both SimpleStep ELISA kits employ an affinity tag labeled capture antibody and a reporter conjugated detector antibody. The capture antibody and detector antibody bind to sample analytes, which are then immobilized to an anti-tag antibody coating the assay well. Both colorimetric ELISA assays are read at an absorbance of 450 nm.

### Reporting summary

Further information on research design is available in the Nature Portfolio Reporting Summary linked to this article.

## Online content

Any methods, additional references, Nature Portfolio reporting summaries, source data, extended data, supplementary information, acknowledgements, peer review information; details of author contributions and competing interests; and statements of data and code availability are available at 10.1038/s41587-022-01611-9.

## Supplementary information

Supplementary Information Supplementary Figs. 1–31, Supplementary Tables 1–5, Supplementary Sequences 1–31 and Supplementary References.

Reporting Summary

Supplementary Data 1 Statistical source data for Supplementary Figs. 2–4, 6, 15, 17–21 and 24–29.

Supplementary Data 2 Gel image source data for Supplementary Fig. 22.

Supplementary Data 3 Gel image source data for Supplementary Fig. 23.

## Data Availability

Next-generation sequencing data underlying all experiments are deposited in the NCBI Sequence Read Archive (SRA) under submission project PRJNA869750. The atomic coordinates and structure factors have been deposited in the PDB as entries: 8E2P, 8E2Q, 8E2R and 8E2S. Source Data are available for Figs. 1, 3–6, Extended Data Fig. 5–7 and Supplementary Figs 2–4, 6, 15, 17–21, 22–29 (including gel image source files). Source data are provided with this paper.

## Source data

Source Data Figs. 1, 3–6 and Extended Data Fig. 5–7 Statistical source data, with excel tabs labeled with the figure or sub-figure number.
Source Data Fig. 5 and Extended Data Fig. 5 Uncropped gels of all gel-based data shown (including those for Supplementary Figs. 22 and 23, which are invariably linked to the main figures and extended figures in question).
Source Data Extended Data Fig. 1 ChemDraw structures file of Extended Data Fig. 1

## Code availability

All software tools used for data analysis are publicly available and were used in a manner as previously described in ref. 13.
