# Supplementary material for: Improved cytosine base editors generated from TadA variants
Source: Nat Biotechnol. 2023 Jan 9;41(5):686–97. doi: 10.1038/s41587-022-01611-9 (PMC10188367; doi:10.1038/s41587-022-01611-9)
Supplement: Supplementary file 1 — Supplementary Figs. 1–31, Supplementary Tables 1–5, Supplementary Sequences 1–31 and Supplementary References. [file 41587_2022_1611_MOESM1_ESM.pdf]

nature biotechnology

Article

https://doi.org/10.1038/s41587-022-01611-9

Improved cytosine base editors generated from TadA variants

In the format provided by the authors and unedited

Table of Contents

Supplementary Figure 1: Amino acid sequence identities of candidate CABE-T1 variants isolated from directed evolution round 1……………4

Supplementary Figure 2: Median C·G to T·A and A·T to G·C conversion of select CABE-T and CBE-T editors from two rounds of directed evolution and two rounds of structure-guided combinatorial screens, genomic sites #1-11……………5

Supplementary Figure 3: Median C·G to T·A and A·T to G·C conversion of select CABE-T and CBE-T editors from two rounds of directed evolution and two rounds of structure-guided combinatorial screens, genomic sites #12-2……………6

Supplementary Figure 4: Initial screen of CABE-T1 editors from the first round of directed evolution in HEK293T cells……………7

Supplementary Figure 5: Amino acid sequence identities of candidate CABE-T2 variants isolated from the directed evolution round 2……………8

Supplementary Figure 6: Initial screen of CABE-T2 editors from the second round of directed evolution in HEK293T cells..9

Supplementary Figure 7: Stereo view of the interactions between ssDNA and TadA*8.20 active site residues……………10

Supplementary Figure 8: Stereo view of TadA*8.20 in a complex with ssDNA containing the transition-state analog 2-deoxy-8-azanebularine……………11

Supplementary Figure 9: Structural comparisons between TadA*8.20 and related structures……………12

Supplementary Figure 10: Stereo view of TADAC-T1.17 in a complex with ssDNA containing the transition-state analog 2-deoxy-8-azanebularine……………13

Supplementary Figure 11: Stereo view of TADAC-T1.14 with water bound to the zinc ion……………14

Supplementary Figure 12: Stereo view of TADAC-T1.19 with water bound to the zinc ion……………15

Supplementary Figure 13: Amino acid sequence identities of candidate CABE-T3 variants in the first structure-guided combinatorial screening, variants #1-100……………16

Supplementary Figure 14: Amino acid sequence identities of candidate CABE-T3 variants in the first structure-guided combinatorial screening, variants #101-199……………17

Supplementary Figure 15: Screen of CABE-T3 editors from the first structure-guided combinatorial screen in HEK293T cells……………18

Supplementary Figure 16: Amino acid sequence identities of candidate CBE-T variants in the second structure-guided combinatorial screen……………19

Supplementary Figure 17: Screen of CBE-T editors from the second structure-guided combinatorial screen in HEK293T cells……………20

Supplementary Figure 18: Fold change in C-to-T and A-to-G editing rates between CBE-T1 variants and ABE8.20 at each target site position……………21

Supplementary Figure 19: Comparison of a representative set of CABE-Ts and CBE-Ts to the previously reported ABE-P48R-UGI editor variant……………22

Supplementary Figure 20: Evaluation of a representative set of CABE-Ts and CBE-Ts utilizing *S. aureus* Cas9 nickase in HEK293T cells……………23

Supplementary Figure 21: Median C·G to T·A and A·T to G·C conversion of CABE-T and CBE-T constructs at eight genomic sites in HEK293T transfected with mRNA at saturating conditions……………24

Supplementary Figure 22: *In vitro* kinetic analysis of ABE8.20, BE4, & CBE-T1.14 acting on the same substrate DNA**…..**25

Table of Contents Continued

Supplementary Figure 23: *In vitro* relative nicking of substrate DNA by ABE8.20, BE4, & CBE-T1.14 acting on the same dsDNAsubstrate……...26

Supplementary Figure 24: Dose-response curve of percent maximum C·G to T·A or A·T to G·C conversion in transfections of HEK293T with varying concentrations of mRNA ……………………………………………………………………….…....27

Supplementary Figure 25: Median C·G to T·A and A·T to G·C conversion of CABE-T and CBE-T constructs at eight genomic sites in HEK293T transfected with mRNA at sub-saturating conditions ……………………………….………..…...28

Supplementary Figure 26: Median Indel Frequencies of CABE-T & CBE-T editor variants characterized in this study….....29

Supplementary Figure 27: Median C·G to T·A or A·T to G·C conversion of representative CABE-T and CBE-T editor variants across eight genomic sites at each target site position ….…………………………………………………………...30

Supplementary Figure 28: Guide RNA-dependent off-target C·G to T·A conversion of editors evaluated in this study...….31

Supplementary Figure 29: Guide RNA-dependent off-target A·T to G·C conversion of editors evaluated in this study......32

Supplementary Figure 30: Representative examples of gates used to flow sort live single B2M-positive and B2M-negative cells for whole genome sequencing………………………………………………………………………………..……....33

Supplementary Figure 31: Representative flow gates used for assessment of protein knockdown in T cells……...........34

Supplementary Table 1: List of substitutions in TadA*8.20 relative to wild-type *Escherichia coli* TadA (EcTadA)………...35

Supplementary Table 2: Data collection and refinement statistics for crystal structures ……………..…………………..…36

Supplementary Table 3: Sequences of sgRNAs used for transfection ………………..……………..……………...…37-38

Supplementary Table 4: Primers used to amplify genomic sites for HTS…………………………………………..…...…39-41

Supplementary Table 5: The number of residues (1-167) and nucleotides (1-13) visualized in the structures.…………..…42

Supplementary Sequence 1: Inactivated chloramphenicol resistance gene used for directed evolution round 1….……..43

Supplementary Sequence 2: Inactivated chloramphenicol resistance gene used for directed evolution round 1………..44

Supplementary Sequence 3-31: Amino Acid sequences of editors characterized in this study……………………..….45-59

Supplementary References……….………….……….……….……….……….……….……….……...………………………...….60

Amino Acid position	2	8	13	17	27	47	48	49	67	76	77	82	84	96	107	112	115	118	119	127	142	162	165
TadA*8.20	**S**	**H**	**R**	**T**	**E**	**R**	**A**	**I**	**G**	**Y**	**D**	**S**	**F**	**H**	**R**	**G**	**G**	**M**	**D**	**N**	**A**	**A**	**S**
TadA*8.19										**I**													
**TadAC-1.1**					H					I			M										
**TadAC-1.2**					H			K		I													
**TadAC-1.3**					S			K		I													
**TadAC-1.4**					S			K		I												N	
**TadAC-1.5**					K																		
**TadAC-1.6**					K														N				
**TadAC-1.7**					H					I													
**TadAC-1.8**					S			K	W														
**TadAC-1.9**								T	W					N									
**TadAC-1.10**					C					I									N				
**TadAC-1.11**			G		Q															K			
**TadAC-1.12**				A	H			M		I								L					
**TadAC-1.13**								Q		I							M						
**TadAC-1.14**	H							K		I						H							
**TadAC-1.15**						S									C								
**TadAC-1.16**		Q						Q		I													
**TadAC-1.17**				A			G					T									E		
**TadAC-1.18**					G																		
**TadAC-1.19**					G			N															
**TadAC-1.20**					G						G												P

Supplementary Figure 1 | Amino acid sequence identities of candidate TadAC deaminase in CABE-T1 variants isolated from directed evolution round 1. Mutational identities of the TadA* deaminase from each candidate CABE-T1 variant are specified by residue position and amino acid change relative to the TadA* of ABE8.19-m and ABE8.20-m[1]

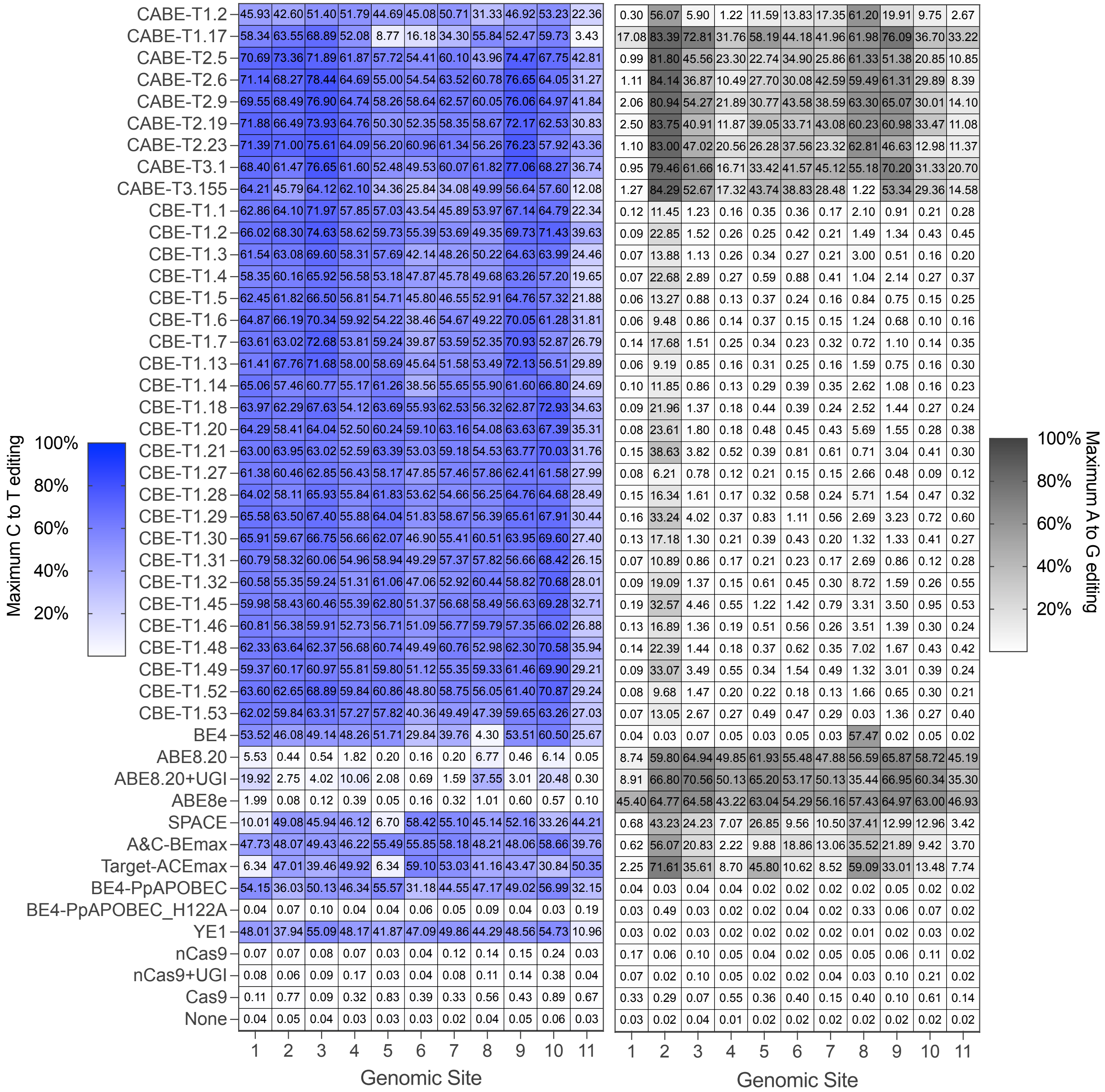

Supplementary Figure 2 | Median C·G to T·A and A·T to G·C conversion of select CABE-T and CBE-T editors from two rounds of directed evolution and two rounds of structure-guided combinatorial screens, genomic sites #1-11. Color maps depict the median of percent maximum A·T to G·C or C·G to T·A base conversion at targeted genomic loci in HEK293T cells transfected with plasmid encoding editor (or control) + plasmid encoding sgRNA. Median values were derived from n = 3 (genomic sites 5-8 and 10-11) or 4 (genomic sites 1-4 and 9) independent biological replicates performed on different days. Samples under the sequencing read threshold (<5000 mapped reads) were excluded.

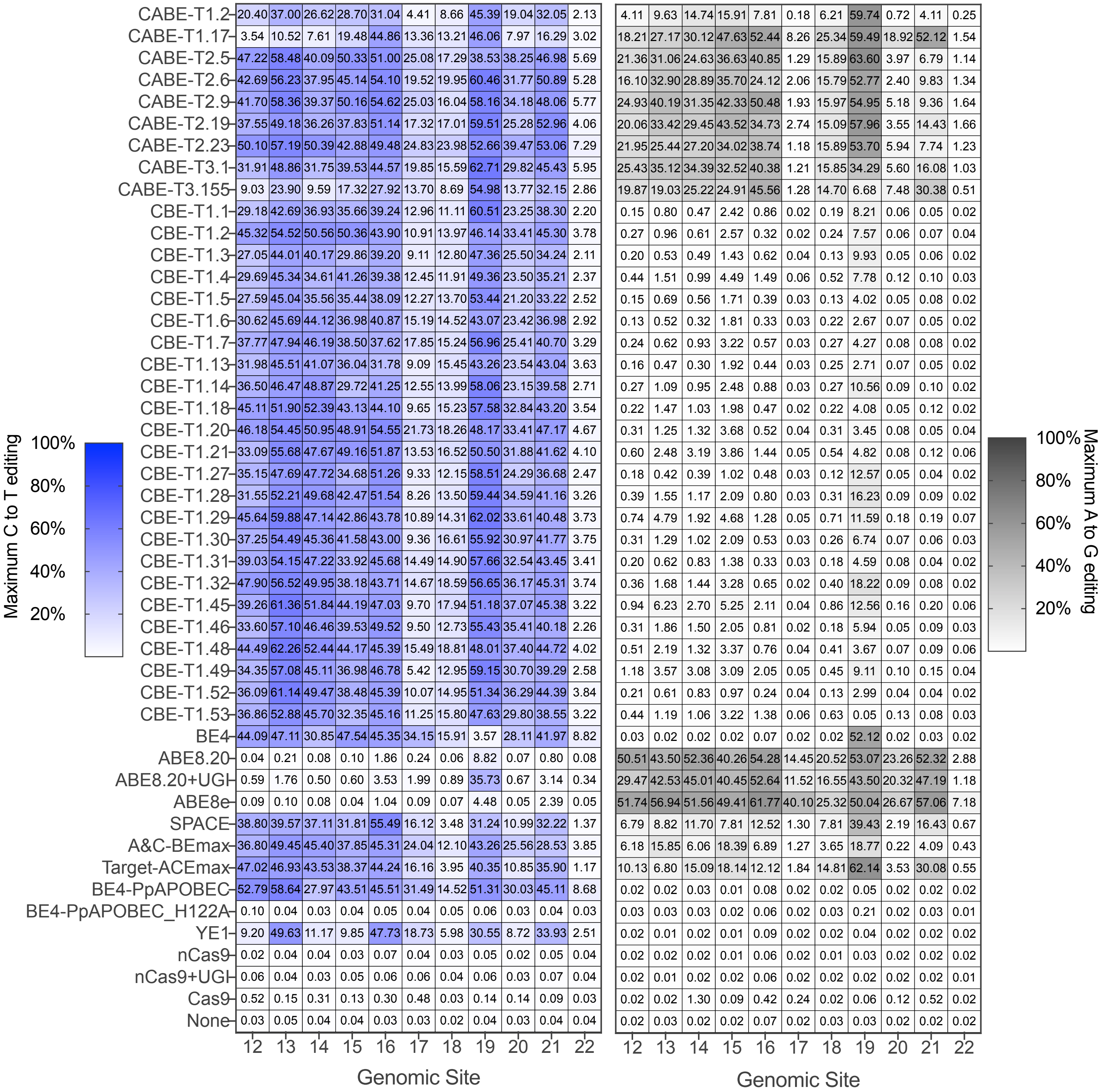

Supplementary Figure 3 | Median C·G to T·A and A·T to G·C conversion of select CABE-T and CBE-T editors from two rounds of directed evolution and two rounds of structure-guided combinatorial screens, genomic sites #12-22. Color maps depict the median of percent maximum A·T to G·C or C·G to T·A base conversion at targeted genomic loci in HEK293T cells transfected with plasmid encoding editor (or control) + plasmid encoding sgRNA. Median values were derived from n = 3 independent biological replicates performed on different days. Samples under the sequencing read threshold (<5000 mapped reads) were excluded.

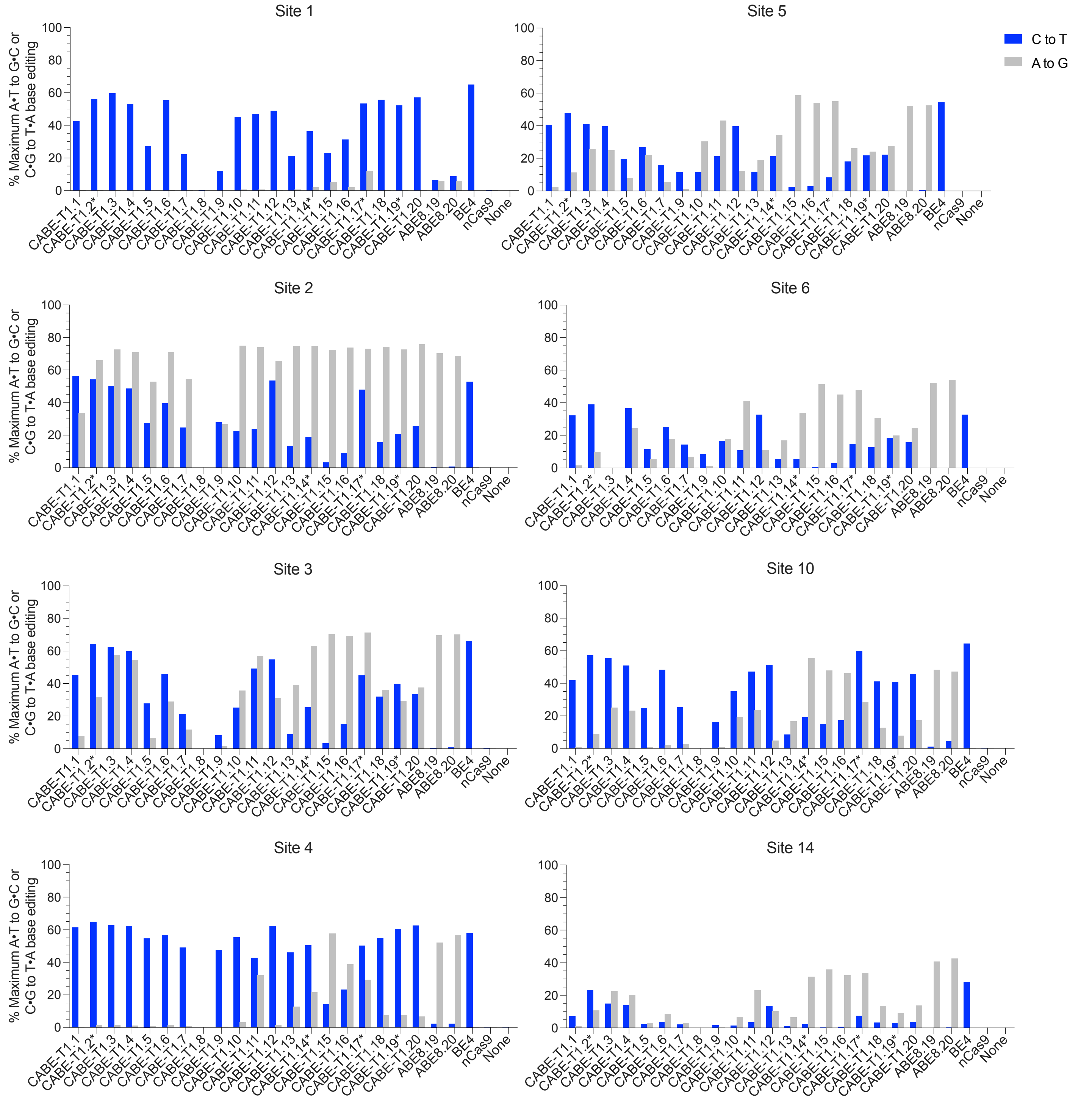

Supplementary Figure 4 | Initial screen of CABE-T1 editors from the first round of directed evolution in HEK293T cells. a-b, Percent maximum C·G to T·A and A·T to G·C conversion at targeted genomic loci in HEK293T cells transfected with plasmid encoding CABE-T1 variants from the first round of directed evolution, plus plasmid encoding guide RNA targeting the site specified. 500 ng of editor plasmid and 100 ng gRNA plasmid were used. Y axis is expressed as the percentage of sequencing reads mapped as C·G to T·A or A·T to G·C. Variants chosen for further characterization or development are marked *. The data in this screen was derived from n = 1 replicate.

Amino Acid Position	4	6	17	23	76	77	100	111	114	119	122	127	143	147	158	159	162	166
TADAC-1.2	V	F	T	R	I	D	G	T	A	D	H	N	A	R	A	Q	A	T
TADAC-2.1	K								C									
TADAC-2.2	K					G												
TADAC-2.3		Y					A				R							
TADAC-2.4	T				R						G							
TADAC-2.5		Y			W													
TADAC-2.6		Y								N								
TADAC-2.7		Y							C									
TADAC-2.8		Y																
TADAC-2.9	K				W						T							
TADAC-2.10		G			R		K											
TADAC-2.11		H									N							
TADAC-2.12									C									
TADAC-2.13		Y			H						R							I
TADAC-2.14												P						
TADAC-2.15				Q	R													
TADAC-2.16					H						R				V			
TADAC-2.17		Y						H										
TADAC-2.18					W													
TADAC-2.19								H			G						C	
TADAC-2.20													E					
TADAC-2.21		Y			R													
TADAC-2.22			W		H						G				V			
TADAC-2.23	S				Y								E			S		
TADAC-2.24												I					Q	

Supplementary Figure 5 | Amino acid sequence identities of candidate T$_{AD}$AC deaminase CABE-T2 variants isolated from directed evolution round 2. Mutational identities of the T$_{AD}$AC deaminase from each candidate CABE-T2 variant are specified by residue position and amino acid change relative to the TadA* of CABE-T1.2 (basis of the library).

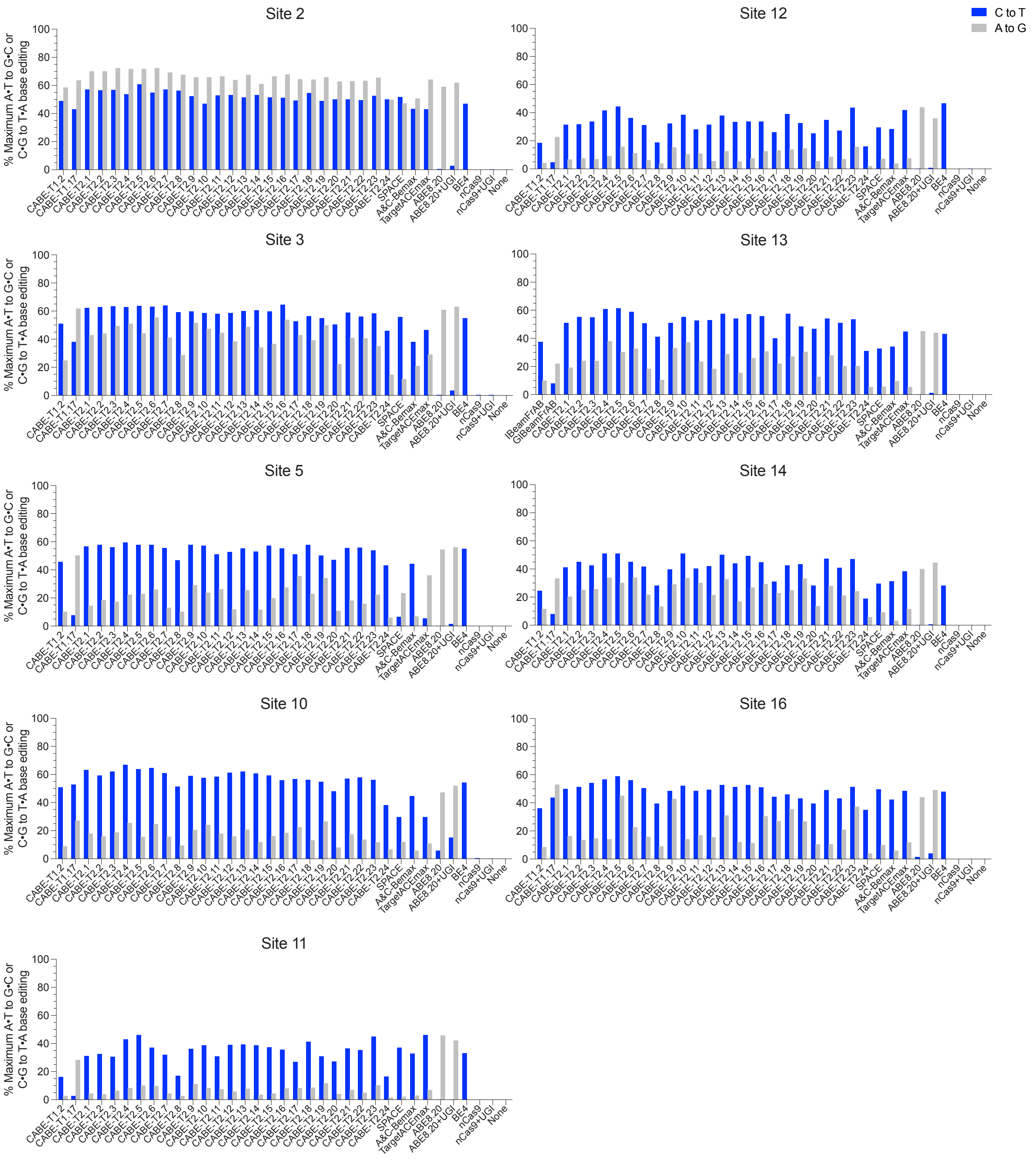

Supplementary Figure 6 | Initial screen of CABE-T2 editors from the second round of directed evolution in HEK293T cells. Percent maximum C·G to T·A and A·T to G·C conversion at targeted genomic loci in HEK293T cells transfected with plasmid encoding CABE-T2 variants from the second round of directed evolution round, plus plasmid encoding guide RNA targeting the site specified. 500 ng of editor plasmid and 100 ng gRNA plasmid were used. The data in this screen was derived from n = 1 replicate.

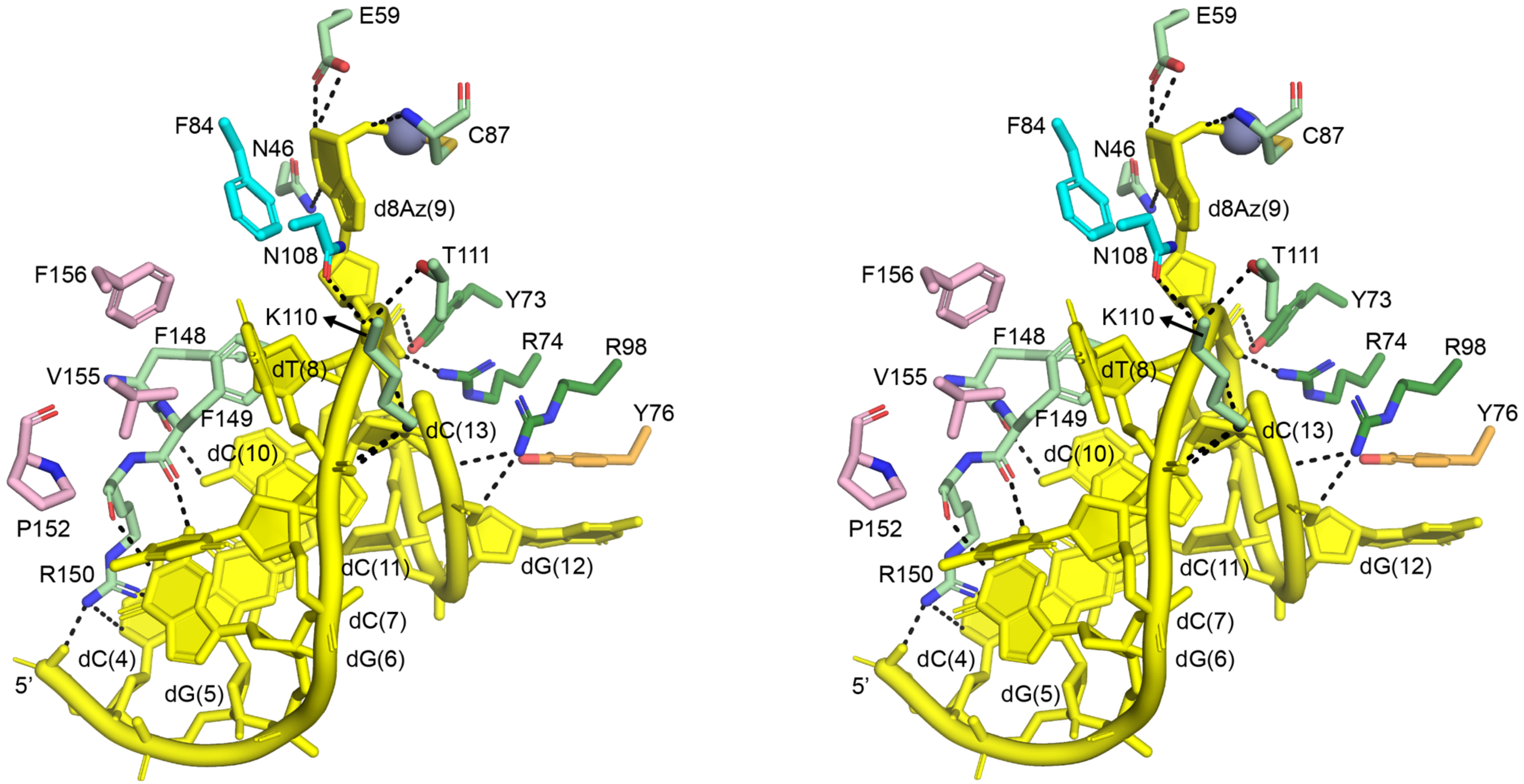

Supplementary Figure 7 | Stereo view of the interactions between ssDNA and TadA*8.20 active site residues. The ssDNA is shown in yellow. The residues from chains A and B are shown in dark and light green, respectively. The zinc ion is shown in a gray sphere. The protein surface, C-terminal, and active site substitutions are shown in orange (chain A), pink (chain B), and cyan (chain B), respectively. The hydrogen bonds are shown as black dashed lines.

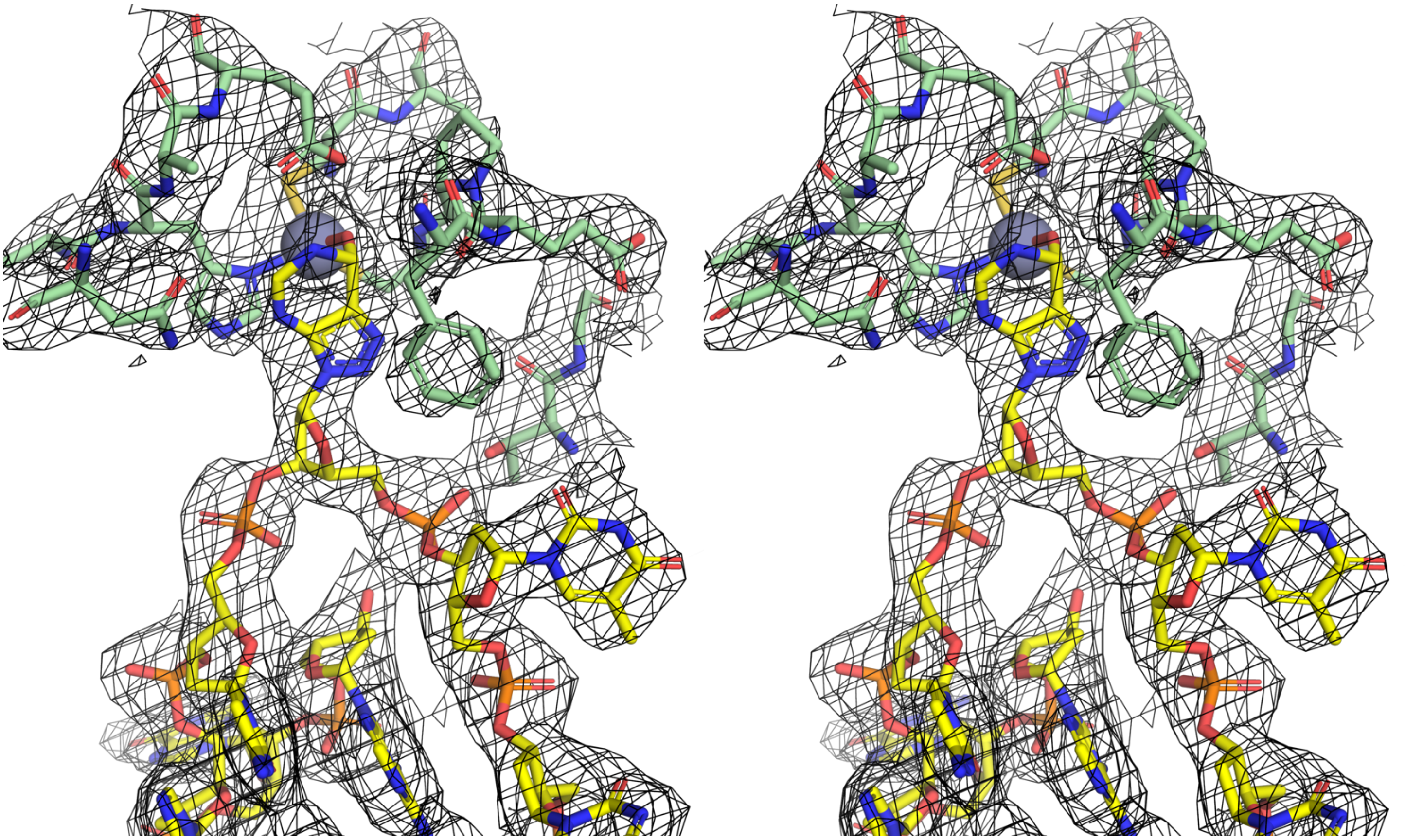

Supplementary Figure 8 | Stereo view of TadA*8.20 (light green) in a complex with ssDNA (yellow) containing the transition-state analog 2-deoxy-8-azanebularine. The $2F_o - F_c$ electron density map (black mesh) is contoured at 1.0 sigma. The zinc ion is shown as a gray sphere.

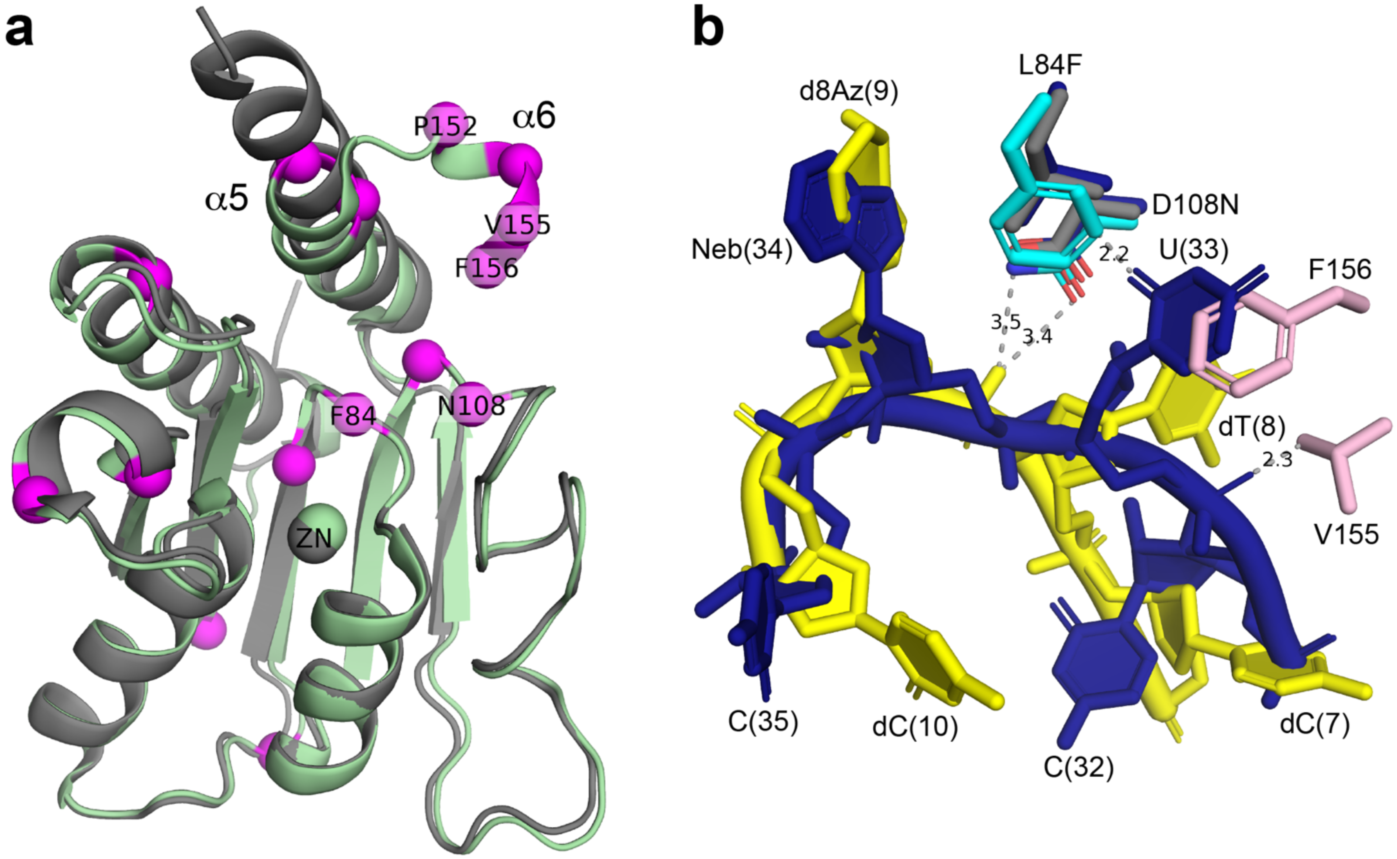

Supplementary Figure 9 | Structural comparisons between TadA*8.20 and related structures. a, Superposition between TadA*8.20 (light green) and wild-type *Escherichia coli* TadA (EcTadA) (gray; PDB 1Z3A[2]) monomers. The structures of TadA*8.20 and EcTadA are highly similar (RMSD of 0.7-Å for all of the Ca atoms), with main structural differences in the C-terminal α5-helix. This helix is broken into two α-helices (α5 and α6) in TadA*8.20 mainly due to the R152P substitution, as previously observed for TadA*8e[3]. The TadA*8.20 substitutions relative to EcTadA (**Supplementary Table 1**) are shown as magenta spheres. **b**, Superposition between TadA*8.20 with ssDNA (yellow), *Staphylococcus aureus* TadA (SaTadA) with the anticodon loop of tRNA (dark blue; PDB 2B3J[4]) and EcTadA without tRNA. The TadA*8.20 residues V155 (pink) and F156 (pink), present in the C-terminal α6-helix, interact with ssDNA base dT(8) but would make steric clashes with tRNA bases C(32) and U(33). Also, the TadA*8.20 residue F84 (cyan), present in the active site, would make steric clash with tRNA U(33) base (2.2-Å). These findings suggest that changes in the C-terminal secondary structure due to substitutions accumulated during evolution and a substitution in the active site may contribute to the evolved protein binding ssDNA over tRNA. The Tad*8.20 residues interacting with ssDNA are shown in pink (C-terminal) and cyan (active site). The residue N108 H-bonds (gray dashed lines) to the backbone of the target nucleobase d8Az(9) in TadA*8.20. The L84 and D108 residues of SaTadA and EcTadA are shown in dark blue and gray, respectively.

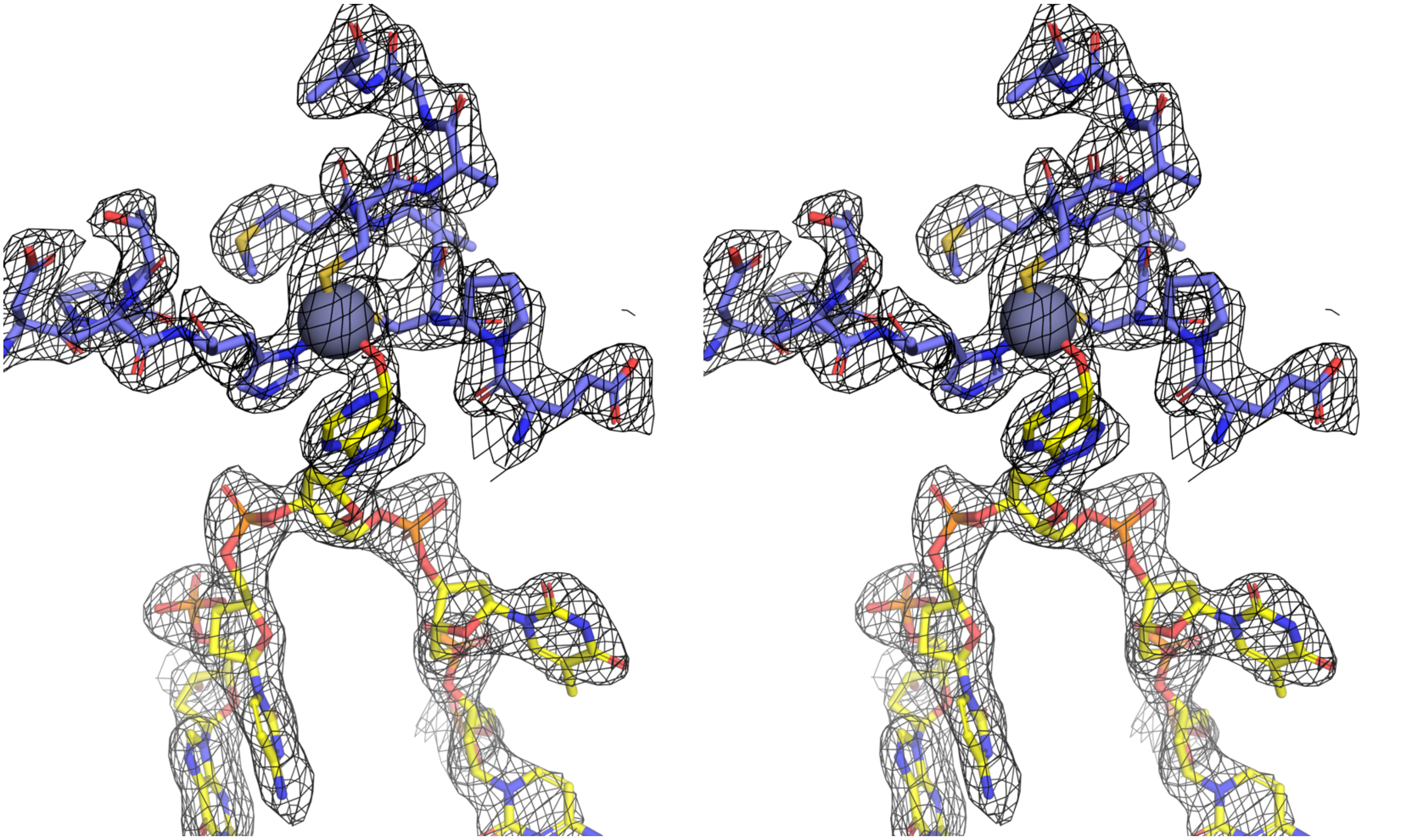

Supplementary Figure 10 | Stereo view of T_{AD}AC-T1.17 (slate blue) in a complex with ssDNA (yellow) containing the transition-state analog 2-deoxy-8-azanebularine. The $2F_o - F_c$ electron density map (black mesh) is contoured at 1.5 sigma. The zinc ion is shown as a gray sphere.

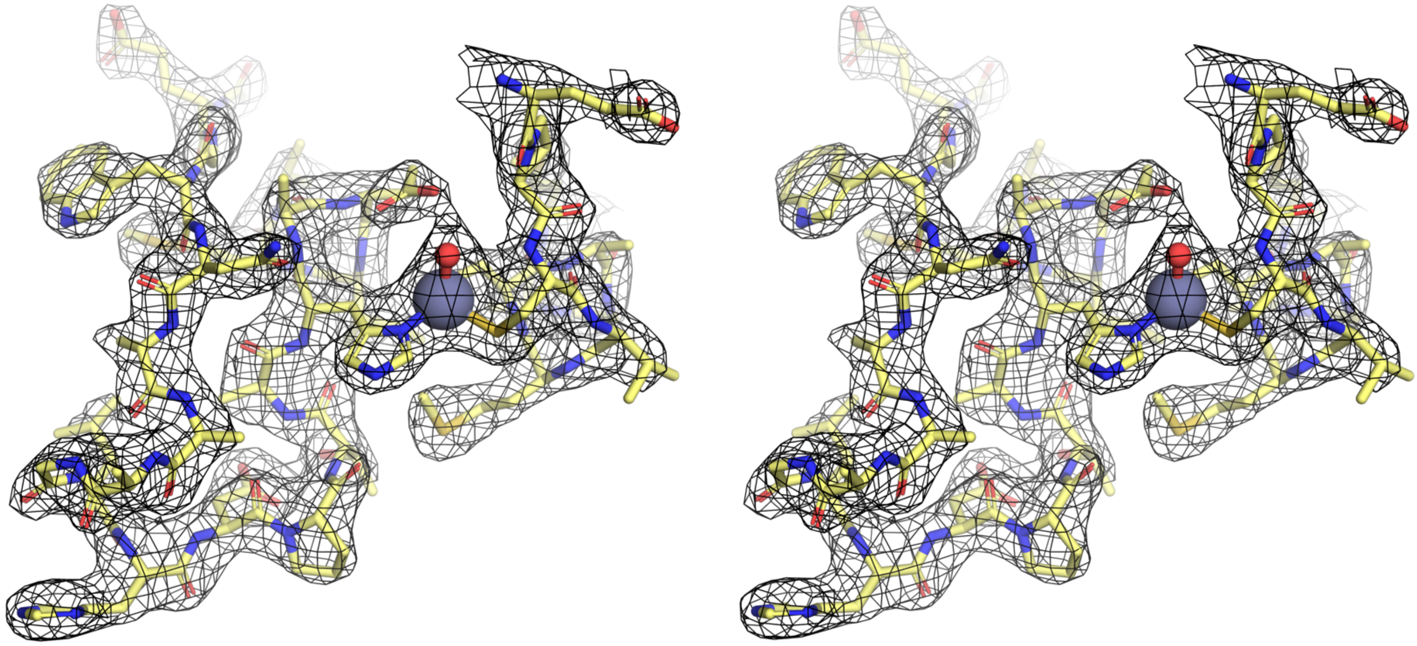

Supplementary Figure 11 | Stereo view of T_{AD}AC-T1.14 (light yellow) with water (red sphere) bound to the zinc ion (gray sphere). The $2F_o - F_c$ electron density map (black mesh) is contoured at 1.5 sigma.

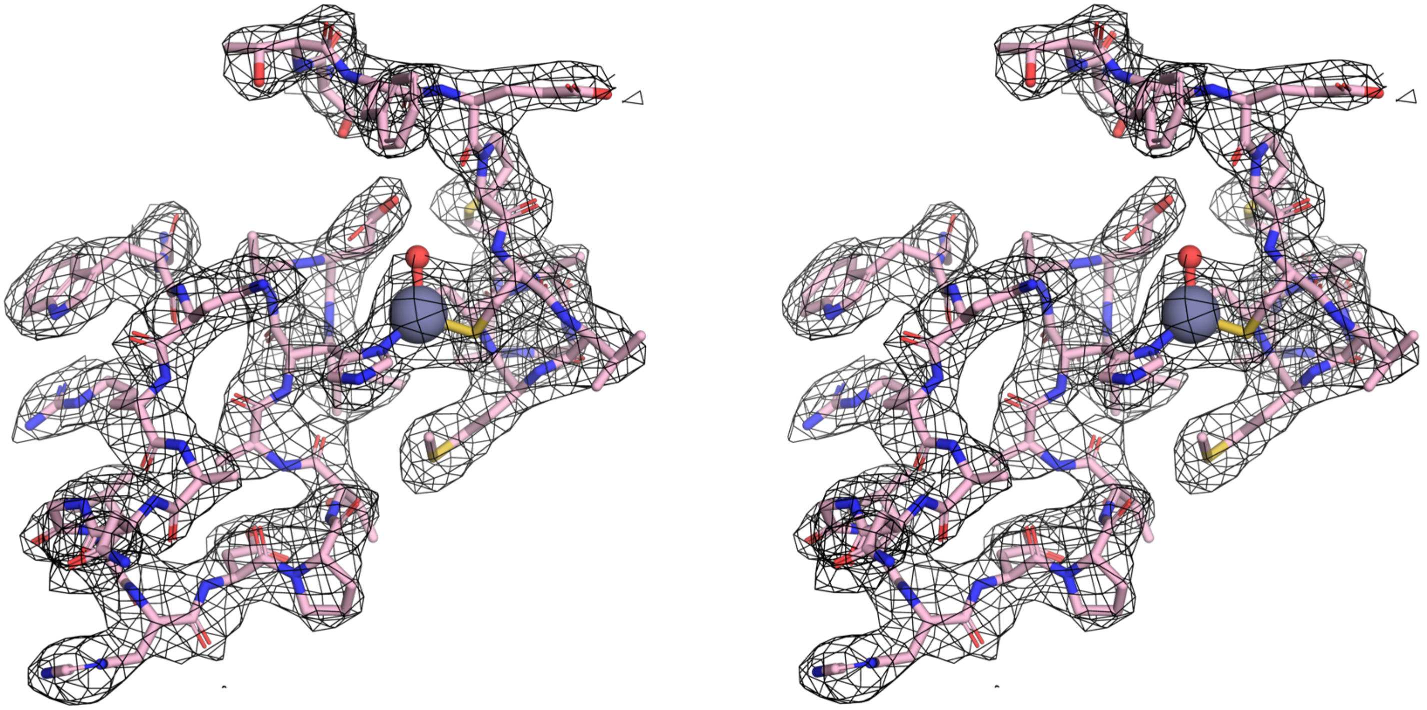

Supplementary Figure 12 | Stereo view of T$_{AD}$AC-T1.19 (light pink) with water (red sphere) bound to the zinc ion (gray sphere). The $2F_o - F_c$ electron density map (black mesh) is contoured at 1.0 sigma.

Amino Acid Position	27	29	30	49	82	84	107	112	115	142
**Tad*8.20**	**E**	**P**	**V**	**I**	**S**	**F**	**R**	**G**	**G**	**A**
**Mutation**	**G/S/H**	**G/A/K**	**I/L/F**	**K**	**T**	**L/A**	**C**	**H**	**M**	**E**
**TADAC-3.1**	S			K	T					
**TADAC-3.2**	S			K	T		C			
**TADAC-3.3**	S			K	T			H		
**TADAC-3.4**	S			K	T				M	
**TADAC-3.5**	S			K	T					E
**TADAC-3.6**	S			K	T		C	H		
**TADAC-3.7**	S			K	T		C		M	
**TADAC-3.8**	S			K	T		C			E
**TADAC-3.9**	S			K	T			H		E
**TADAC-3.10**	S			K	T				M	E
**TADAC-3.11**	S			K	T		C	H	M	E
**TADAC-3.12**	S		I	K	T					
**TADAC-3.13**	S		I	K	T		C			
**TADAC-3.14**	S		I	K	T			H		
**TADAC-3.15**	S		I	K	T				M	
**TADAC-3.16**	S		I	K	T					E
**TADAC-3.17**	S		I	K	T		C	H		
**TADAC-3.18**	S		I	K	T		C		M	
**TADAC-3.19**	S		I	K	T		C			E
**TADAC-3.20**	S		I	K	T			H		E
**TADAC-3.21**	S		I	K	T				M	E
**TADAC-3.22**	S		I	K	T		C	H	M	E
**TADAC-3.23**	S		L	K	T					
**TADAC-3.24**	S		L	K	T		C			
**TADAC-3.25**	S		L	K	T			H		
**TADAC-3.26**	S		L	K	T				M	
**TADAC-3.27**	S		L	K	T					E
**TADAC-3.28**	S		L	K	T		C	H		
**TADAC-3.29**	S		L	K	T		C		M	
**TADAC-3.30**	S		L	K	T		C			E
**TADAC-3.31**	S		L	K	T			H		E
**TADAC-3.32**	S		L	K	T				M	E
**TADAC-3.33**	S		L	K	T		C	H	M	E
**TADAC-3.34**	S		F	K	T	A				
**TADAC-3.35**	S		F	K	T	A	C			
**TADAC-3.36**	S		F	K	T	A		H		
**TADAC-3.37**	S		F	K	T	A			M	
**TADAC-3.38**	S		F	K	T	A				E
**TADAC-3.39**	S		F	K	T	A	C	H		
**TADAC-3.40**	S		F	K	T	A	C		M	
**TADAC-3.41**	S		F	K	T	A	C			E
**TADAC-3.42**	S		F	K	T	A		H		E
**TADAC-3.43**	S		F	K	T	A			M	E
**TADAC-3.44**	S		F	K	T	A	C	H	M	E
**TADAC-3.45**	S			K	T	L				
**TADAC-3.46**	S			K	T	L	C			
**TADAC-3.47**	S			K	T	L		H		
**TADAC-3.48**	S			K	T	L			M	
**TADAC-3.49**	S			K	T	L				E
**TADAC-3.50**	S			K	T	L	C	H		
**TADAC-3.51**	S			K	T	L	C		M	
**TADAC-3.52**	S			K	T	L	C			E
**TADAC-3.53**	S			K	T	L		H		E
**TADAC-3.54**	S			K	T	L			M	E
**TADAC-3.55**	S			K	T	L	C	H	M	E
**TADAC-3.56**	S		I	K	T	L				
**TADAC-3.57**	S		I	K	T	L	C			
**TADAC-3.58**	S		I	K	T	L		H		
**TADAC-3.59**	S		I	K	T	L			M	
**TADAC-3.60**	S		I	K	T	L				E
**TADAC-3.61**	S		I	K	T	L	C	H		
**TADAC-3.62**	S		I	K	T	L	C		M	
**TADAC-3.63**	S		I	K	T	L	C			E
**TADAC-3.64**	S		I	K	T	L		H		E
**TADAC-3.65**	S		I	K	T	L			M	E
**TADAC-3.66**	S		I	K	T	L	C	H	M	E
**TADAC-3.67**	S	G		K	T					
**TADAC-3.68**	S	G		K	T		C			
**TADAC-3.69**	S	G		K	T			H		
**TADAC-3.70**	S	G		K	T				M	
**TADAC-3.71**	S	G		K	T					E
**TADAC-3.72**	S	G		K	T		C	H		
**TADAC-3.73**	S	G		K	T		C		M	
**TADAC-3.74**	S	G		K	T		C			E
**TADAC-3.75**	S	G		K	T			H		E
**TADAC-3.76**	S	G		K	T				M	E
**TADAC-3.77**	S	G		K	T		C	H	M	E
**TADAC-3.78**		G		K	T					
**TADAC-3.79**		G		K	T		C			
**TADAC-3.80**		G		K	T			H		
**TADAC-3.81**		G		K	T				M	
**TADAC-3.82**		G		K	T					E
**TADAC-3.83**		G		K	T		C	H		
**TADAC-3.84**		G		K	T		C		M	
**TADAC-3.85**		G		K	T		C			E
**TADAC-3.86**		G		K	T			H		E
**TADAC-3.87**		G		K	T				M	E
**TADAC-3.88**		G		K	T		C	H	M	E
**TADAC-3.89**		K		K	T					
**TADAC-3.90**		K		K	T		C			
**TADAC-3.91**		K		K	T			H		
**TADAC-3.92**		K		K	T				M	
**TADAC-3.93**		K		K	T					E
**TADAC-3.94**		K		K	T		C	H		
**TADAC-3.95**		K		K	T		C		M	
**TADAC-3.96**		K		K	T		C			E
**TADAC-3.97**		K		K	T			H		E
**TADAC-3.98**		K		K	T				M	E
**TADAC-3.99**		K		K	T		C	H	M	E
**TADAC-3.100**		K	I	K	T					

Supplementary Figure 13 | Amino acid sequence identities of candidate TADAC deaminase in CABE-T3 variants in the first structure-guided combinatorial screening, variants #1-100. Mutational identities of the TADAC deaminase from each candidate CABE-T3 variant are specified by residue position and amino acid change relative to the TadA* of ABE8.20. Mutational priorities were determined via crystal structures of TadA* from select CABE-T1 variants.

Amino Acid Position	27	29	30	49	82	84	107	112	115	142
Tad*8.20	E	P	V	I	S	F	R	G	G	A
Mutation	G/S/H	G/A/K	I/L/F	K	T	L/A	C	H	M	E
TADAC-3.101		K	I	K	T		C			
TADAC-3.102		K	I	K	T			H		
TADAC-3.103		K	I	K	T				M	
TADAC-3.104		K	I	K	T					E
TADAC-3.105		K	I	K	T		C	H		
TADAC-3.106		K	I	K	T		C		M	
TADAC-3.107		K	I	K	T		C			E
TADAC-3.108		K	I	K	T			H		E
TADAC-3.109		K	I	K	T				M	E
TADAC-3.110		K	I	K	T		C	H	M	E
TADAC-3.111		K		K	T	L				
TADAC-3.112		K		K	T	L	C			
TADAC-3.113		K		K	T	L		H		
TADAC-3.114		K		K	T	L			M	
TADAC-3.115		K		K	T	L				E
TADAC-3.116		K		K	T	L	C	H		
TADAC-3.117		K		K	T	L	C		M	
TADAC-3.118		K		K	T	L	C			E
TADAC-3.119		K		K	T	L		H		E
TADAC-3.120		K		K	T	L			M	E
TADAC-3.121		K		K	T	L	C	H	M	E
TADAC-3.122		K	I	K	T	L				
TADAC-3.123		K	I	K	T	L	C			
TADAC-3.124		K	I	K	T	L		H		
TADAC-3.125		K	I	K	T	L			M	
TADAC-3.126		K	I	K	T	L				E
TADAC-3.127		K	I	K	T	L	C	H		
TADAC-3.128		K	I	K	T	L	C		M	
TADAC-3.129		K	I	K	T	L	C			E
TADAC-3.130		K	I	K	T	L		H		E
TADAC-3.131		K	I	K	T	L			M	E
TADAC-3.132		K	I	K	T	L	C	H	M	E
TADAC-3.133	G			K	T					
TADAC-3.134	G			K	T		C			
TADAC-3.135	G			K	T			H		
TADAC-3.136	G			K	T				M	
TADAC-3.137	G			K	T					E
TADAC-3.138	G			K	T		C	H		
TADAC-3.139	G			K	T		C		M	
TADAC-3.140	G			K	T		C			E
TADAC-3.141	G			K	T			H		E
TADAC-3.142	G			K	T				M	E
TADAC-3.143	G			K	T		C	H	M	E
TADAC-3.144	H			K	T					
TADAC-3.145	H			K	T		C			
TADAC-3.146	H			K	T			H		
TADAC-3.147	H			K	T				M	
TADAC-3.148	H			K	T					E
TADAC-3.149	H			K	T		C	H		
TADAC-3.150	H			K	T		C		M	

Amino Acid Position	27	29	30	49	82	84	107	112	115	142
Tad*8.20	E	P	V	I	S	F	R	G	G	A
Mutation	G/S/H	G/A/K	I/L/F	K	T	L/A	C	H	M	E
TADAC-3.151	H			K	T		C			E
TADAC-3.152	H			K	T			H		E
TADAC-3.153	H			K	T				M	E
TADAC-3.154	H			K	T		C	H	M	E
TADAC-3.155	S				T					
TADAC-3.156	S				T		C			
TADAC-3.157	S				T			H		
TADAC-3.158	S				T				M	
TADAC-3.159	S				T					E
TADAC-3.160	S				T		C	H		
TADAC-3.161	S				T		C		M	
TADAC-3.162	S				T		C			E
TADAC-3.163	S				T			H		E
TADAC-3.164	S				T				M	E
TADAC-3.165	S				T		C	H	M	E
TADAC-3.166		A			T					
TADAC-3.167		A			T		C			
TADAC-3.168		A			T			H		
TADAC-3.169		A			T				M	
TADAC-3.170		A			T					E
TADAC-3.171		A			T		C	H		
TADAC-3.172		A			T		C		M	
TADAC-3.173		A			T		C			E
TADAC-3.174		A			T			H		E
TADAC-3.175		A			T				M	E
TADAC-3.176		A			T		C	H	M	E
TADAC-3.177	S		I		T					
TADAC-3.178	S		I		T		C			
TADAC-3.179	S		I		T			H		
TADAC-3.180	S		I		T				M	
TADAC-3.181	S		I		T					E
TADAC-3.182	S		I		T		C	H		
TADAC-3.183	S		I		T		C		M	
TADAC-3.184	S		I		T		C			E
TADAC-3.185	S		I		T			H		E
TADAC-3.186	S		I		T				M	E
TADAC-3.187	S		I		T		C	H	M	E
TADAC-3.188		A	I		T	L				
TADAC-3.189		A	I		T	L	C			
TADAC-3.190		A	I		T	L		H		
TADAC-3.191		A	I		T	L			M	
TADAC-3.192		A	I		T	L				E
TADAC-3.193		A	I		T	L	C	H		
TADAC-3.194		A	I		T	L	C		M	
TADAC-3.195		A	I		T	L	C			E
TADAC-3.196		A	I		T	L		H		E
TADAC-3.197		A	I		T	L			M	E
TADAC-3.198		A	I		T	L	C	H	M	E
TADAC-3.199	S	A	L	K	T	L	C	H	M	E

Supplementary Figure 14 | Amino acid sequence identities of candidate T$_{AD}$AC deaminase in CABE-T3 variants in the first structure-guided combinatorial screening, variants #101-199. Mutational identities of the T$_{AD}$AC deaminase from each candidate CABE-T3 variant are specified by residue position and amino acid change relative to the TadA* of ABE8.20. Mutational priorities were determined via crystal structures of TadA* from select CABE-T1 variants.

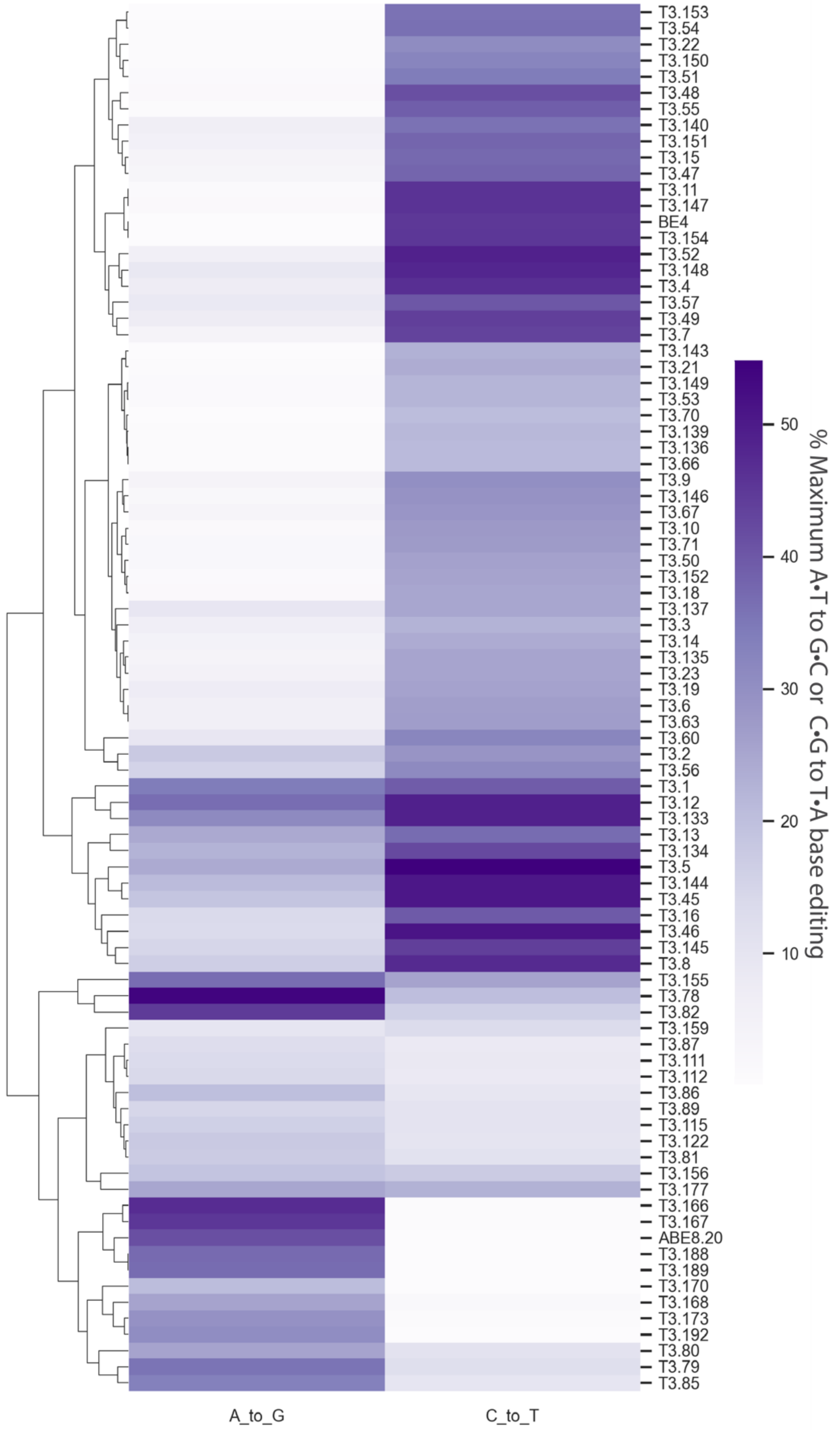

Supplementary Figure 15 | Screen of CABE-T3 editors for base editing activity in HEK293T cells. Colormap of median percent maximum C·G to T·A and A·T to G·C conversion at 6 targeted genomic loci (sites 3, 5, 12, 13, 14, & 16, see Supplementary Table 3) in HEK293T cells transfected with plasmid encoding CABE-T3 variants from the first structure-guided combinatorial screen, plus plasmid encoding guide RNA targeting the site specified. Variants with >20% A-to-G or C-to-T are included. 500ng of editor plasmid and 100ng gRNA plasmid were used. Variants are clustered by similarities in ratio of C-to-T and A-to-G editing. The data in this screen was derived from n = 1 replicate.

Amino Acid Position	6	27	49	76	77	82	107	112	114	115	119	122	127	142	143
TadA8.20	F	E	I	Y	D	S	R	G	A	G	D	H	N	A	A
TADC-1.1	Y	H	K	W		T	C	H		M				E	
TADC-1.2	Y	H	K		G	T	C	H		M				E	
TADC-1.3	Y	H	K			T	C	H	C	M				E	
TADC-1.4	Y	H	K			T	C	H		M	N			E	
TADC-1.5	Y	H	K			T	C	H		M		G		E	
TADC-1.6	Y	H	K			T	C	H		M			P	E	
TADC-1.7	Y	H	K			T	C	H		M				E	E
TADC-1.8	Y	H	K			T	C	H		M				A	E
TADC-1.9	Y	H	K	W	G	T	C	H		M				E	
TADC-1.10	Y	H	K	W		T	C	H	C	M				E	
TADC-1.11	Y	H	K	W		T	C	H		M	N			E	
TADC-1.12	Y	H	K	W		T	C	H		M		G		E	
TADC-1.13	Y	H	K	W		T	C	H		M			P	E	
TADC-1.14	Y	H	K	W		T	C	H		M				E	E
TADC-1.15	Y	H	K	W		T	C	H		M				A	E
TADC-1.16	Y	H	K		G	T	C	H	C	M				E	
TADC-1.17	Y	H	K		G	T	C	H		M	N			E	
TADC-1.18	Y	H	K		G	T	C	H		M		G		E	
TADC-1.19	Y	H	K		G	T	C	H		M			P	E	
TADC-1.20	Y	H	K		G	T	C	H		M				E	E
TADC-1.21	Y	H	K		G	T	C	H		M				A	E
TADC-1.22	Y	H	K			T	C	H	C	M	N			E	
TADC-1.23	Y	H	K			T	C	H	C	M		G		E	
TADC-1.24	Y	H	K			T	C	H	C	M			P	E	
TADC-1.25	Y	H	K			T	C	H		M	N	G		E	
TADC-1.26	Y	H	K			T	C	H		M	N		P	E	
TADC-1.27	Y	H	K			T	C	H		M		G	P	E	
TADC-1.28	Y	H	K	W	G	T	C	H	C	M				E	
TADC-1.29	Y	H	K	W	G	T	C	H		M	N			E	
TADC-1.30	Y	H	K	W	G	T	C	H		M		G		E	
TADC-1.31	Y	H	K	W	G	T	C	H		M			P	E	
TADC-1.32	Y	H	K	W	G	T	C	H		M				E	E
TADC-1.33	Y	H	K	W	G	T	C	H		M				A	E
TADC-1.34	Y	H	K	W		T	C	H	C	M	N			E	
TADC-1.35	Y	H	K	W		T	C	H	C	M		G		E	
TADC-1.36	Y	H	K	W		T	C	H	C	M			P	E	
TADC-1.37	Y	H	K	W		T	C	H	C	M				E	E
TADC-1.38	Y	H	K	W		T	C	H	C	M				A	E
TADC-1.39	Y	H	K	W		T	C	H		M	N	G		E	
TADC-1.40	Y	H	K	W		T	C	H		M	N		P	E	
TADC-1.41	Y	H	K	W		T	C	H		M		G	P	E	
TADC-1.42	Y	H	K	W		T	C	H	C	M	N	G		E	
TADC-1.43	Y	H	K	W		T	C	H	C	M	N		P	E	
TADC-1.44	Y	H	K	W		T	C	H	C	M		G	P	E	
TADC-1.45	Y	H	K	W	G	T	C	H	C	M	N			E	
TADC-1.46	Y	H	K	W	G	T	C	H	C	M		G		E	
TADC-1.47	Y	H	K	W	G	T	C	H	C	M			P	E	
TADC-1.48	Y	H	K	W	G	T	C	H	C	M				E	E
TADC-1.49	Y	H	K	W	G	T	C	H	C	M				A	E
TADC-1.50	Y	H	K	W	G	T	C	H	C	M	N	G		E	
TADC-1.51	Y	H	K	W	G	T	C	H	C	M	N		P	E	
TADC-1.52	Y	H	K	W	G	T	C	H	C	M		G	P	E	
TADC-1.53	Y	H	K	W		T	C	H	C	M	N		P	E	E
TADC-1.54	Y	H	K	W		T	C	H	C	M	N	G	P	A	E
TADC-1.55	Y	H	K	W	G	T	C	H	C	M	N		P	E	E
TADC-1.56	Y	H	K	W	G	T	C	H	C	M	N	G	P	A	E

Supplementary Figure 16 | Amino acid sequence identities of candidate $T_{AD}C$ deaminase CBE-T variants in the second structure-guided combinatorial screen. Mutational identities of the $T_{AD}C$ deaminase from each candidate CBE-T1 variant are identified by residue position and amino acid change relative to the TadA* of ABE8.20. Mutations inherited from CABE-T3.154 (basis of the library) are colored orange.

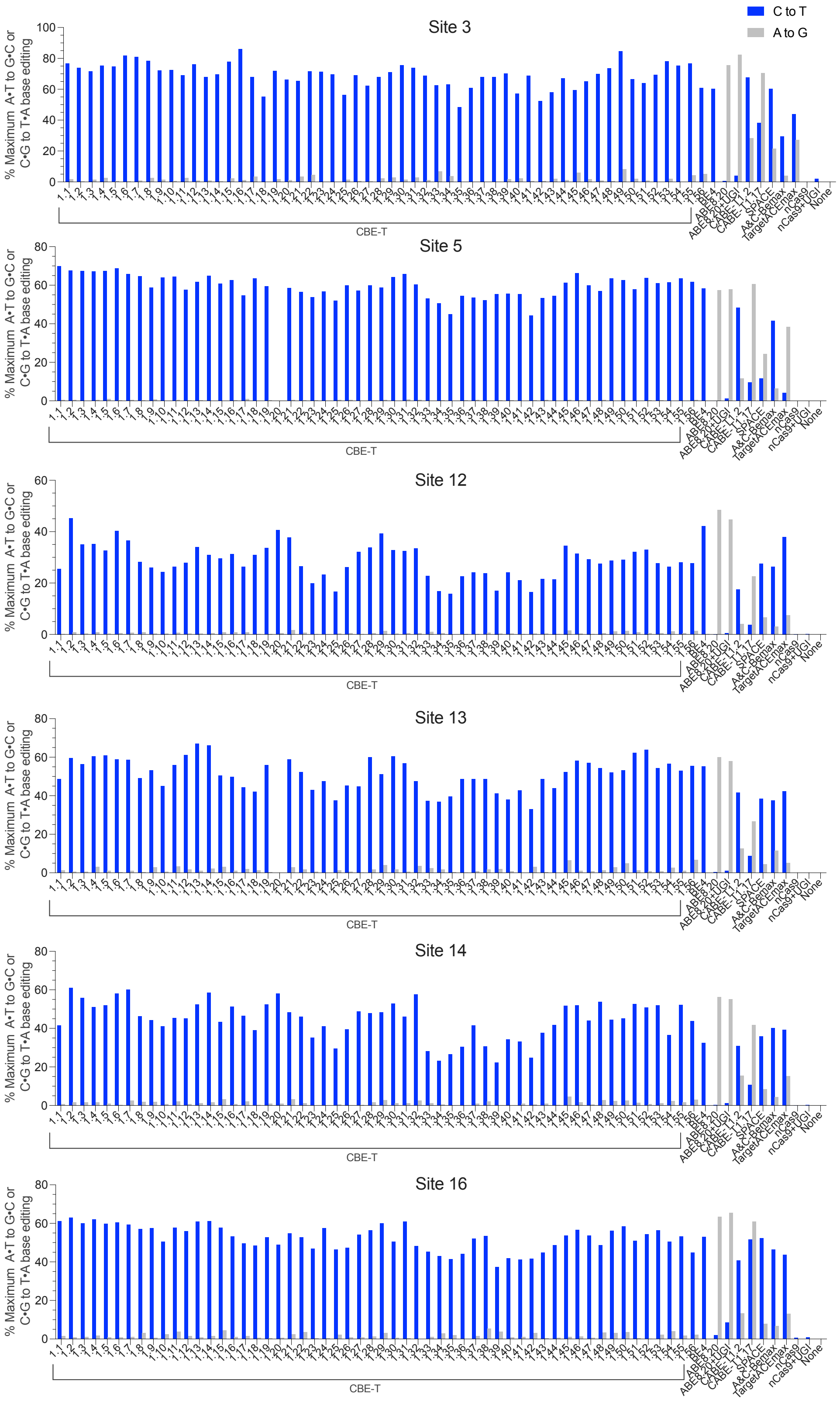

Supplementary Figure 17 | Screen of CBE-T editors from the second structure-guided combinatorial screen in HEK293T cells. Percent maximum C·G to T·A and A·T to G·C conversion at targeted genomic loci in HEK293T cells transfected with a plasmid encoding CBE-T variants from the second structure-guided combinatorial screen, plus plasmid encoding guide RNA targeting the site specified. 500 ng of editor plasmid and 100 ng gRNA plasmid were used. Samples with insufficient (<5000) sequencing reads were excluded. The data in this screen was derived from n = 1 replicate.

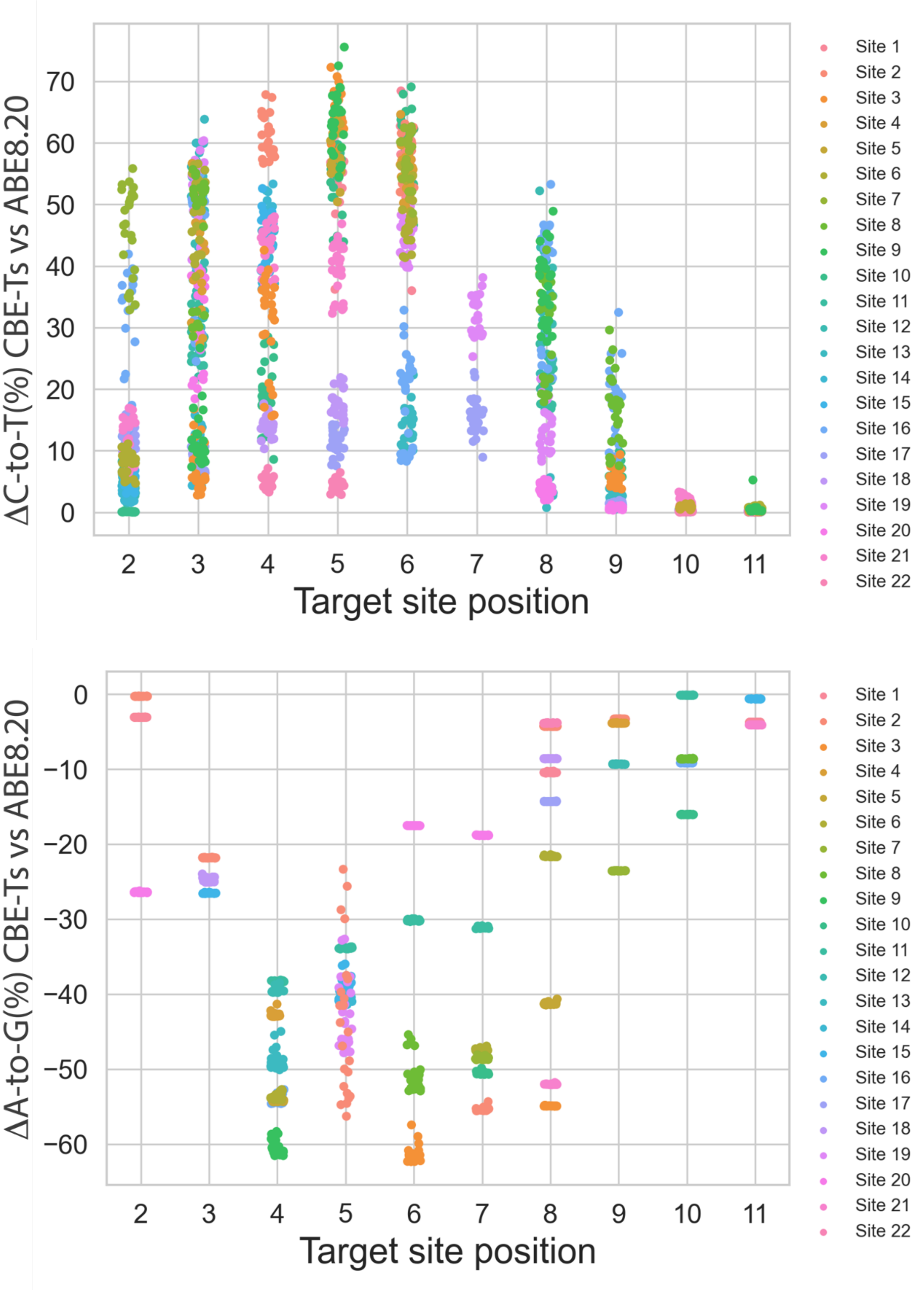

Supplementary Figure 18 | Fold change in C-to-T and A-to-G editing rates between CBE-T1 variants and ABE8.20 at each target site position. X-axis denotes target site position at the protospacer, with the PAM defined as positions 21-23. Y-axis denotes change in percent C-to-T(top) or A-to-G(bottom) base editing of CBE-T1 variants relative to that of ABE8.20. Values were determined from plasmid transfections at 22 genomic sites with n = 3 - 4 biological replicates conducted on different days.

a

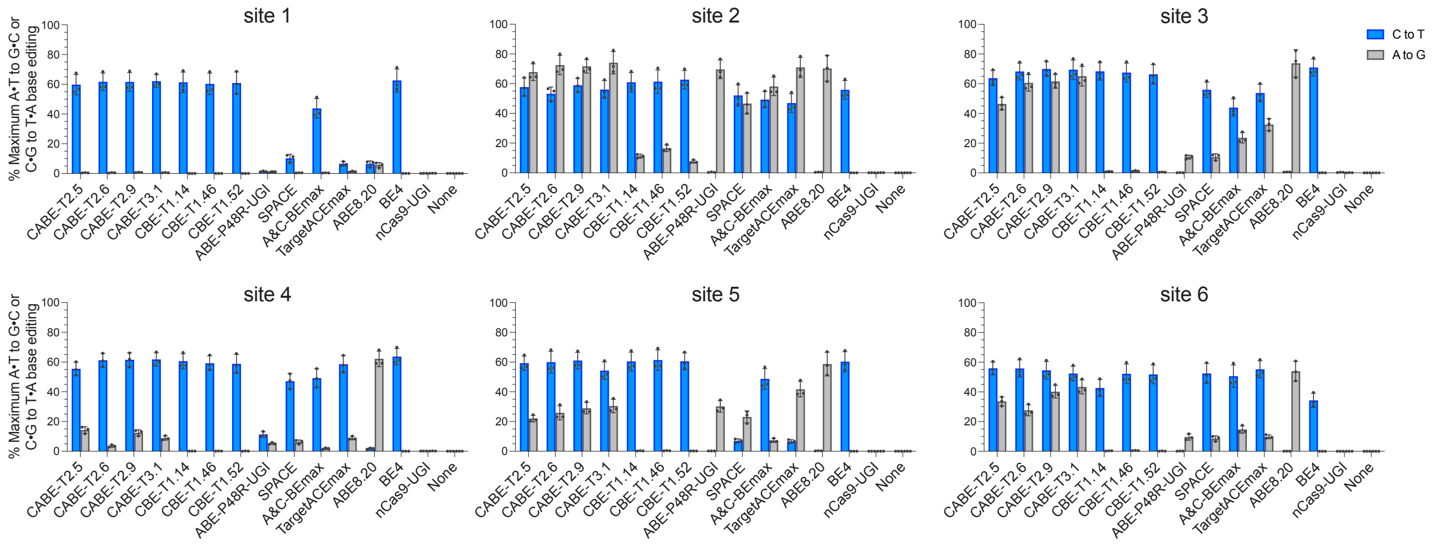

site 1: GAGTC$_5$C$_6$GAGCAGAAGAAGAA
site 2: GAAC$_4$AC$_6$AAAGCATAGACTGC
site 3: GGCC$_4$C$_5$AGACTGAGCACGTGA
site 4: GTCA**TC$_6$**TTAGTCATTACCTG
site 5: GTGGGAC$_5$TGATCCCTTAATGTG
site 6: GCCC$_3$AGC$_6$AATTCACTGTGAAG

b

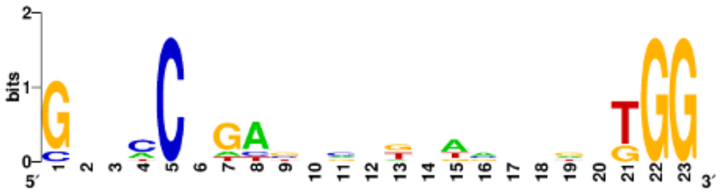

WebLogo of target sites containing C5 (N=7)

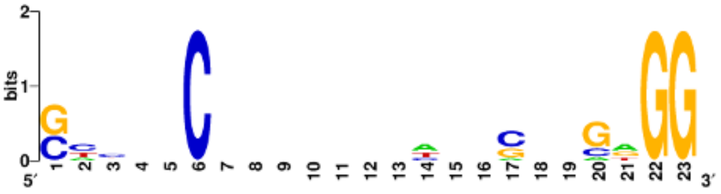

WebLogo of target sites containing C6 (N=9)

Supplementary Figure 19 | Comparison of a representative set of CABE-Ts and CBE-Ts to the previously reported ABE-P48R-UGI. a) Percent maximum C•G to T•A and A•T to G•C conversion of targeted genomic loci in HEK293T cells transfected with plasmid encoding a representative subset of CABE-T variants, CBE-T variants, and the previously described ABE-P48R-UGI[5], plus plasmid encoding guide RNA targeting the site specified. 500 ng of editor plasmid and 100 ng gRNA plasmid were used. Values and error bars in this screen reflect the mean and SD from n = 3 independent biological replicates. **b)** WebLogo plots are included for target site sequences containing cytosines at positions 5 or 6 within the protospacer (NGG = positions 21-23) and showing high editing efficiency at the position. Nearly all CBE-Ts and CABE editors have C•G to T•A editing >20% at these positions.

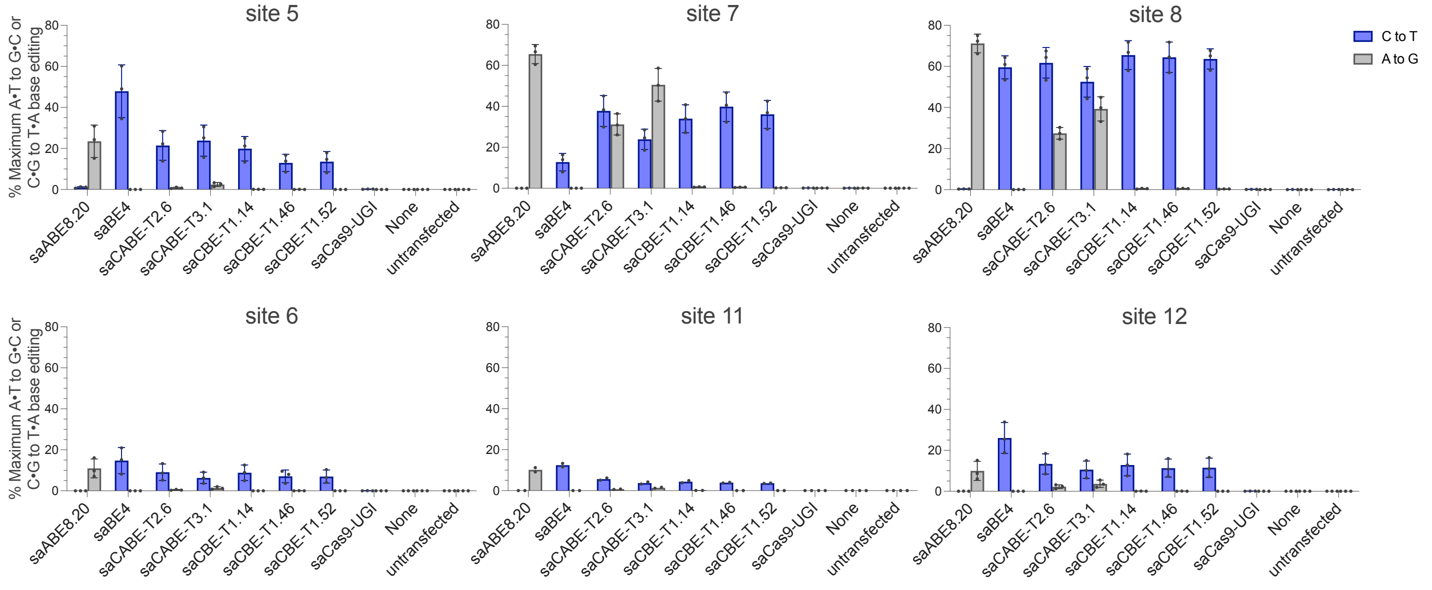

Supplementary Figure 20 | Evaluation of a representative set of CABE-Ts and CBE-Ts utilizing *S. aureus* Cas9 nickase in HEK293T cells. Percent maximum C•G to T•A and A•T to G•C conversion of targeted genomic loci in HEK293T cells transfected with plasmid encoding a representative subset of CABE-T and CBE-T variants, plus controls, that utilize an *S. aureus* Cas9 nickase in lieu of *S. pyogenes* Cas9 nickase. The *S. aureus* Cas9 nickase used has been previously shown to have compatibility with ABE editors[6]. Plasmid encoding guide RNA targeting the site specified was also included. 500 ng of editor plasmid and 100 ng gRNA plasmid were used. *S. aureus* Cas9 nickase (NGGRRT) versions of the gRNAs targeting these genomic loci utilize a saCas9-compatible scaffold, and are detailed in Supplementary Table 3. The data in this screen was derived from n = 3 independent biological replicates with values and error bars reflecting the mean and SD, except for site 11, where the data was derived from n = 2 independent biological replicates.

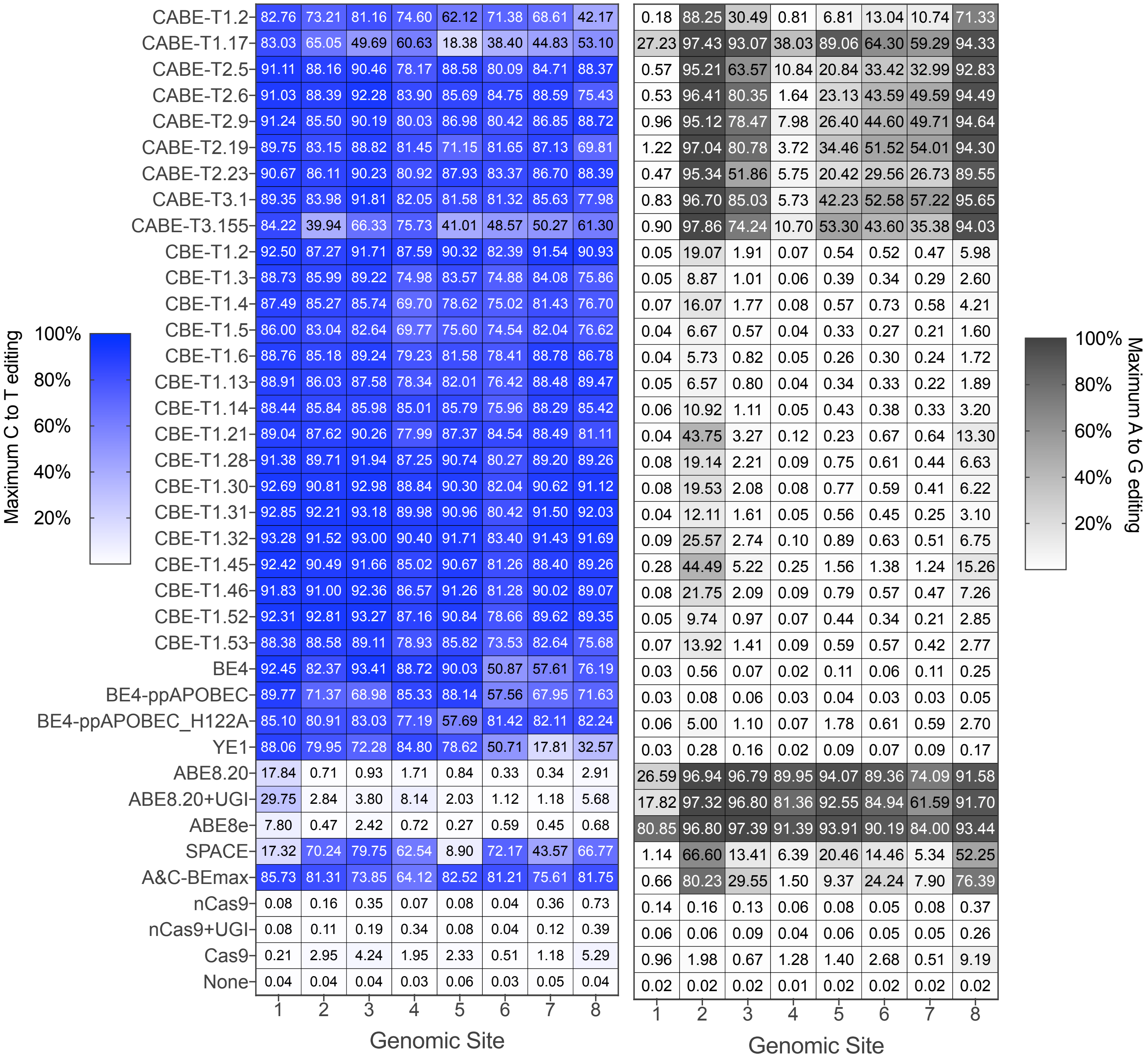

Supplementary Figure 21 | Median C·G to T·A and A·T to G·C conversion of CABE-T and CBE-T constructs at eight genomic sites in HEK293T transfected with mRNA at saturating conditions. Color maps depict the median of percent maximum A·T to G·C or C·G to T·A base conversion at targeted genomic loci in HEK293T cells transfected with mRNA encoding editor (or control) plus synthetic sgRNA at saturating conditions (500ng mRNA + 100ng synthetic guide). Median values were derived from n = 4 independent biological replicates performed on different days. Samples under the sequencing read threshold (<5000 mapped reads) were excluded.

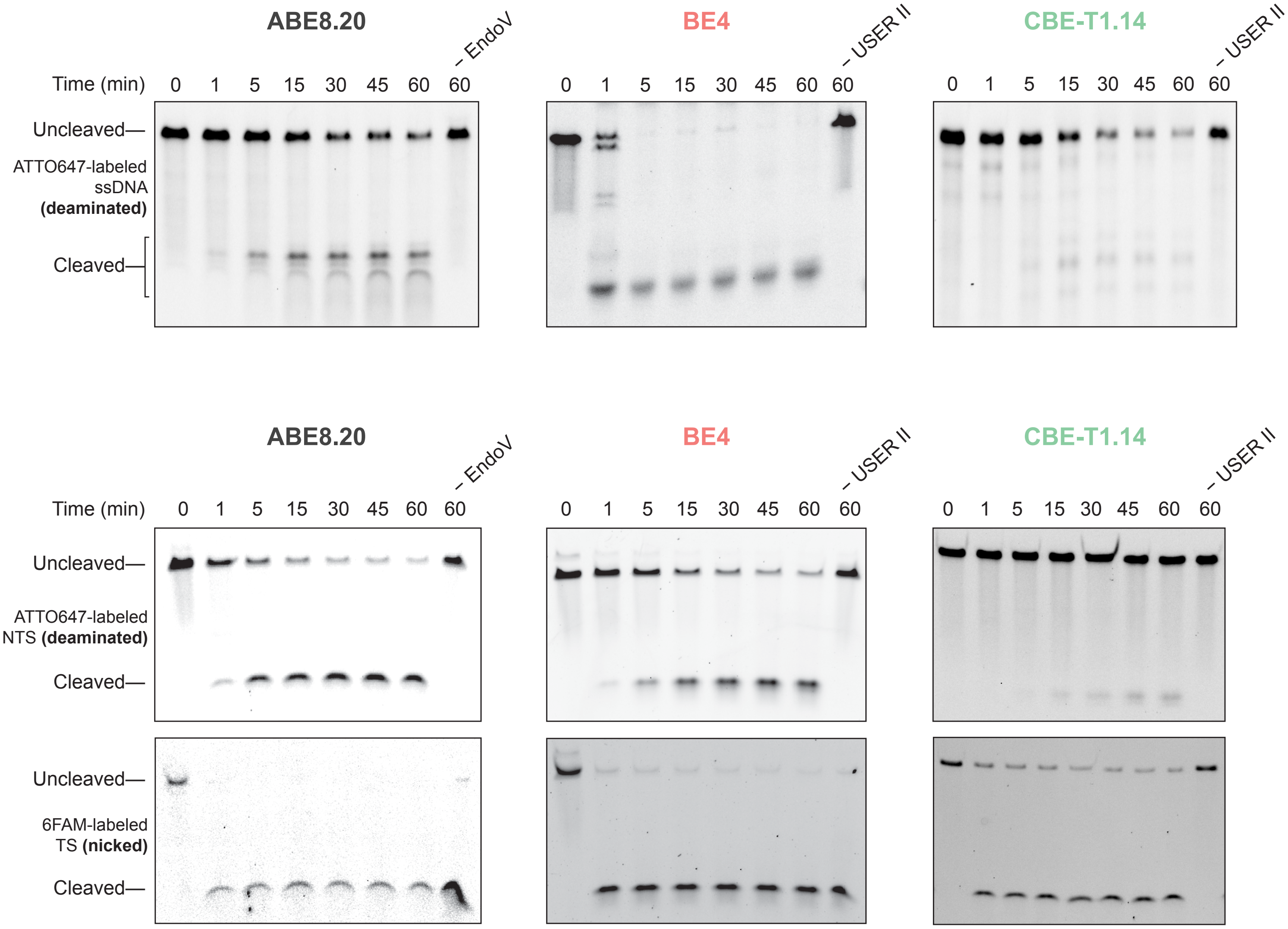

Supplementary Figure 22 | *In vitro* kinetic analysis of ABE8.20, BE4, and CBE-T1.14 acting on the same substrate DNA. Top, Representative gel images showing time dependence of deamination by unprogrammed BE4, ABE8.20, and CBE-T1.14 acting on ssDNA substrate labeled with ATTO647 dye. **Bottom**, Representative gel images showing time dependence of deamination (top) and nicking (bottom) by guide-RNA programmed BE4, ABE8.20, and CBE-T1.14 acting on dsDNA substrate. Top and bottom are different scans of the same gel, scanned first in the fluorescein channel followed by Alexa 647 channel. The same DNA strand undergoes deamination in **top** and **bottom**. The experiment was done thrice with similar results. For gel images of all replicates, see gel source data.

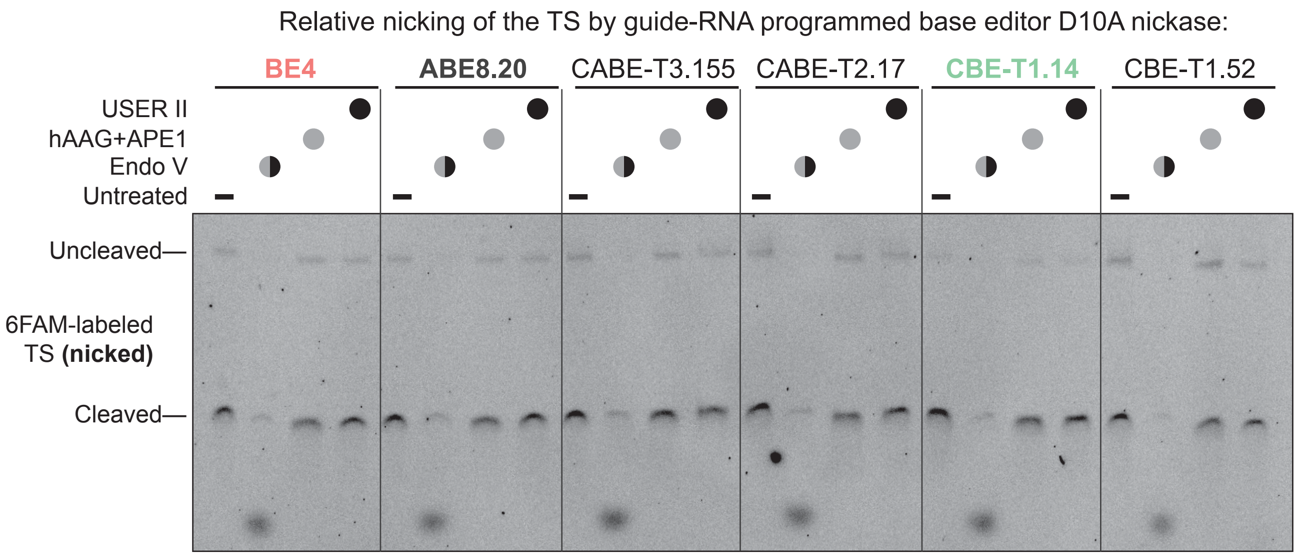

Supplementary Figure 23 | *In vitro* relative nicking of substrate DNA by ABE8.20, BE4, & CBE-T1.14 acting on the same dsDNA substrate. Representative gel image showing relative guide RNA dependent nicking of the substrate dsDNA catalyzed by the D10A nickase of the BE; TS, target strand. The experiment was done thrice with similar results. For gel images of all replicates, see gel source data.

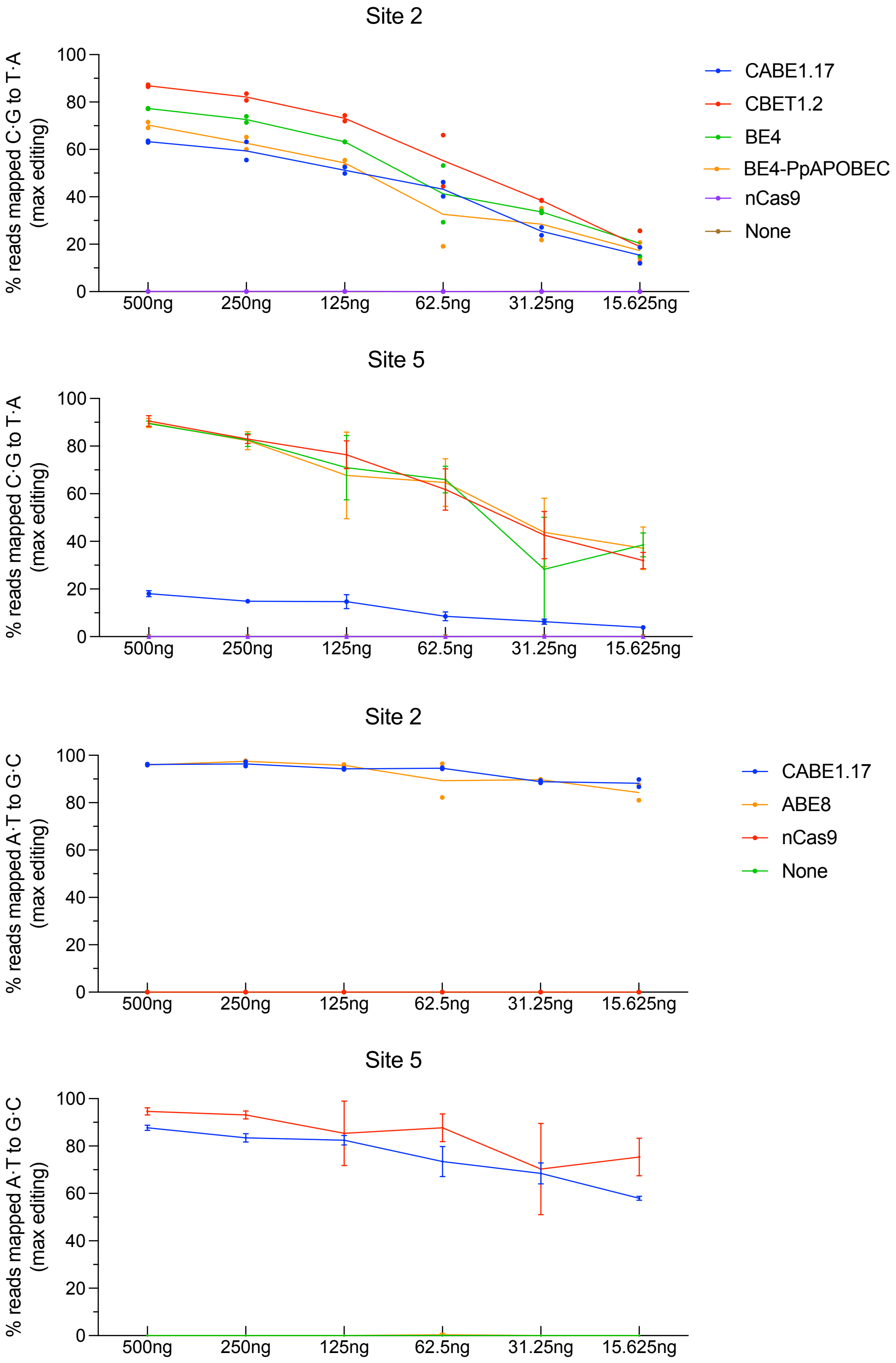

Supplementary Figure 24 | Dose-response curve of percent maximum C·G to T·A or A·T to G·C conversion in transfections of HEK293T with varying concentrations of mRNA. Percent maximum A·T to G·C or C·G to T·A base conversion at targeted genomic loci in HEK293T cells. X-axis represents amount of editor mRNA transfected, with the balance of 500ng total RNA from carrier mRNA. Values and error bars, where applicable, reflect the mean and SD from n = 2 (site 2) or 3 (site 5) independent biological replicates performed on different days. Samples that did not meet the minimum threshold of 5000 sequencing reads mapped to the target site were excluded.

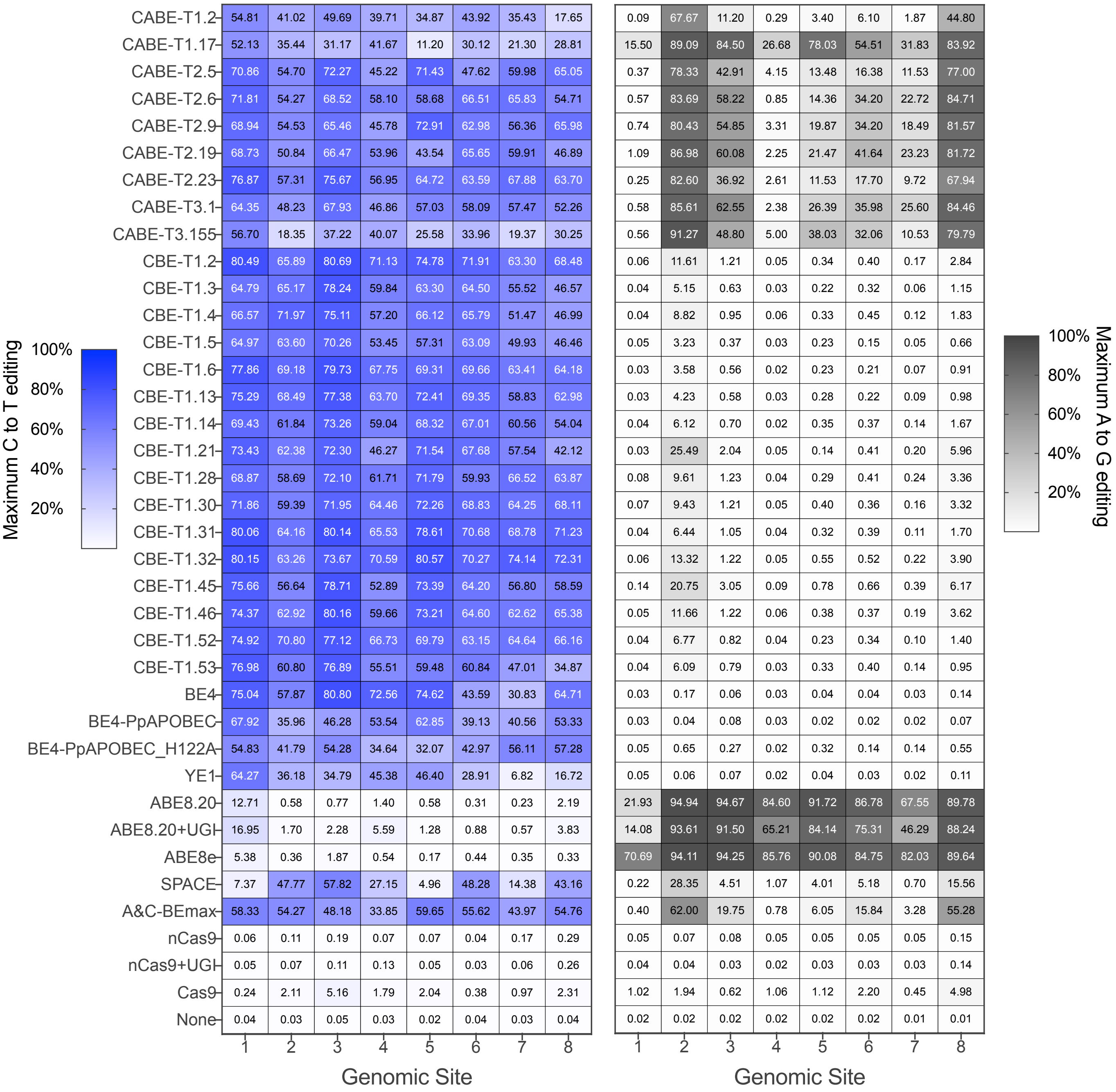

Supplementary Figure 25 | Median C·G to T·A and A·T to G·C conversion of CABE-T and CBE-T constructs at eight genomic sites in HEK293T transfected with mRNA at sub-saturating conditions. Color maps depict the median of percent maximum A·T to G·C or C·G to T·A base conversion at targeted genomic loci in HEK293T cells transfected with mRNA encoding editor (or control) plus synthetic sgRNA at sub-saturating conditions (62.5ng editor mRNA + 437.5ng carrier mRNA + 100ng synthetic guide). Median values were derived from n = 4 independent biological replicates performed on different days. Samples that did not meet the minimum threshold of 5000 sequencing reads mapped to the target site were excluded.

	1	2	3	4	5	6	7	8
CABE-T1.2	1.44	1.06	1.25	0.93	0.86	1.32	1.31	2.21
CABE-T1.17	2.42	1.82	1.71	1.06	1.52	2.06	2.28	1.60
CABE-T2.5	1.66	2.34	1.68	1.27	0.91	1.78	1.22	1.50
CABE-T2.6	1.72	1.65	1.46	1.25	1.15	1.90	1.59	1.65
CABE-T2.9	1.48	2.50	1.44	1.30	0.96	1.85	1.16	1.48
CABE-T2.19	1.86	1.42	1.26	0.93	1.34	1.86	1.20	1.45
CABE-T2.23	1.69	2.10	1.46	1.09	0.80	1.72	1.14	1.49
CABE-T3.1	1.78	2.35	1.34	1.33	1.63	2.05	1.41	1.35
CABE-T3.155	1.59	1.71	1.72	1.12	1.92	1.66	2.35	1.52
CBE-T1.2	1.80	1.92	1.51	1.43	0.62	1.76	1.18	2.92
CBE-T1.3	1.39	1.39	1.03	1.03	0.55	1.18	0.97	2.43
CBE-T1.4	1.05	1.20	0.77	0.90	0.49	1.24	0.89	2.08
CBE-T1.5	1.16	1.41	0.75	0.87	0.50	1.24	0.91	2.09
CBE-T1.6	1.58	1.76	1.28	1.09	0.58	1.58	1.18	2.97
CBE-T1.13	1.58	2.23	1.37	1.09	0.65	1.50	1.24	3.17
CBE-T1.14	1.51	1.63	0.99	1.06	0.58	1.53	1.12	2.51
CBE-T1.21	1.64	2.42	1.81	1.07	0.80	1.68	1.31	3.23
CBE-T1.28	1.55	2.00	1.52	1.45	0.65	1.84	1.20	3.48
CBE-T1.30	1.57	1.81	1.44	1.63	0.70	1.87	1.31	3.39
CBE-T1.31	1.71	1.83	1.57	1.58	0.62	1.96	1.38	3.23
CBE-T1.32	1.81	1.70	1.52	1.51	0.62	1.98	1.38	3.24
CBE-T1.45	1.48	1.69	1.37	1.44	0.63	1.74	1.18	2.35
CBE-T1.46	1.59	1.87	1.44	1.50	0.63	1.79	1.28	3.03
CBE-T1.52	1.68	2.13	1.38	1.37	0.62	1.68	1.29	3.38
CBE-T1.53	1.35	1.51	0.87	1.07	0.51	1.31	0.85	2.53
BE4	1.15	1.95	2.59	0.95	3.12	1.66	2.85	2.92
ABE8.20	5.82	1.55	2.08	1.43	2.03	3.58	2.10	1.79
ABE8.20+ugi	2.00	0.87	1.44	0.84	1.22	2.69	1.55	1.07
ABE8e	6.48	2.01	2.51	2.63	2.16	5.51	2.66	1.61
SPACE	0.55	1.14	4.21	0.64	0.42	1.43	1.06	1.19
A&C-BEmax	1.50	5.43	6.11	0.64	0.80	1.64	1.51	1.39
BE4-ppAPOBEC	1.43	2.16	4.41	1.01	2.16	1.39	1.50	2.57
BE4-ppAPOBEC_H122A	0.69	0.66	1.36	0.47	0.62	0.74	0.40	1.22
YE1	1.46	1.93	2.90	0.64	1.19	1.00	2.14	3.94
nCas9	6.51	9.90	10.62	0.52	2.29	0.37	12.21	17.64
nCas9+UGI	2.88	3.26	4.96	0.31	1.28	0.29	3.63	8.71
Cas9	95.91	96.40	96.76	95.53	97.25	92.29	95.06	95.43
None	0.03	0.03	0.03	0.03	0.04	0.03	0.03	0.02

Genomic Site

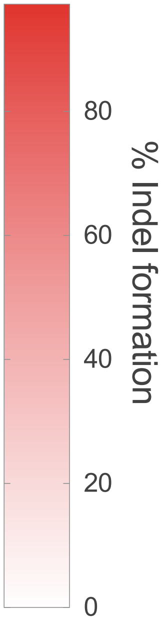

Supplementary Figure 26 | Median indel frequencies of CABE-T and CBE-T editor variants characterized in this study. Median values were determined from n = 3 or 4 biological replicates from transfections of HEK293T cells with mRNA and synthetic guides at saturating conditions.

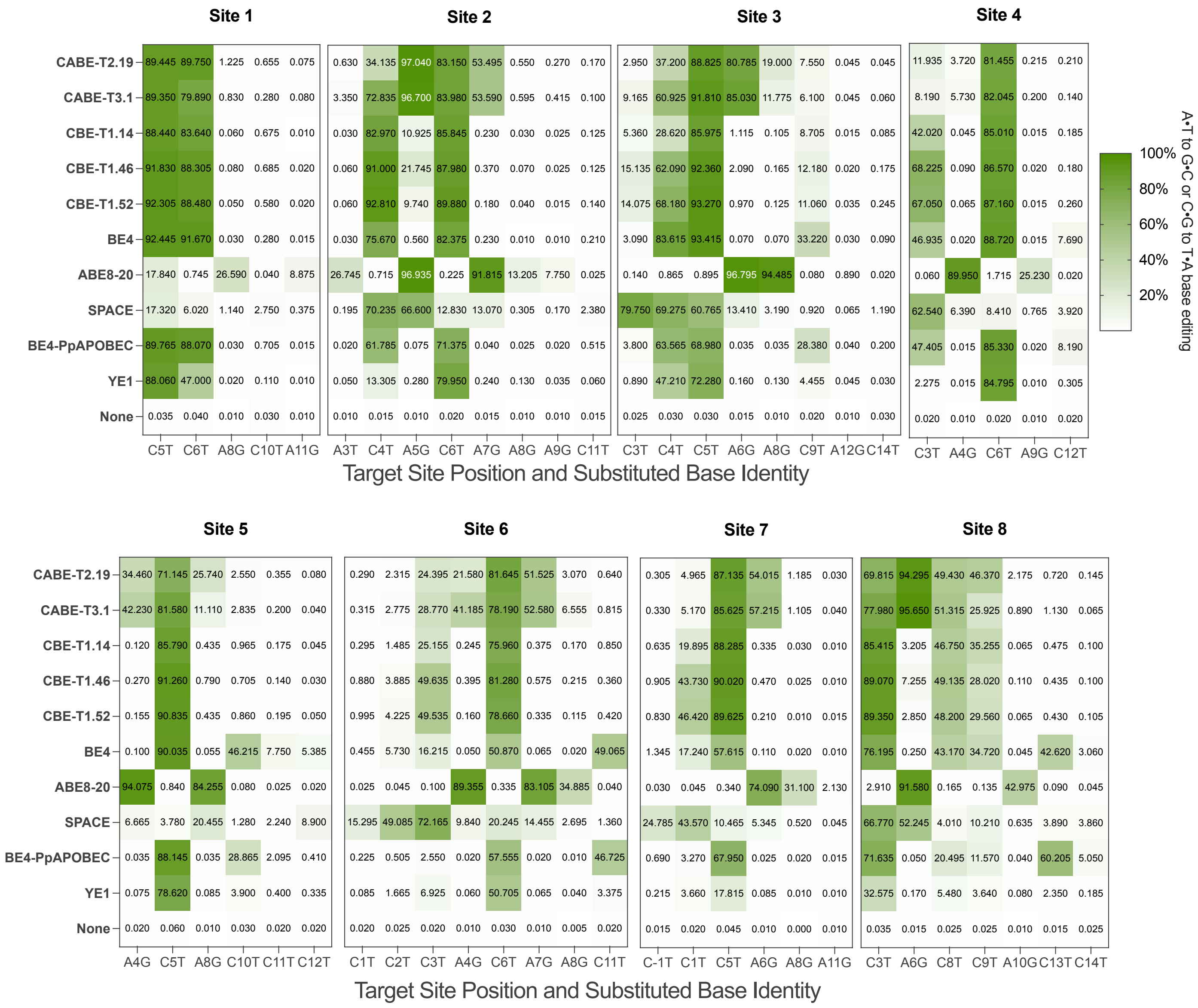

Supplementary Figure 27 | Median C·G to T·A or A·T to G·C conversion of representative CABE-T and CBE-T editor variants across eight genomic sites at each target site position. Percent conversion of C·G to T·A or A·T to G·C at each target site position, as specified on the x-axis, is shown, with the PAM defined as positions 21-23. Median values were determined from n = 4 independent biological replicates via transfections with mRNA and synthetic guides at saturating conditions on different days.

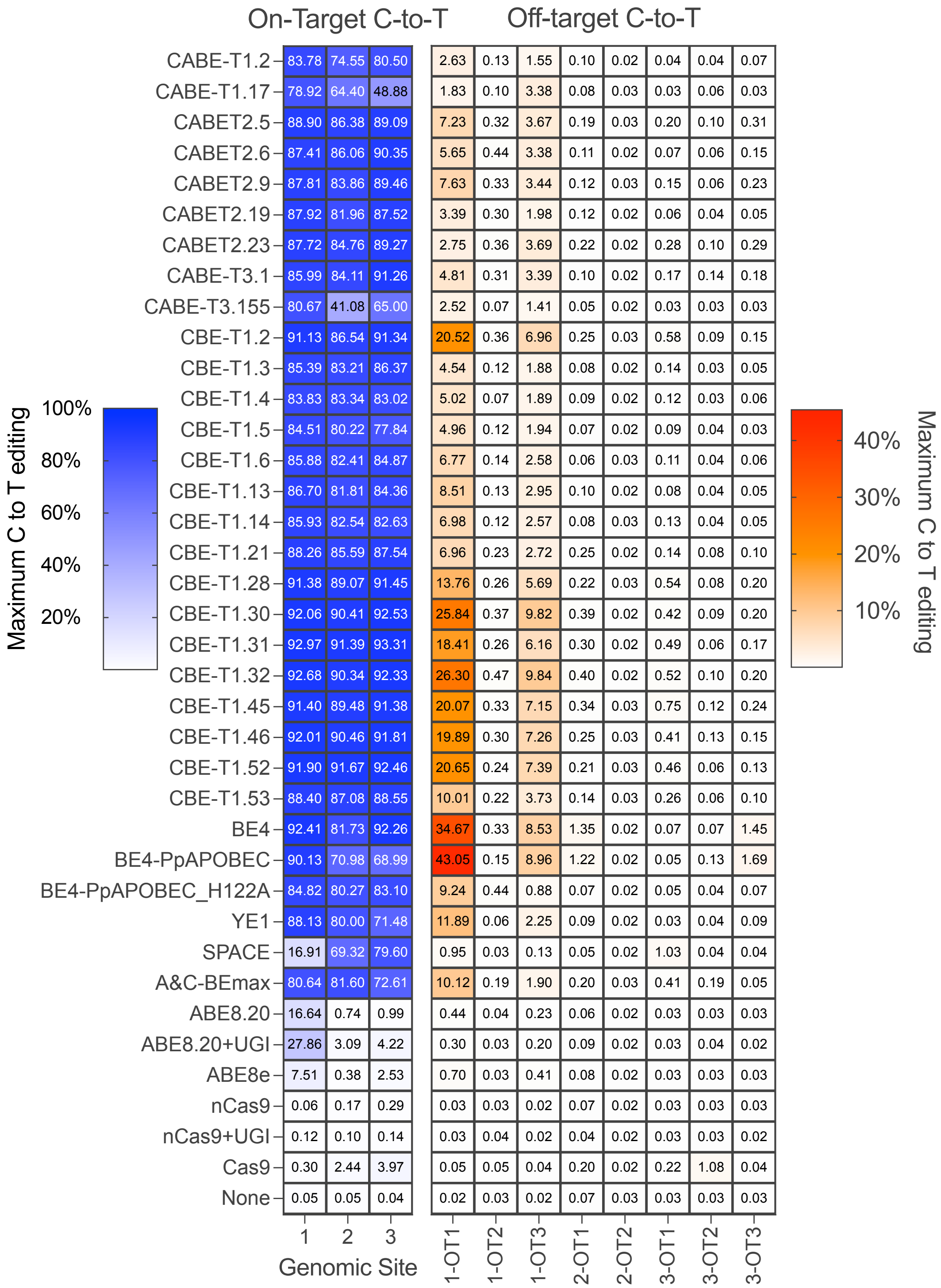

Supplementary Figure 28 | Guide RNA-dependent off-target C·G to T·A conversion of editors evaluated in this study. Color maps depict the median of percent maximum C·G to T·A base conversion at targeted genomic loci (left) and their corresponding off-target sites (right) in HEK293T cells transfected with mRNA encoding editor (or control) plus synthetic sgRNA at saturating conditions (500ng mRNA + 100ng synthetic guide). Median values were derived from n = 3 independent biological replicates performed on different days. Samples that did not meet the minimum threshold of 5000 sequencing reads mapped to the target site were excluded.

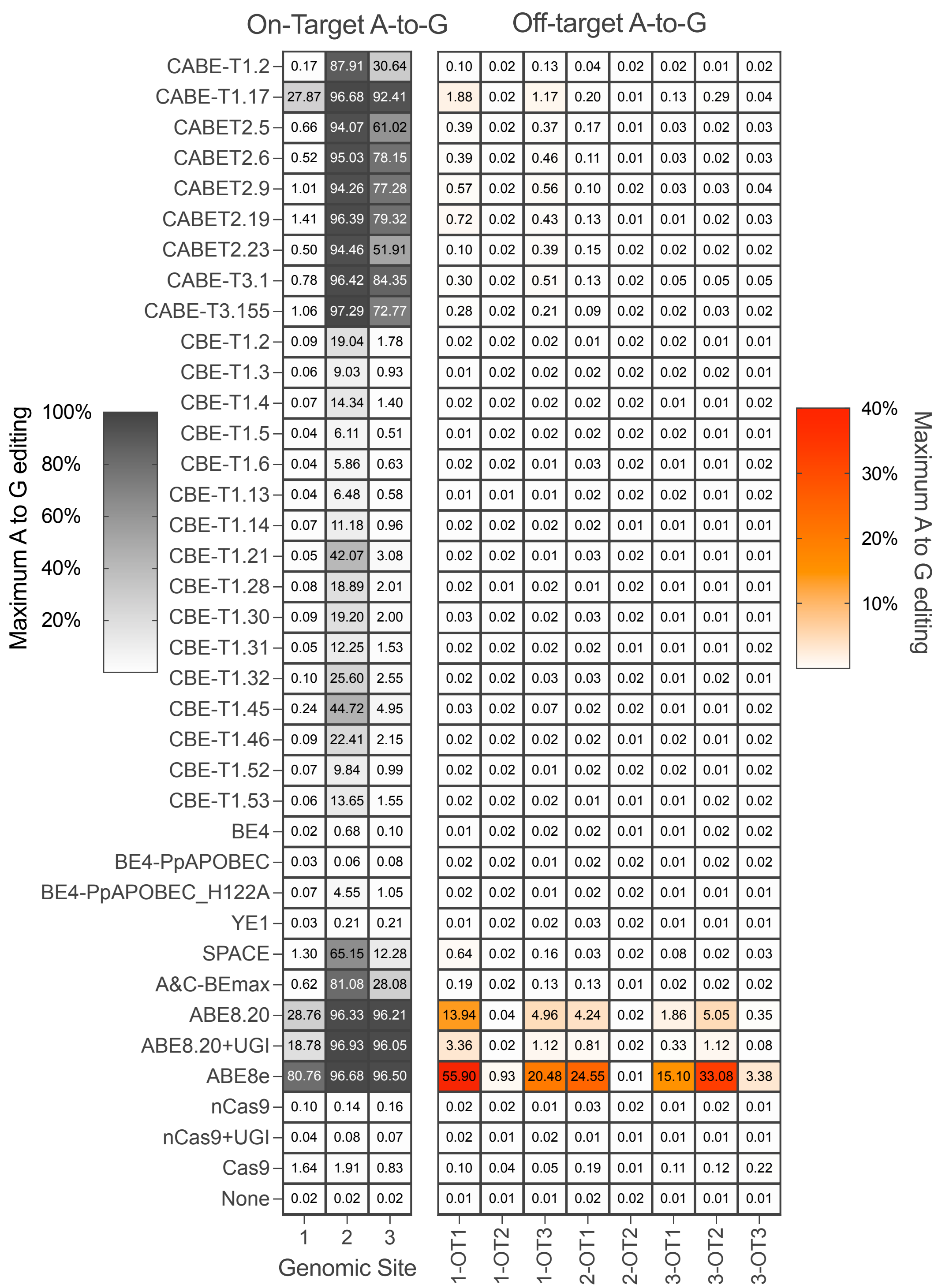

Supplementary Figure 29 | Guide RNA-dependent off-target A·T to G·C conversion of editors evaluated in this study. Color maps depict the median of percent maximum A·T to G·C base conversion at targeted genomic loci (left) and their corresponding off-target sites (right) in HEK293T cells transfected with mRNA encoding editor (or control) plus synthetic sgRNA at saturating conditions (500ng mRNA + 100ng synthetic guide). Median values were derived from n = 3 independent biological replicates performed on different days. Samples that did not meet the minimum threshold of 5000 sequencing reads mapped to the target site were excluded.

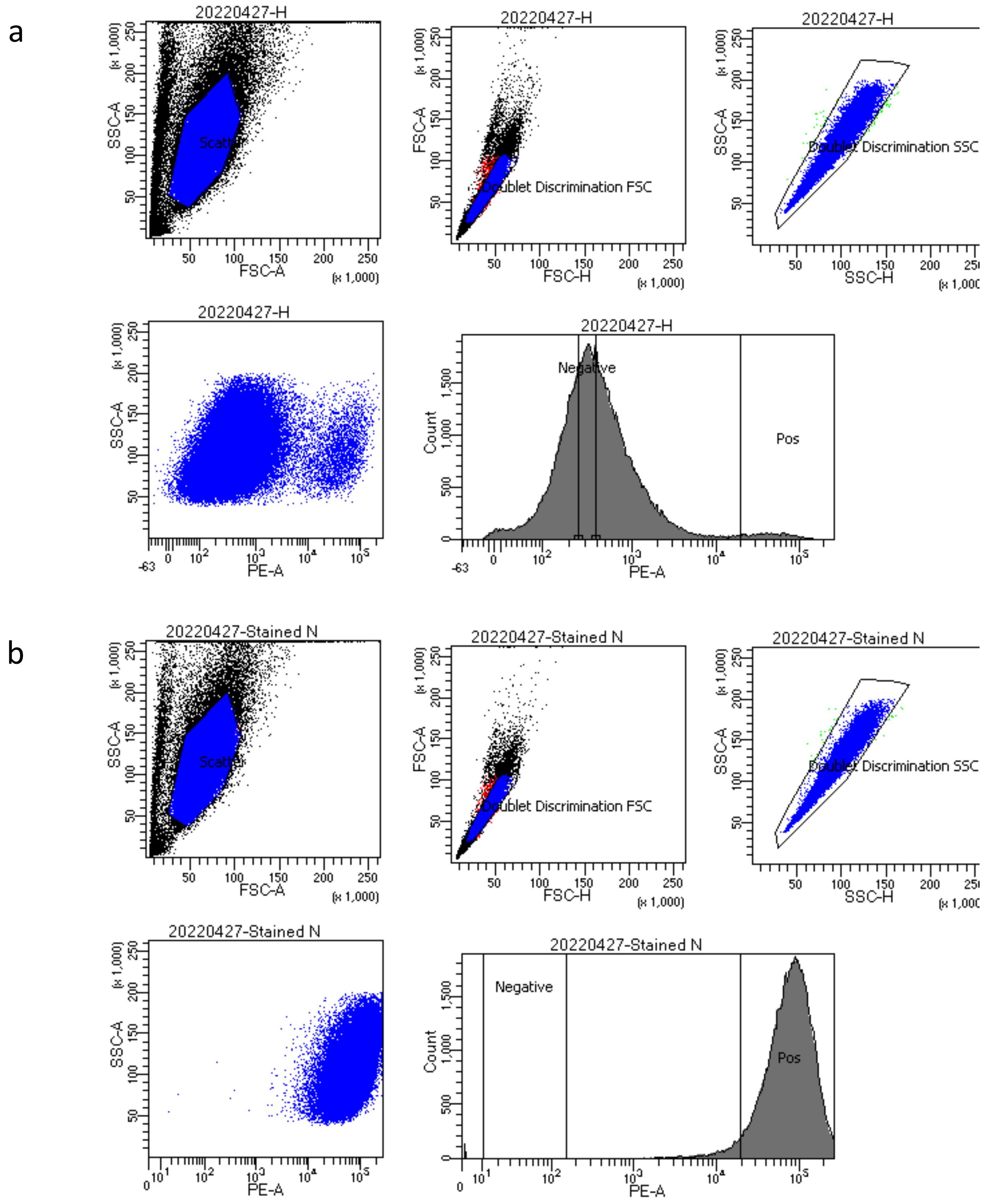

Supplementary Figure 30 | Representative examples of gates used to flow sort live single B2M-positive and B2M-negative cells for whole genome sequencing. a, Representative plots and gates for live, B2M-positive HEK293T cells sorted as single cells for clonal expansion in all treated conditions, and **b,** that of live, B2M-negative HEK293T cells sorted as single cells for clonal expansion in the untreated condition.

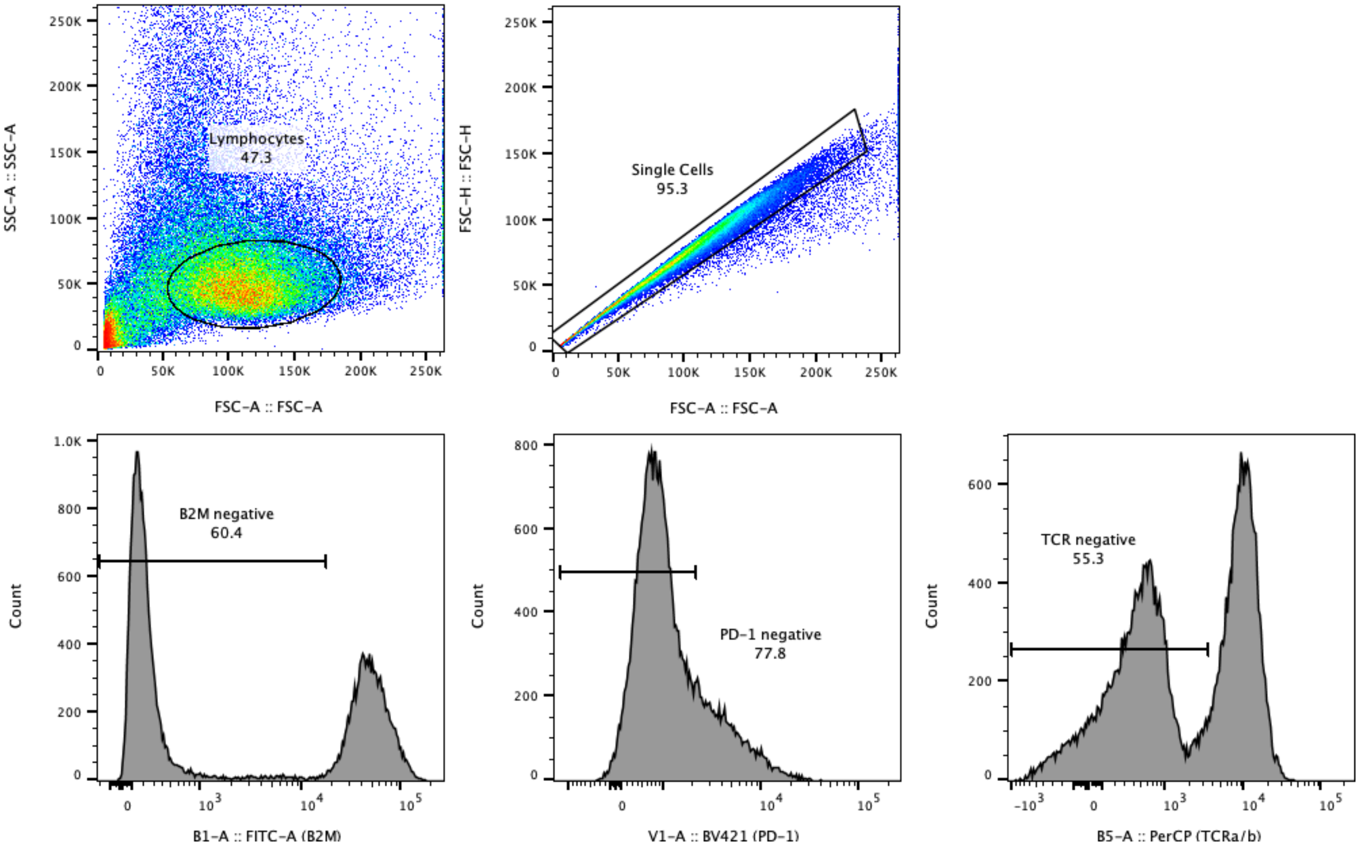

Supplementary Figure 31 | Representative flow gates used for assessment of protein knockdown in T cells. Representative gating strategy for population analysis of live, single, lymphocytes to detect the reduction of cell surface protein expression of B2M, PD-1, and TCRa/b via their respective detection antibodies**.**

Supplementary Table 1 | List of substitutions in TadA*8.20 relative to wild-type *Escherichia coli* TadA (EcTadA).

Residue No.	23	36	48	51	76	82	84	106	108	146	147	152	154	155	156	157
**EcTadA**	W	H	P	R	I	V	L	A	D	S	D	R	Q	E	I	K
**TadA*8.20**	R	L	A	L	Y	S	F	V	N	C	R	P	R	V	F	N

Supplementary Table 2 | Data collection and refinement statistics for crystal structures.

**Data name**	**TadA*8.20-ssDNA1**	**T_{AD}AC-1.17-ssDNA1**	**T_{AD}AC-1.14-holo**	**T_{AD}AC-1.19-holo**
**Ligand in the structure**	ssDNA with d8Az	ssDNA with d8Az	no ligand	no ligand
**PDB ID**	8E2P	8E2Q	8E2R	8E2S
**Data collection**				
Space group	P 31 2 1	P 31 2 1	P 41 21 2	P1
Cell dimensions				
a, b, c (Å)	84.67, 84.67, 214.93	85.93, 85.93, 224.59	89.74, 89.74, 112	59.49, 63.39, 95.17
alpha, beta, gamma (°)	90, 90, 120	90, 90, 120	90, 90, 90	87.89, 82.82, 77.14
Wavelength (Å)	0.9793	0.9763	0.97926	0.97626
Resolution (Å)	43.34-2.72 (2.79-2.72)[a]	62.03-2.34 (2.40-2.34)[a]	56-2.2 (2.28-2.2)[a]	51.88-2.95 (3.03-2.95)[a]
No. of unique reflections	24824 (1776)[a]	41554 (3041)[a]	329638 (24929)[a]	27706 (2043)[a]
Rpim	0.072 (0.744)[a]	0.100 (0.854)[a]	0.027 (0.670)[a]	0.165 (0.393)[a]
I/σ(I)	9.4 (1) [a]	7.7 (1.2)[a]	15.2 (1.2)[a]	2.9 (1.3)[a]
CC1/2	0.995 (0.389)[a]	0.991 (0.470)[a]	0.999 (0.561)[a]	0.918 (0.771)[a]
Completeness (%)	100 (100)[a]	100 (99.9 [a]	99.9 (99.8)[a]	98.0 (97.1)[a]
Redundancy	10.9 (11.6)[a]	9.1 (8.2)[a]	14.2 (14.6)[a]	2.0 (2.0)[a]
**Refinement**				
No. of reflections used	24771 (2428)[a]	41492 (4071)[a]	23237 (2284)[a]	27698 (2663)[a]
Rwork / Rfree[b]	0.1901 / 0.2180	0.1690 / 0.2049	0.2160 / 0.2505	0.2186 / 0.2607
No. of atoms				
Macromolecules	5467	5752	2178	9056
Ligands	88	106	8	8
Water	22	133	38	49
Average B-factors (Å2)				
Macromolecules	66.36	41.73	64.34	32.69
Ligands	57.50	41.63	69.62	27.70
Water	63.77	44.51	63.16	23.57
R.m.s deviations				
Bond lengths (Å)	0.002	0.004	0.004	0.003
Bond angles (°)	0.479	0.59	0.67	0.55
Ramachandran analysis (%)				
Favored	97.33	95.92	95.41	95.86
Allowed	2.67	4.08	4.59	4.14
Outliers	0	0	0	0

[a]Highest resolution shell is shown in parenthesis. [b]R_{free} was calculated with 5% of the data.

Supplementary Table 3 | Sequences of sgRNAs used for transfection. Common names of previously described sgRNAs are given.[7]

Site	sgRNA sequence	PAM	length	Common name
1	GAGUCCGAGCAGAAGAAGAA	GGG	20	EMX1 site 1
2	GAACACAAAGCAUAGACUGC	GGG	20	HEK site 2
3	GGCCCAGACUGAGCACGUGA	TGG	20	HEK site 3
4	GUCAUCUUAGUCAUUACCUG	AGG	20	RNF2 site 1
5	GUGGGACUGAUCCCUUAAUGUG	TGG	22	
6	GCCCAGCAAUUCACUGUGAAG	AGG	21	YY-A16
7	GCCUCAAGUCUGGUUAUUUUAG	AGG	22	YY-A20
8	GGUCGUAGCCAGUCCGAACCC	CGG	21	YY-A28
9	GGCACUGCGGCUGGAGGUGG	GGG	20	Hek site 4
10	GCUCCCAUCACAUCAACCGG	TGG	20	EMX1 site 2
11	GACCAAAACGAGGGACAUUUA	GGG	21	
12	GACCAGGUCAGCAAACAUGUU	TGG	21	
13	GACUCAGCGCCCCUGCCGGGCC	TGG	22	
14	GCACCCAGGGGUUCUGCAGAGC	AGG	22	
15	GCCACAGUGGGAGGGGACAUG	GGG	21	
16	GCCCAGCUCCAGCCUCUGAUG	AGG	21	
17	GCUCCUCUCACCCUUAUGACUC	AGG	22	
18	GGGUACCUGAGUGGGGUGCAUU	TGG	22	
19	GGUCGACCCUUGGUAUCCAUG	GGG	21	
20	GUAACUGAACCCCUGCAAUCAA	TGG	22	
21	GGCCUCCGUAUCACUCUCUGAC	TGG	22	
22	GCUUUCCUUAGCUGUAAAAGAA	AGG	22	
23	GUAUUACUAUUAUUAUCUGAGA	TGG	22	
24	GCCUCAAGUCUGGUUAUUUUAG	GGG	22	
B2M	ACUCACGCUGGAUAGCCUCC	GGG	22	
Q555X	CACCAACAGGGCCACGUCCUCAC	AGG	23	PCSK9 Q555X
E4Sp.	GCUUACCUGUCUGUGGAAGC	GGG	20	PCSK9 E4Splice
CIITA	CACUCACCUUAGCCUGAGCA	GGG	20	CIITA
B2M	ACUCACGCUGGAUAGCCUCC	AGG	20	B2M Ex.1 SD C6
B2M	UUACCCCACUUAACUAUCUU	GGG	20	B2M Ex.2 pmSTOP C6
CD247	CAGGCACAGUUGCCGAUUAC	AGG	20	CD247 Ex.1 pmSTOP C7
CD247	AGUUCCUGCAGAAGAGGGCG	TGG	20	CD247 Ex.6 SA C6
CD3D	AGCCUUACCUUGCGAGAGAA	GGG	20	CD3D Ex.1 SD C8
CD3D	UCUAUCAGGUGAGCGUUGAG	GGG	20	CD3D Ex.4 pmSTOP C6
CD3E	ACUCACCUGAUAAGAGGCAG	AGG	20	CD3E Ex.2 SD C6
CD3E	GUGACACGAGGAGCGGGUGC	TGG	20	CD3E Ex.7 pmSTOP C7
CD3E	UUUGUCCUGCGGAGGAAGGA	GGG	20	CD3E Ex.8 SA C7
CD3G	CUUCAAGGUAAGGGCCUACU	AGG	20	CD3G Ex.1 pmSTOP C4

**CD3G**	ACAUACUUCUGUAAUACACU	TGG	**20**	CD3G Ex.3 SD C6
**TRAC**	UUUCAAAACCUGUCAGUGAU	TGG	**20**	TRAC Ex.3 pmSTOP C4
**TRAC**	UUCGUAUCUGUAAAACCAAG	AGG	**20**	TRAC Ex.3 SA C8
**PD-1**	CACCUACCUAAGAACCAUCC	TGG	**20**	PD-1 Ex.1 SA C7
**PD-1**	GGAGUCUGAGAGAUGGAGAG	AGG	**20**	PD-1 Ex.2 SD C6
**PD-1**	CAGUUCCAAACCCUGGUGGU	TGG	**20**	PD-1 Ex.3 pmSTOP C7
**PD-1**	GACGUUACCUCGUGCGGCCC	GGG	**20**	PD-1 Ex.3 SA C8
**5(*S.a.*)**	GUGGGACUGAUCCCUUAAUGUG	TGGGGT	**22**	Site 5 *S. aureus* PAM
**6(*S.a.*)**	GCCCAGCAAUUCACUGUGAAG	**AGGGAT**	**21**	*Site 6 S. aureus* PAM
**7(*S.a.*)**	GCCUCAAGUCUGGUUAUUUUAG	**AGGAAT**	**22**	Site 7 *S. aureus* PAM
**8(*S.a.*)**	GGUCGUAGCCAGUCCGAACCC	**CGGAGT**	**21**	Site 8 *S. aureus* PAM
**11(*S.a.*)**	GACCAAAACGAGGGACAUUUA	**GGGGAT**	**21**	Site 11 *S. aureus* PAM
**12(*S.a.*)**	GACCAGGUCAGCAAACAUGUU	**TGGAAT**	**21**	Site 12 *S. aureus* PAM

S.a. = S. aureus

S. pyogenes sgRNA scaffold sequence:
GUUUUAGAGCUAGAAAUAGCAAGUUAAAAUAAGGCUAGUCCGUUAUCAACUUGAAAAAGUGGCACCGAGUCGGUGC

S. aureus sgRNA scaffold sequence:
GUUUUAGUACUCUGUAAUGAAAAUUACAGAAUCUACUAAAACAAGGCAAAAUGCCGUGUUUAUCUCGUCAACUUGUUGGCGAGAUUUUUU

Supplementary Table 4 | Primers used to amplify genomic sites for high-throughput, targeted-amplicon sequencing

Primer name	Sequence
site_1_for	ACACTCTTTCCCTACACGACGCTCTTCCGATCTNNNNCAGCTCAGCCTGAGTGTTGA
site_1_rev	TGGAGTTCAGACGTGTGCTCTTCCGATCTCTCGTGGGTTTGTGGTTGC
site_2_for	ACACTCTTTCCCTACACGACGCTCTTCCGATCTNNNNCCAGCCCCATCTGTCAAACT
site_2_rev	TGGAGTTCAGACGTGTGCTCTTCCGATCTTGAATGGATTCCTTGGAAACAATGA
site_3_for	ACACTCTTTCCCTACACGACGCTCTTCCGATCTNNNNATGTGGGCTGCCTAGAAAGG
site_3_rev	TGGAGTTCAGACGTGTGCTCTTCCGATCTCCCAGCCAAACTTGTCAACC
site_4_for	ACACTCTTTCCCTACACGACGCTCTTCCGATCTNNNNACGTCTCATATGCCCCTTGG
site_4_rev	TGGAGTTCAGACGTGTGCTCTTCCGATCTACGTAGGAATTTTGGTGGGACA
site_5_for	ACACTCTTTCCCTACACGACGCTCTTCCGATCTNNNNCTGACACTAAATATGTGGTTTTTTGCT
site_5_rev	TGGAGTTCAGACGTGTGCTCTTCCGATCTGGATTGCGGAAATCCCCAACTTATAGC
site_6_for	ACACTCTTTCCCTACACGACGCTCTTCCGATCTNNNNAGGTGGGGGTGACTCCTTTTTTGGA
site_6_rev	TGGAGTTCAGACGTGTGCTCTTCCGATCTGGGCAGAAGGAAAAATCTATCCTGGAA
site_7_for	ACACTCTTTCCCTACACGACGCTCTTCCGATCTNNNNGCCAAAGTCTGAGGTTTAGTTGACTAA
site_7_rev	TGGAGTTCAGACGTGTGCTCTTCCGATCTATTACAGGTGTGGGCCACCTTGCCC
site_8_for	ACACTCTTTCCCTACACGACGCTCTTCCGATCTNNNNGCTGCTGGAATACCGAGGAC
site_8_rev	TGGAGTTCAGACGTGTGCTCTTCCGATCTGCAACTCTCTTTTCTCCGGGA
site_9_for	ACACTCTTTCCCTACACGACGCTCTTCCGATCTNNNNGAACCCAGGTAGCCAGAGAC
site_9_rev	TGGAGTTCAGACGTGTGCTCTTCCGATCTTCCTTTCAACCCGAACGGAG
site_10_for	ACACTCTTTCCCTACACGACGCTCTTCCGATCTNNNNCCATCAGGCTCTCAGCTCAG
site_10_rev	TGGAGTTCAGACGTGTGCTCTTCCGATCTCTCGTGGGTTTGTGGTTGC
site_11_for	ACACTCTTTCCCTACACGACGCTCTTCCGATCTNNNNGGCTCCCACTCTCTCCCAGTGTCCTCA
site_11_rev	TGGAGTTCAGACGTGTGCTCTTCCGATCTGGATAGAGCAAAAGAAGTAGTGCCTGG
site_12_for	ACACTCTTTCCCTACACGACGCTCTTCCGATCTNNNNTCTGCCTGTGTGAAGCTCCC
site_12_rev	TGGAGTTCAGACGTGTGCTCTTCCGATCTTGAAACTGTCACTGAAACATCTGGT
site_13_for	ACACTCTTTCCCTACACGACGCTCTTCCGATCTNNNNGGGAGTCCTCCCTTCACCCCTGC
site_13_rev	TGGAGTTCAGACGTGTGCTCTTCCGATCTGTTCTCAAGAAAAGGCCACCCCTCAG
site_14_for	ACACTCTTTCCCTACACGACGCTCTTCCGATCTNNNNAGCCTCTTTCCTGCTAGAGC
site_14_rev	TGGAGTTCAGACGTGTGCTCTTCCGATCTTCTCTCCTATGTGCTGGCCT
site_15_for	ACACTCTTTCCCTACACGACGCTCTTCCGATCTNNNNCCATCCTAAGTGAAGCAGCATATTTGA
site_15_rev	TGGAGTTCAGACGTGTGCTCTTCCGATCTAACAATGGCAAGGGCCTGCCCTG
site_16_for	ACACTCTTTCCCTACACGACGCTCTTCCGATCTNNNNCTTGTCTGTCCAAGGAGAATGAGGTC
site_16_rev	TGGAGTTCAGACGTGTGCTCTTCCGATCTGCACAGAACCCGCTGCTAGAGACTCCA
site_17_for	ACACTCTTTCCCTACACGACGCTCTTCCGATCTNNNNAACCAGTCCCTGTCCTGAATCTATCTA
site_17_rev	TGGAGTTCAGACGTGTGCTCTTCCGATCTGGAAGGCAGACTGTATCTGGTCTTTT
site_18_for	ACACTCTTTCCCTACACGACGCTCTTCCGATCTNNNNAAGCTGTCCAGCTGGAAGCCTGGTAA
site_18_rev	TGGAGTTCAGACGTGTGCTCTTCCGATCTCCGCCCAGCACTCGCAGAGCAGA
site_19_for	ACACTCTTTCCCTACACGACGCTCTTCCGATCTNNNNGCCTAAGTTATATGCAAACATCATGCC
site_19_rev	TGGAGTTCAGACGTGTGCTCTTCCGATCTGATGAGAATGCACCATGATTCCAATCA

site_20_for	ACACTCTTTCCCTACACGACGCTCTTCCGATCTNNNNACGAGGTAAGTGTGTGGATTAGTTTCA
site_20_rev	TGGAGTTCAGACGTGTGCTCTTCCGATCTCTACCAAGGAGAGTCATTCCTTTCAGA
site_21_for	ACACTCTTTCCCTACACGACGCTCTTCCGATCTNNNNAGTGGTTACTTTGCCGGGTT
site_21_rev	TGGAGTTCAGACGTGTGCTCTTCCGATCTAAGACAGTCTGGGAAGCGTG
site_22_for	ACACTCTTTCCCTACACGACGCTCTTCCGATCTNNNNACCCTAGCCCTCGGTGCCCTTAGTT
site_22_rev	TGGAGTTCAGACGTGTGCTCTTCCGATCTACTTGGTATTTTAGAAGACTGTTTTCT
site_23_for	ACACTCTTTCCCTACACGACGCTCTTCCGATCTNNNNGTGGGAACATCACCGGAGCCTGG
site_23_rev	TGGAGTTCAGACGTGTGCTCTTCCGATCTTGCATAACCTACACACATCCTCTGATA
site_24_for	ACACTCTTTCCCTACACGACGCTCTTCCGATCTNNNNCCTTTAGGACACATGCTGTCTACCACA
site_24_rev	TGGAGTTCAGACGTGTGCTCTTCCGATCTGTGGTGGAGTGCTCTGTGTTTGTCT
site_B2M_for	ACACTCTTTCCCTACACGACGCTCTTCCGATCTNNNNAGATCCAGCCCTGGACTAGC
site_B2M_rev	TGGAGTTCAGACGTGTGCTCTTCCGATCTGAGATGTCTCGCTCCGTGGC
site1_off1_for	ACACTCTTTCCCTACACGACGCTCTTCCGATCTNNNNAGTAGCCTCTTTCTCAATGTGC
site1_off1_rev	TGGAGTTCAGACGTGTGCTCTTCCGATCTGCTTTCACAAGGATGCAGTCT
site1_off2_for	ACACTCTTTCCCTACACGACGCTCTTCCGATCTNNNNGAGCTAGACTCCGAGGGGA
site1_off2_rev	TGGAGTTCAGACGTGTGCTCTTCCGATCTTCCTCGTCCTGCTCTCACTT
site1_off3_for	ACACTCTTTCCCTACACGACGCTCTTCCGATCTNNNNAGAGGCTGAAGAGGAAGACCA
site1_off3_rev	TGGAGTTCAGACGTGTGCTCTTCCGATCTGGCCCAGCTGTGCATTCTAT
site2_off1_for	ACACTCTTTCCCTACACGACGCTCTTCCGATCTNNNNGTGTGGAGAGTGAGTAAGCCA
site2_off1_rev	TGGAGTTCAGACGTGTGCTCTTCCGATCTACGGTAGGATGATTTCAGGCA
site2_off2_for	ACACTCTTTCCCTACACGACGCTCTTCCGATCTNNNNCACAAAGCAGTGTAGCTCAGG
site2_off2_rev	TGGAGTTCAGACGTGTGCTCTTCCGATCTTTTTTGGTACTCGAGTGTTATTCAG
site3_off1_for	ACACTCTTTCCCTACACGACGCTCTTCCGATCTNNNNTCCCCTGTTGACCTGGAGAA
site3_off1_rev	TGGAGTTCAGACGTGTGCTCTTCCGATCTCACTGTACTTGCCCTGACCA
site3_off2_for	ACACTCTTTCCCTACACGACGCTCTTCCGATCTNNNNTTGGTGTTGACAGGGAGCAA
site3_off2_rev	TGGAGTTCAGACGTGTGCTCTTCCGATCTCTGAGATGTGGGCAGAAGGG
site3_off3_for	ACACTCTTTCCCTACACGACGCTCTTCCGATCTNNNNTGAGAGGGAACAGAAGGGCT
site3_off3_rev	TGGAGTTCAGACGTGTGCTCTTCCGATCTGTCCAAAGGCCCAAGAACCT
PCSK9_A443T_for	ACACTCTTTCCCTACACGACGCTCTTCCGATCTNNNNGCATTGCAGCCATGATGCTG
PCSK9_A443T_rev	TGGAGTTCAGACGTGTGCTCTTCCGATCTAAGGTCACACAGACCTCCCA
PCSK9_Q555X_for	ACACTCTTTCCCTACACGACGCTCTTCCGATCTNNNNGGTGTCTACGCCATTGCC
PCSK9_Q555X_rev	TGGAGTTCAGACGTGTGCTCTTCCGATCTCATGGATCACACTTGTGAGG
PCSK9_E4Splice_for	ACACTCTTTCCCTACACGACGCTCTTCCGATCTNNNNCATACAGAGTGACCACCGGG
PCSK9_E4Splice_rev	TGGAGTTCAGACGTGTGCTCTTCCGATCTGCAGGGAGGGGACAGTGACA
site_CIITA_for	ACACTCTTTCCCTACACGACGCTCTTCCGATCTNNNNATGCAAGTTTGGTCCTGAG
site_CIITA_rev	TGGAGTTCAGACGTGTGCTCTTCCGATCTGATGTGGGTTCCCTGCGCT
B2M Ex.1 SD C6_for	ACACTCTTTCCCTACACGACGCTCTTCCGATCTGCATTCGGGCCGAGATG
B2M Ex.1 SD C6_rev	TGGAGTTCAGACGTGTGCTCTTCCGATCTCTTCCCCGAGATCCAGC
B2M Ex.2 pmSTOP C6_for	ACACTCTTTCCCTACACGACGCTCTTCCGATCTAAGATGAGTATGCCTGCC
B2M Ex.2 pmSTOP C6_rev	TGGAGTTCAGACGTGTGCTCTTCCGATCTTGCAGATTGTTTATATCAGATGG
CD247 Ex.1 pmSTOP C7_for	ACACTCTTTCCCTACACGACGCTCTTCCGATCTAGGACAAGATGAAGTGGAA
CD247 Ex.1 pmSTOP C7_rev	TGGAGTTCAGACGTGTGCTCTTCCGATCTTGCCCATTGATTTGAGGAG
CD247 Ex.6 SA C6_for	ACACTCTTTCCCTACACGACGCTCTTCCGATCTGGAACCTGCTGCTTTCT

CD247 Ex.6 SA C6_rev	TGGAGTTCAGACGTGTGCTCTTCCGATCTGTGGATGGGAAAGGAGG
CD3D Ex.1 SD C8_for	ACACTCTTTCCCTACACGACGCTCTTCCGATCTGGGAGATGGAACATAGCA
CD3D Ex.1 SD C8_rev	TGGAGTTCAGACGTGTGCTCTTCCGATCTTCCCAAAGCTCTGGGAT
CD3D Ex.4 pmSTOP C6_for	ACACTCTTTCCCTACACGACGCTCTTCCGATCTCTGGTTCTTCTAGCTGCC
CD3D Ex.4 pmSTOP C6_rev	TGGAGTTCAGACGTGTGCTCTTCCGATCTAGAGCTCCTGCCTGAC
CD3E Ex.2 SD C6_for	ACACTCTTTCCCTACACGACGCTCTTCCGATCTTGCTGGCCTCCGC
CD3E Ex.2 SD C6_rev	TGGAGTTCAGACGTGTGCTCTTCCGATCTTCTGGTAAATGAGGCTCC
CD3E Ex.7 pmSTOP C7_for	ACACTCTTTCCCTACACGACGCTCTTCCGATCTCTGCTGGTTTACTACTGGA
CD3E Ex.7 pmSTOP C7_rev	TGGAGTTCAGACGTGTGCTCTTCCGATCTGCATAGAAGCTCTAGAGAAGC
CD3E Ex.8 SA C7_for	ACACTCTTTCCCTACACGACGCTCTTCCGATCTTGCTGCAAGATTGGTTCC
CD3E Ex.8 SA C7_rev	TGGAGTTCAGACGTGTGCTCTTCCGATCTAGGCATCTGGCAGACA
CD3G Ex.1 pmSTOP C4_for	ACACTCTTTCCCTACACGACGCTCTTCCGATCTAGACTGACATGGAACAGG
CD3G Ex.1 pmSTOP C4_rev	TGGAGTTCAGACGTGTGCTCTTCCGATCTTTGCCCTTCTTTGAACTCTAAG
CD3G Ex.3 SD C6_for	ACACTCTTTCCCTACACGACGCTCTTCCGATCTGCCAAGGACCCTCGA
CD3G Ex.3 SD C6_rev	TGGAGTTCAGACGTGTGCTCTTCCGATCTTAAGAGAACAGGCGATAATATTAC
TRAC Ex.3 pmSTOP C4_for	ACACTCTTTCCCTACACGACGCTCTTCCGATCTCTGCAGGCCATGCA
TRAC Ex.3 pmSTOP C4_rev	TGGAGTTCAGACGTGTGCTCTTCCGATCTCCCACTCCCAGCTTCA
TRAC Ex.3 SA C8_for	ACACTCTTTCCCTACACGACGCTCTTCCGATCTCTGCAGGCCATGCA
TRAC Ex.3 SA C8_rev	TGGAGTTCAGACGTGTGCTCTTCCGATCTCCCACTCCCAGCTTCA
PD-1 Ex.1 SA C7_for	ACACTCTTTCCCTACACGACGCTCTTCCGATCTTGCTCCAGGCATGCAGA
PD-1 Ex.1 SA C7_rev	TGGAGTTCAGACGTGTGCTCTTCCGATCTCCAGGCCCGCCT
PD-1 Ex.2 SD C6_for	ACACTCTTTCCCTACACGACGCTCTTCCGATCTTGTGAGCAGGTGGCC
PD-1 Ex.2 SD C6_rev	TGGAGTTCAGACGTGTGCTCTTCCGATCTCGGTACCAGTTTAGCACG
PD-1 Ex.3 pmSTOP C7_for	ACACTCTTTCCCTACACGACGCTCTTCCGATCTTGTGCCCTTCCAGAGA
PD-1 Ex.3 pmSTOP C7_rev	TGGAGTTCAGACGTGTGCTCTTCCGATCTCAGGGCAGGCCGAG
PD-1 Ex.3 SA C8_for	ACACTCTTTCCCTACACGACGCTCTTCCGATCTCTGCTGGGCAGCCT
PD-1 Ex.3 SA C8_rev	TGGAGTTCAGACGTGTGCTCTTCCGATCTTTCATCAGGGACTTAGCC

Supplementary Table 5 | The number of residues (1-167) and nucleotides (1-13) visualized in the structures.

Structure	Macromolecule	Chain A	Chain B	Chain C	Chain D	Chain E	Chain F	Chain G	Chain H
TadA*8.20-ssDNA	TadA*8.20	6-156	3-156	4-154	6-156	-	-	-	-
	ssDNA with d8Az	-	-	-	-	1-13	4-13	4-13	3-13
T_{AD}AC-1.17-ssDNA	T_{AD}AC-1.17	4-157	2-161	4-156	3-156	-	-	-	-
	ssDNA with d8Az	-	-	-	-	1-13	1-13	3-13	2-13
T_{AD}AC-1.14-holo	T_{AD}AC-1.14	6-113/115-150	3-108/115-155	NA	NA	NA	NA	NA	NA
T_{AD}AC-1.19-holo	T_{AD}AC-1.19	3-155	1-151	6-155	6-151	6-160	6-151	6-151	4-155

Notes: The first ~5 N-terminal residues and the last 6-21 C-terminal residues are disordered and cannot be seen in the experimental electron density. The first ~3 nucleotides of ssDNA1 are disordered and cannot be seen in the experimental electron density. The residue 114 (chain A) and residues from 109 to 114 (chain B) of the T_{AD}AC1.14 structure are disordered and cannot be seen in the experimental electron density.

Supplementary Sequence 1 | inactivated chloramphenicol resistance gene used in the first round of directed evolution

ggcacgtaagaggttccaactttcaccataatgaaataagatcactaccgggcgtatttttgagttatcgaga
ttttcaggagctaaggaagctaaaATGGAGAAAAAAATCACTGGATATACCACCGTTGATATATCCCAA
TGGCATCGTAAAGAACATTTTGAGGCCTTTCAATCAGTTGCTCAATGTACCTATAACCAGACCGTTCAACTGGA
TATTACGGCCTTTTTAAAGACCGTAAAGAAAAATAAGCACAAGTTTTATCCGGCCTTTATTCACATTCTTGCCC
GCCTGATGAATGCTCATCCGGAGTTCCGTATGGCAATGAAAGACGGTGAGCTGGTGATATGGGATAGTGTTCAC
CCTTGTTACACCGTTTTCCATGAGCAAACTGAAACGTTTTCATCGCTCTGGAGTGAATACCACGACGATTTCCG
GCAGTTTCTACACATATATTCGCAAGATGTGGCGTGTTACGGTGAAAACCTGGCCTATTTCCCTAAAGGGTTTA
TTGAGAATATGTTTTTCGTCTCAGCCAATCCCTGGGTGAGTTTCACCAGTTTTGAT**CC**GAACGTGGCCAATATG
GACAACTTCTTCGCCCCCGTTTTCACTATGGGCAAATATTATACGCAAGGCGACAAGGTGCTGATGCCGCTGGC
CATCCAGGTGCACC**G**CGCCGTATGCGACGGCTTCCATGTCGGCAGAATGCTTAATGAATTACAACAGTACTGCG
ATGAGTGGCAGGGCGGGGCGTAA

lower case = chloramphenicol resistance promoter region
purple = target inactive proline reversion site (L158P)
blue = targeted inactive active site of chloramphenicol resistance gene (H193R)
underline = PAM

Supplementary Sequence 2: inactivated chloramphenicol resistance gene used in the second round of directed evolution

ggcacgtaagaggttccaactttcaccataatgaaataagatcactaccgggcgtatttttgagttatcgaga
ttttcaggagctaaggaagctaaaATGGAGAAAAAAATCACTGGATATACCACCGTTGATATATCCTAA
TGGCATCGTAAAGAACATTTTGAGGCCTTTCAATCAGTTGCTTAATGTACCTATAACCAGACCGTTCAACTGGA
TATTACGGCCTTTTTAAAGACCGTAAAGAAAAATAAGCACAAGTTTTATCCGGCCTTTATTCACATTCTTGCCC
GCCTGATGAATGCTCATCCGGAGTTCCGTATGGCAATGAAAGACGGTGAGCTGGTGATATGGGATAGTGTTCAC
CCTTGTTACACCGTTTTCCATGAGCAAACTGAAACGTTTTCATCGCTCTGGAGTGAATACCACGACGATTTCCG
GCAGTTTCTACACATATATTCGCAAGATGTGGCGTGTTACGGTGAAAACCTGGCCTATTTCCCTAAAGGGTTTA
TTGAGAATATGTTTTTCGTCTCAGCCAATCCCTGGGTGAGTTTCACCAGTTTTGAT**CC**GAACGTGGCCAATATG
GACAACTTCTTCGCCCCCGTTTTCACTATGGGCAAATATTATACGCAAGGCGACAAGGTGCTGATGCCGCTGGC
CATCCAGGTGCACC**G**CGCCGTATGCGACGGCTTCCATGTCGGCAGAATGCTTAATGAATTACAACAGTACTGCG
ATGAGTGGCAGGGCGGGGCGTAA

lower case = chloramphenicol resistance promoter region
red = target STOP codon reversion site (Q15* & Q30*)
purple = target inactive proline reversion site (L158P)
blue = targeted inactive active site of chloramphenicol resistance gene (H193R)
underline = PAM

Supplementary Sequence 3: CABE-T1.2

MSEVEFSHEYWMRHALTLAKRARDERHVPVGAVLVLNNRVIGEGWNRAKGLHDPTAHAEIMALRQGGLVMQNYRLIDATLY
STFEPCVMCAGAMIHSRIGRVVFGVRNAKTGAAGSLMDVLHHPGMNHRVEITEGILADECAALLCRFFRMPRRVFNAQKKA
QSSTDSGGSSGGSSGSETPGTSESATPESSGGSSGGSDKKYSIGLAIGTNSVGWAVITDEYKVPSKKFKVLGNTDRHSIKK
NLIGALLFDSGETAEATRLKRTARRRYTRRKNRICYLQEIFSNEMAKVDDSFFHRLEESFLVEEDKKHERHPIFGNIVDEV
AYHEKYPTIYHLRKKLVDSTDKADLRLIYLALAHMIKFRGHFLIEGDLNPDNSDVDKLFIQLVQTYNQLFEENPINASGVD
AKAILSARLSKSRRLENLIAQLPGEKKNGLFGNLIALSLGLTPNFKSNFDLAEDAKLQLSKDTYDDDLDNLLAQIGDQYAD
LFLAAKNLSDAILLSDILRVNTEITKAPLSASMIKRYDEHHQDLTLLKALVRQQLPEKYKEIFFDQSKNGYAGYIDGGASQ
EEFYKFIKPILEKMDGTEELLVKLNREDLLRKQRTFDNGSIPHQIHLGELHAILRRQEDFYPFLKDNREKIEKILTFRIPY
YVGPLARGNSRFAWMTRKSEETITPWNFEEVVDKGASAQSFIERMTNFDKNLPNEKVLPKHSLLYEYFTVYNELTKVKYVT
EGMRKPAFLSGEQKKAIVDLLFKTNRKVTVKQLKEDYFKKIECFDSVEISGVEDRFNASLGTYHDLLKIIKDKDFLDNEEN
EDILEDIVLTLTLFEDREMIEERLKTYAHLFDDKVMKQLKRRRYTGWGRLSRKLINGIRDKQSGKTILDFLKSDGFANRNF
MQLIHDDSLTFKEDIQKAQVSGQGDSLHEHIANLAGSPAIKKGILQTVKVVDELVKVMGRHKPENIVIEMARENQTTQKGQ
KNSRERMKRIEEGIKELGSQILKEHPVENTQLQNEKLYLYYLQNGRDMYVDQELDINRLSDYDVDHIVPQSFLKDDSIDNK
VLTRSDKNRGKSDNVPSEEVVKKMKNYWRQLLNAKLITQRKFDNLTKAERGGLSELDKAGFIKRQLVETRQITKHVAQILD
SRMNTKYDENDKLIREVKVITLKSKLVSDFRKDFQFYKVREINNYHHAHDAYLNAVVGTALIKKYPKLESEFVYGDYKVYD
VRKMIAKSEQEIGKATAKYFFYSNIMNFFKTEITLANGEIRKRPLIETNGETGEIVWDKGRDFATVRKVLSMPQVNIVKKT
EVQTGGFSKESILPKRNSDKLIARKKDWDPKKYGGFDSPTVAYSVLVVAKVEKGKSKKLKSVKELLGITIMERSSFEKNPI
DFLEAKGYKEVKKDLIIKLPKYSLFELENGRKRMLASAGELQKGNELALPSKYVNFLYLASHYEKLKGSPEDNEQKQLFVE
QHKHYLDEIIEQISEFSKRVILADANLDKVLSAYNKHRDKPIREQAENIIHLFTLTNLGAPAAFKYFDTTIDRKRYTSTKE
VLDATLIHQSITGLYETRIDLSQLGGDSGGSGGSGGSTNLSDIIEKETGKQLVIQESILMLPEEVEEVIGNKPESDILVHT
AYDESTDENVMLLTSDAPEYKPWALVIQDSNGENKIKMLSGGSGGSGGSTNLSDIIEKETGKQLVIQESILMLPEEVEEVI
GNKPESDILVHTAYDESTDENVMLLTSDAPEYKPWALVIQDSNGENKIKMLPKKKRKVEGADKRTADGSEFESPKKKRKV*

Supplementary Sequence 4: CABE-T1.14

MHEVEFSHEYWMRHALTLAKRARDEREVPVGAVLVLNNRVIGEGWNRAKGLHDPTAHAEIMALRQGGLVMQNYRLIDATLY
STFEPCVMCAGAMIHSRIGRVVFGVRNAKTHAAGSLMDVLHHPGMNHRVEITEGILADECAALLCRFFRMPRRVFNAQKKA
QSSTDSGGSSGGSSGSETPGTSESATPESSGGSSGGSDKKYSIGLAIGTNSVGWAVITDEYKVPSKKFKVLGNTDRHSIKK
NLIGALLFDSGETAEATRLKRTARRRYTRRKNRICYLQEIFSNEMAKVDDSFFHRLEESFLVEEDKKHERHPIFGNIVDEV
AYHEKYPTIYHLRKKLVDSTDKADLRLIYLALAHMIKFRGHFLIEGDLNPDNSDVDKLFIQLVQTYNQLFEENPINASGVD
AKAILSARLSKSRRLENLIAQLPGEKKNGLFGNLIALSLGLTPNFKSNFDLAEDAKLQLSKDTYDDDLDNLLAQIGDQYAD
LFLAAKNLSDAILLSDILRVNTEITKAPLSASMIKRYDEHHQDLTLLKALVRQQLPEKYKEIFFDQSKNGYAGYIDGGASQ
EEFYKFIKPILEKMDGTEELLVKLNREDLLRKQRTFDNGSIPHQIHLGELHAILRRQEDFYPFLKDNREKIEKILTFRIPY
YVGPLARGNSRFAWMTRKSEETITPWNFEEVVDKGASAQSFIERMTNFDKNLPNEKVLPKHSLLYEYFTVYNELTKVKYVT
EGMRKPAFLSGEQKKAIVDLLFKTNRKVTVKQLKEDYFKKIECFDSVEISGVEDRFNASLGTYHDLLKIIKDKDFLDNEEN
EDILEDIVLTLTLFEDREMIEERLKTYAHLFDDKVMKQLKRRRYTGWGRLSRKLINGIRDKQSGKTILDFLKSDGFANRNF
MQLIHDDSLTFKEDIQKAQVSGQGDSLHEHIANLAGSPAIKKGILQTVKVVDELVKVMGRHKPENIVIEMARENQTTQKGQ
KNSRERMKRIEEGIKELGSQILKEHPVENTQLQNEKLYLYYLQNGRDMYVDQELDINRLSDYDVDHIVPQSFLKDDSIDNK
VLTRSDKNRGKSDNVPSEEVVKKMKNYWRQLLNAKLITQRKFDNLTKAERGGLSELDKAGFIKRQLVETRQITKHVAQILD
SRMNTKYDENDKLIREVKVITLKSKLVSDFRKDFQFYKVREINNYHHAHDAYLNAVVGTALIKKYPKLESEFVYGDYKVYD
VRKMIAKSEQEIGKATAKYFFYSNIMNFFKTEITLANGEIRKRPLIETNGETGEIVWDKGRDFATVRKVLSMPQVNIVKKT
EVQTGGFSKESILPKRNSDKLIARKKDWDPKKYGGFDSPTVAYSVLVVAKVEKGKSKKLKSVKELLGITIMERSSFEKNPI
DFLEAKGYKEVKKDLIIKLPKYSLFELENGRKRMLASAGELQKGNELALPSKYVNFLYLASHYEKLKGSPEDNEQKQLFVE
QHKHYLDEIIEQISEFSKRVILADANLDKVLSAYNKHRDKPIREQAENIIHLFTLTNLGAPAAFKYFDTTIDRKRYTSTKE
VLDATLIHQSITGLYETRIDLSQLGGDSGGSGGSGGSTNLSDIIEKETGKQLVIQESILMLPEEVEEVIGNKPESDILVHT
AYDESTDENVMLLTSDAPEYKPWALVIQDSNGENKIKMLSGGSGGSGGSTNLSDIIEKETGKQLVIQESILMLPEEVEEVI
GNKPESDILVHTAYDESTDENVMLLTSDAPEYKPWALVIQDSNGENKIKMLPKKKRKVEGADKRTADGSEFESPKKKRKV*

Supplementary Sequence 5: CABE-T1.17

MSEVEFSHEYWMRHALALAKRARDEREVPVGAVLVLNNRVIGEGWNRGIGLHDPTAHAEIMALRQGGLVMQNYRLYDATLY
TTFEPCVMCAGAMIHSRIGRVVFGVRNAKTGAAGSLMDVLHHPGMNHRVEITEGILADECEALLCRFFRMPRRVFNAQKKA
QSSTDSGGSSGGSSGSETPGTSESATPESSGGSSGGSDKKYSIGLAIGTNSVGWAVITDEYKVPSKKFKVLGNTDRHSIKK
NLIGALLFDSGETAEATRLKRTARRRYTRRKNRICYLQEIFSNEMAKVDDSFFHRLEESFLVEEDKKHERHPIFGNIVDEV
AYHEKYPTIYHLRKKLVDSTDKADLRLIYLALAHMIKFRGHFLIEGDLNPDNSDVDKLFIQLVQTYNQLFEENPINASGVD
AKAILSARLSKSRRLENLIAQLPGEKKNGLFGNLIALSLGLTPNFKSNFDLAEDAKLQLSKDTYDDDLDNLLAQIGDQYAD
LFLAAKNLSDAILLSDILRVNTEITKAPLSASMIKRYDEHHQDLTLLKALVRQQLPEKYKEIFFDQSKNGYAGYIDGGASQ
EEFYKFIKPILEKMDGTEELLVKLNREDLLRKQRTFDNGSIPHQIHLGELHAILRRQEDFYPFLKDNREKIEKILTFRIPY
YVGPLARGNSRFAWMTRKSEETITPWNFEEVVDKGASAQSFIERMTNFDKNLPNEKVLPKHSLLYEYFTVYNELTKVKYVT
EGMRKPAFLSGEQKKAIVDLLFKTNRKVTVKQLKEDYFKKIECFDSVEISGVEDRFNASLGTYHDLLKIIKDKDFLDNEEN
EDILEDIVLTLTLFEDREMIEERLKTYAHLFDDKVMKQLKRRRYTGWGRLSRKLINGIRDKQSGKTILDFLKSDGFANRNF
MQLIHDDSLTFKEDIQKAQVSGQGDSLHEHIANLAGSPAIKKGILQTVKVVDELVKVMGRHKPENIVIEMARENQTTQKGQ
KNSRERMKRIEEGIKELGSQILKEHPVENTQLQNEKLYLYYLQNGRDMYVDQELDINRLSDYDVDHIVPQSFLKDDSIDNK
VLTRSDKNRGKSDNVPSEEVVKKMKNYWRQLLNAKLITQRKFDNLTKAERGGLSELDKAGFIKRQLVETRQITKHVAQILD
SRMNTKYDENDKLIREVKVITLKSKLVSDFRKDFQFYKVREINNYHHAHDAYLNAVVGTALIKKYPKLESEFVYGDYKVYD
VRKMIAKSEQEIGKATAKYFFYSNIMNFFKTEITLANGEIRKRPLIETNGETGEIVWDKGRDFATVRKVLSMPQVNIVKKT
EVQTGGFSKESILPKRNSDKLIARKKDWDPKKYGGFDSPTVAYSVLVVAKVEKGKSKKLKSVKELLGITIMERSSFEKNPI
DFLEAKGYKEVKKDLIIKLPKYSLFELENGRKRMLASAGELQKGNELALPSKYVNFLYLASHYEKLKGSPEDNEQKQLFVE
QHKHYLDEIIEQISEFSKRVILADANLDKVLSAYNKHRDKPIREQAENIIHLFTLTNLGAPAAFKYFDTTIDRKRYTSTKE
VLDATLIHQSITGLYETRIDLSQLGGDSGGSGGSGGSTNLSDIIEKETGKQLVIQESILMLPEEVEEVIGNKPESDILVHT
AYDESTDENVMLLTSDAPEYKPWALVIQDSNGENKIKMLSGGSGGSGGSTNLSDIIEKETGKQLVIQESILMLPEEVEEVI
GNKPESDILVHTAYDESTDENVMLLTSDAPEYKPWALVIQDSNGENKIKMLPKKKRKVEGADKRTADGSEFESPKKKRKV*

Supplementary Sequence 6: CABE-T1.19

MSEVEFSHEYWMRHALTLAKRARDERGVPVGAVLVLNNRVIGEGWNRANGLHDPTAHAEIMALRQGGLVMQNYRLYDATLY
STFEPCVMCAGAMIHSRIGRVVFGVRNAKTGAAGSLMDVLHHPGMNHRVEITEGILADECAALLCRFFRMPRRVFNAQKKA
QSSTDSGGSSGGSSGSETPGTSESATPESSGGSSGGSDKKYSIGLAIGTNSVGWAVITDEYKVPSKKFKVLGNTDRHSIKK
NLIGALLFDSGETAEATRLKRTARRRYTRRKNRICYLQEIFSNEMAKVDDSFFHRLEESFLVEEDKKHERHPIFGNIVDEV
AYHEKYPTIYHLRKKLVDSTDKADLRLIYLALAHMIKFRGHFLIEGDLNPDNSDVDKLFIQLVQTYNQLFEENPINASGVD
AKAILSARLSKSRRLENLIAQLPGEKKNGLFGNLIALSLGLTPNFKSNFDLAEDAKLQLSKDTYDDDLDNLLAQIGDQYAD
LFLAAKNLSDAILLSDILRVNTEITKAPLSASMIKRYDEHHQDLTLLKALVRQQLPEKYKEIFFDQSKNGYAGYIDGGASQ
EEFYKFIKPILEKMDGTEELLVKLNREDLLRKQRTFDNGSIPHQIHLGELHAILRRQEDFYPFLKDNREKIEKILTFRIPY
YVGPLARGNSRFAWMTRKSEETITPWNFEEVVDKGASAQSFIERMTNFDKNLPNEKVLPKHSLLYEYFTVYNELTKVKYVT
EGMRKPAFLSGEQKKAIVDLLFKTNRKVTVKQLKEDYFKKIECFDSVEISGVEDRFNASLGTYHDLLKIIKDKDFLDNEEN
EDILEDIVLTLTLFEDREMIEERLKTYAHLFDDKVMKQLKRRRYTGWGRLSRKLINGIRDKQSGKTILDFLKSDGFANRNF
MQLIHDDSLTFKEDIQKAQVSGQGDSLHEHIANLAGSPAIKKGILQTVKVVDELVKVMGRHKPENIVIEMARENQTTQKGQ
KNSRERMKRIEEGIKELGSQILKEHPVENTQLQNEKLYLYYLQNGRDMYVDQELDINRLSDYDVDHIVPQSFLKDDSIDNK
VLTRSDKNRGKSDNVPSEEVVKKMKNYWRQLLNAKLITQRKFDNLTKAERGGLSELDKAGFIKRQLVETRQITKHVAQILD
SRMNTKYDENDKLIREVKVITLKSKLVSDFRKDFQFYKVREINNYHHAHDAYLNAVVGTALIKKYPKLESEFVYGDYKVYD
VRKMIAKSEQEIGKATAKYFFYSNIMNFFKTEITLANGEIRKRPLIETNGETGEIVWDKGRDFATVRKVLSMPQVNIVKKT
EVQTGGFSKESILPKRNSDKLIARKKDWDPKKYGGFDSPTVAYSVLVVAKVEKGKSKKLKSVKELLGITIMERSSFEKNPI
DFLEAKGYKEVKKDLIIKLPKYSLFELENGRKRMLASAGELQKGNELALPSKYVNFLYLASHYEKLKGSPEDNEQKQLFVE
QHKHYLDEIIEQISEFSKRVILADANLDKVLSAYNKHRDKPIREQAENIIHLFTLTNLGAPAAFKYFDTTIDRKRYTSTKE
VLDATLIHQSITGLYETRIDLSQLGGDSGGSGGSGGSTNLSDIIEKETGKQLVIQESILMLPEEVEEVIGNKPESDILVHT
AYDESTDENVMLLTSDAPEYKPWALVIQDSNGENKIKMLSGGSGGSGGSTNLSDIIEKETGKQLVIQESILMLPEEVEEVI
GNKPESDILVHTAYDESTDENVMLLTSDAPEYKPWALVIQDSNGENKIKMLPKKKRKVEGADKRTADGSEFESPKKKRKV*

Supplementary Sequence 7: CABE-T2.5

MSEVEYSHEYWMRHALTLAKRARDERHVPVGAVLVLNNRVIGEGWNRAKGLHDPTAHAEIMALRQGGLVMQNYRLWDATLY
STFEPCVMCAGAMIHSRIGRVVFGVRNAKTGAAGSLMDVLHHPGMNHRVEITEGILADECAALLCRFFRMPRRVFNAQKKA
QSSTDSGGSSGGSSGSETPGTSESATPESSGGSSGGSDKKYSIGLAIGTNSVGWAVITDEYKVPSKKFKVLGNTDRHSIKK
NLIGALLFDSGETAEATRLKRTARRRYTRRKNRICYLQEIFSNEMAKVDDSFFHRLEESFLVEEDKKHERHPIFGNIVDEV
AYHEKYPTIYHLRKKLVDSTDKADLRLIYLALAHMIKFRGHFLIEGDLNPDNSDVDKLFIQLVQTYNQLFEENPINASGVD
AKAILSARLSKSRRLENLIAQLPGEKKNGLFGNLIALSLGLTPNFKSNFDLAEDAKLQLSKDTYDDDLDNLLAQIGDQYAD
LFLAAKNLSDAILLSDILRVNTEITKAPLSASMIKRYDEHHQDLTLLKALVRQQLPEKYKEIFFDQSKNGYAGYIDGGASQ
EEFYKFIKPILEKMDGTEELLVKLNREDLLRKQRTFDNGSIPHQIHLGELHAILRRQEDFYPFLKDNREKIEKILTFRIPY
YVGPLARGNSRFAWMTRKSEETITPWNFEEVVDKGASAQSFIERMTNFDKNLPNEKVLPKHSLLYEYFTVYNELTKVKYVT
EGMRKPAFLSGEQKKAIVDLLFKTNRKVTVKQLKEDYFKKIECFDSVEISGVEDRFNASLGTYHDLLKIIKDKDFLDNEEN
EDILEDIVLTLTLFEDREMIEERLKTYAHLFDDKVMKQLKRRRYTGWGRLSRKLINGIRDKQSGKTILDFLKSDGFANRNF
MQLIHDDSLTFKEDIQKAQVSGQGDSLHEHIANLAGSPAIKKGILQTVKVVDELVKVMGRHKPENIVIEMARENQTTQKGQ
KNSRERMKRIEEGIKELGSQILKEHPVENTQLQNEKLYLYYLQNGRDMYVDQELDINRLSDYDVDHIVPQSFLKDDSIDNK
VLTRSDKNRGKSDNVPSEEVVKKMKNYWRQLLNAKLITQRKFDNLTKAERGGLSELDKAGFIKRQLVETRQITKHVAQILD
SRMNTKYDENDKLIREVKVITLKSKLVSDFRKDFQFYKVREINNYHHAHDAYLNAVVGTALIKKYPKLESEFVYGDYKVYD
VRKMIAKSEQEIGKATAKYFFYSNIMNFFKTEITLANGEIRKRPLIETNGETGEIVWDKGRDFATVRKVLSMPQVNIVKKT
EVQTGGFSKESILPKRNSDKLIARKKDWDPKKYGGFDSPTVAYSVLVVAKVEKGKSKKLKSVKELLGITIMERSSFEKNPI
DFLEAKGYKEVKKDLIIKLPKYSLFELENGRKRMLASAGELQKGNELALPSKYVNFLYLASHYEKLKGSPEDNEQKQLFVE
QHKHYLDEIIEQISEFSKRVILADANLDKVLSAYNKHRDKPIREQAENIIHLFTLTNLGAPAAFKYFDTTIDRKRYTSTKE
VLDATLIHQSITGLYETRIDLSQLGGDSGGSGGSGGSTNLSDIIEKETGKQLVIQESILMLPEEVEEVIGNKPESDILVHT
AYDESTDENVMLLTSDAPEYKPWALVIQDSNGENKIKMLSGGSGGSGGSTNLSDIIEKETGKQLVIQESILMLPEEVEEVI
GNKPESDILVHTAYDESTDENVMLLTSDAPEYKPWALVIQDSNGENKIKMLPKKKRKVEGADKRTADGSEFESPKKKRKV*

Supplementary Sequence 8: CABE-T2.6

MSEVEYSHEYWMRHALTLAKRARDERHVPVGAVLVLNNRVIGEGWNRAKGLHDPTAHAEIMALRQGGLVMQNYRLIDATLY
STFEPCVMCAGAMIHSRIGRVVFGVRNAKTGAAGSLMNVLHHPGMNHRVEITEGILADECAALLCRFFRMPRRVFNAQKKA
QSSTDSGGSSGGSSGSETPGTSESATPESSGGSSGGSDKKYSIGLAIGTNSVGWAVITDEYKVPSKKFKVLGNTDRHSIKK
NLIGALLFDSGETAEATRLKRTARRRYTRRKNRICYLQEIFSNEMAKVDDSFFHRLEESFLVEEDKKHERHPIFGNIVDEV
AYHEKYPTIYHLRKKLVDSTDKADLRLIYLALAHMIKFRGHFLIEGDLNPDNSDVDKLFIQLVQTYNQLFEENPINASGVD
AKAILSARLSKSRRLENLIAQLPGEKKNGLFGNLIALSLGLTPNFKSNFDLAEDAKLQLSKDTYDDDLDNLLAQIGDQYAD
LFLAAKNLSDAILLSDILRVNTEITKAPLSASMIKRYDEHHQDLTLLKALVRQQLPEKYKEIFFDQSKNGYAGYIDGGASQ
EEFYKFIKPILEKMDGTEELLVKLNREDLLRKQRTFDNGSIPHQIHLGELHAILRRQEDFYPFLKDNREKIEKILTFRIPY
YVGPLARGNSRFAWMTRKSEETITPWNFEEVVDKGASAQSFIERMTNFDKNLPNEKVLPKHSLLYEYFTVYNELTKVKYVT
EGMRKPAFLSGEQKKAIVDLLFKTNRKVTVKQLKEDYFKKIECFDSVEISGVEDRFNASLGTYHDLLKIIKDKDFLDNEEN
EDILEDIVLTLTLFEDREMIEERLKTYAHLFDDKVMKQLKRRRYTGWGRLSRKLINGIRDKQSGKTILDFLKSDGFANRNF
MQLIHDDSLTFKEDIQKAQVSGQGDSLHEHIANLAGSPAIKKGILQTVKVVDELVKVMGRHKPENIVIEMARENQTTQKGQ
KNSRERMKRIEEGIKELGSQILKEHPVENTQLQNEKLYLYYLQNGRDMYVDQELDINRLSDYDVDHIVPQSFLKDDSIDNK
VLTRSDKNRGKSDNVPSEEVVKKMKNYWRQLLNAKLITQRKFDNLTKAERGGLSELDKAGFIKRQLVETRQITKHVAQILD
SRMNTKYDENDKLIREVKVITLKSKLVSDFRKDFQFYKVREINNYHHAHDAYLNAVVGTALIKKYPKLESEFVYGDYKVYD
VRKMIAKSEQEIGKATAKYFFYSNIMNFFKTEITLANGEIRKRPLIETNGETGEIVWDKGRDFATVRKVLSMPQVNIVKKT
EVQTGGFSKESILPKRNSDKLIARKKDWDPKKYGGFDSPTVAYSVLVVAKVEKGKSKKLKSVKELLGITIMERSSFEKNPI
DFLEAKGYKEVKKDLIIKLPKYSLFELENGRKRMLASAGELQKGNELALPSKYVNFLYLASHYEKLKGSPEDNEQKQLFVE
QHKHYLDEIIEQISEFSKRVILADANLDKVLSAYNKHRDKPIREQAENIIHLFTLTNLGAPAAFKYFDTTIDRKRYTSTKE
VLDATLIHQSITGLYETRIDLSQLGGDSGGSGGSGGSTNLSDIIEKETGKQLVIQESILMLPEEVEEVIGNKPESDILVHT
AYDESTDENVMLLTSDAPEYKPWALVIQDSNGENKIKMLSGGSGGSGGSTNLSDIIEKETGKQLVIQESILMLPEEVEEVI
GNKPESDILVHTAYDESTDENVMLLTSDAPEYKPWALVIQDSNGENKIKMLPKKKRKVEGADKRTADGSEFESPKKKRKV*

Supplementary Sequence 9: CABE-T2.9

MSEKEFSHEYWMRHALTLAKRARDERHVPVGAVLVLNNRVIGEGWNRAKGLHDPTAHAEIMALRQGGLVMQNYRLWDATLY
STFEPCVMCAGAMIHSRIGRVVFGVRNAKTGAAGSLMDVLTHPGMNHRVEITEGILADECAALLCRFFRMPRRVFNAQKKA
QSSTDSGGSSGGSSGSETPGTSESATPESSGGSSGGSDKKYSIGLAIGTNSVGWAVITDEYKVPSKKFKVLGNTDRHSIKK
NLIGALLFDSGETAEATRLKRTARRRYTRRKNRICYLQEIFSNEMAKVDDSFFHRLEESFLVEEDKKHERHPIFGNIVDEV
AYHEKYPTIYHLRKKLVDSTDKADLRLIYLALAHMIKFRGHFLIEGDLNPDNSDVDKLFIQLVQTYNQLFEENPINASGVD
AKAILSARLSKSRRLENLIAQLPGEKKNGLFGNLIALSLGLTPNFKSNFDLAEDAKLQLSKDTYDDDLDNLLAQIGDQYAD
LFLAAKNLSDAILLSDILRVNTEITKAPLSASMIKRYDEHHQDLTLLKALVRQQLPEKYKEIFFDQSKNGYAGYIDGGASQ
EEFYKFIKPILEKMDGTEELLVKLNREDLLRKQRTFDNGSIPHQIHLGELHAILRRQEDFYPFLKDNREKIEKILTFRIPY
YVGPLARGNSRFAWMTRKSEETITPWNFEEVVDKGASAQSFIERMTNFDKNLPNEKVLPKHSLLYEYFTVYNELTKVKYVT
EGMRKPAFLSGEQKKAIVDLLFKTNRKVTVKQLKEDYFKKIECFDSVEISGVEDRFNASLGTYHDLLKIIKDKDFLDNEEN
EDILEDIVLTLTLFEDREMIEERLKTYAHLFDDKVMKQLKRRRYTGWGRLSRKLINGIRDKQSGKTILDFLKSDGFANRNF
MQLIHDDSLTFKEDIQKAQVSGQGDSLHEHIANLAGSPAIKKGILQTVKVVDELVKVMGRHKPENIVIEMARENQTTQKGQ
KNSRERMKRIEEGIKELGSQILKEHPVENTQLQNEKLYLYYLQNGRDMYVDQELDINRLSDYDVDHIVPQSFLKDDSIDNK
VLTRSDKNRGKSDNVPSEEVVKKMKNYWRQLLNAKLITQRKFDNLTKAERGGLSELDKAGFIKRQLVETRQITKHVAQILD
SRMNTKYDENDKLIREVKVITLKSKLVSDFRKDFQFYKVREINNYHHAHDAYLNAVVGTALIKKYPKLESEFVYGDYKVYD
VRKMIAKSEQEIGKATAKYFFYSNIMNFFKTEITLANGEIRKRPLIETNGETGEIVWDKGRDFATVRKVLSMPQVNIVKKT
EVQTGGFSKESILPKRNSDKLIARKKDWDPKKYGGFDSPTVAYSVLVVAKVEKGKSKKLKSVKELLGITIMERSSFEKNPI
DFLEAKGYKEVKKDLIIKLPKYSLFELENGRKRMLASAGELQKGNELALPSKYVNFLYLASHYEKLKGSPEDNEQKQLFVE
QHKHYLDEIIEQISEFSKRVILADANLDKVLSAYNKHRDKPIREQAENIIHLFTLTNLGAPAAFKYFDTTIDRKRYTSTKE
VLDATLIHQSITGLYETRIDLSQLGGDSGGSGGSGGSTNLSDIIEKETGKQLVIQESILMLPEEVEEVIGNKPESDILVHT
AYDESTDENVMLLTSDAPEYKPWALVIQDSNGENKIKMLSGGSGGSGGSTNLSDIIEKETGKQLVIQESILMLPEEVEEVI
GNKPESDILVHTAYDESTDENVMLLTSDAPEYKPWALVIQDSNGENKIKMLPKKKRKVEGADKRTADGSEFESPKKKRKV*

Supplementary Sequence 10: CABE-T2.19

MSEVEFSHEYWMRHALTLAKRARDERHVPVGAVLVLNNRVIGEGWNRAKGLHDPTAHAEIMALRQGGLVMQNYRLIDATLY
STFEPCVMCAGAMIHSRIGRVVFGVRNAKHGAAGSLMDVLGHPGMNHRVEITEGILADECAALLCRFFRMPRRVFNAQKKC
QSSTDSGGSSGGSSGSETPGTSESATPESSGGSSGGSDKKYSIGLAIGTNSVGWAVITDEYKVPSKKFKVLGNTDRHSIKK
NLIGALLFDSGETAEATRLKRTARRRYTRRKNRICYLQEIFSNEMAKVDDSFFHRLEESFLVEEDKKHERHPIFGNIVDEV
AYHEKYPTIYHLRKKLVDSTDKADLRLIYLALAHMIKFRGHFLIEGDLNPDNSDVDKLFIQLVQTYNQLFEENPINASGVD
AKAILSARLSKSRRLENLIAQLPGEKKNGLFGNLIALSLGLTPNFKSNFDLAEDAKLQLSKDTYDDDLDNLLAQIGDQYAD
LFLAAKNLSDAILLSDILRVNTEITKAPLSASMIKRYDEHHQDLTLLKALVRQQLPEKYKEIFFDQSKNGYAGYIDGGASQ
EEFYKFIKPILEKMDGTEELLVKLNREDLLRKQRTFDNGSIPHQIHLGELHAILRRQEDFYPFLKDNREKIEKILTFRIPY
YVGPLARGNSRFAWMTRKSEETITPWNFEEVVDKGASAQSFIERMTNFDKNLPNEKVLPKHSLLYEYFTVYNELTKVKYVT
EGMRKPAFLSGEQKKAIVDLLFKTNRKVTVKQLKEDYFKKIECFDSVEISGVEDRFNASLGTYHDLLKIIKDKDFLDNEEN
EDILEDIVLTLTLFEDREMIEERLKTYAHLFDDKVMKQLKRRRYTGWGRLSRKLINGIRDKQSGKTILDFLKSDGFANRNF
MQLIHDDSLTFKEDIQKAQVSGQGDSLHEHIANLAGSPAIKKGILQTVKVVDELVKVMGRHKPENIVIEMARENQTTQKGQ
KNSRERMKRIEEGIKELGSQILKEHPVENTQLQNEKLYLYYLQNGRDMYVDQELDINRLSDYDVDHIVPQSFLKDDSIDNK
VLTRSDKNRGKSDNVPSEEVVKKMKNYWRQLLNAKLITQRKFDNLTKAERGGLSELDKAGFIKRQLVETRQITKHVAQILD
SRMNTKYDENDKLIREVKVITLKSKLVSDFRKDFQFYKVREINNYHHAHDAYLNAVVGTALIKKYPKLESEFVYGDYKVYD
VRKMIAKSEQEIGKATAKYFFYSNIMNFFKTEITLANGEIRKRPLIETNGETGEIVWDKGRDFATVRKVLSMPQVNIVKKT
EVQTGGFSKESILPKRNSDKLIARKKDWDPKKYGGFDSPTVAYSVLVVAKVEKGKSKKLKSVKELLGITIMERSSFEKNPI
DFLEAKGYKEVKKDLIIKLPKYSLFELENGRKRMLASAGELQKGNELALPSKYVNFLYLASHYEKLKGSPEDNEQKQLFVE
QHKHYLDEIIEQISEFSKRVILADANLDKVLSAYNKHRDKPIREQAENIIHLFTLTNLGAPAAFKYFDTTIDRKRYTSTKE
VLDATLIHQSITGLYETRIDLSQLGGDSGGSGGSGGSTNLSDIIEKETGKQLVIQESILMLPEEVEEVIGNKPESDILVHT
AYDESTDENVMLLTSDAPEYKPWALVIQDSNGENKIKMLSGGSGGSGGSTNLSDIIEKETGKQLVIQESILMLPEEVEEVI
GNKPESDILVHTAYDESTDENVMLLTSDAPEYKPWALVIQDSNGENKIKMLPKKKRKVEGADKRTADGSEFESPKKKRKV*

Supplementary Sequence 11: CABE-T2.23

MSESEFSHEYWMRHALTLAKRARDERHVPVGAVLVLNNRVIGEGWNRAKGLHDPTAHAEIMALRQGGLVMQNYRLYDATLY
STFEPCVMCAGAMIHSRIGRVVFGVRNAKTGAAGSLMDVLHHPGMNHRVEITEGILADECAELLCRFFRMPRRVFNASKKA
QSSTDSGGSSGGSSGSETPGTSESATPESSGGSSGGSDKKYSIGLAIGTNSVGWAVITDEYKVPSKKFKVLGNTDRHSIKK
NLIGALLFDSGETAEATRLKRTARRRYTRRKNRICYLQEIFSNEMAKVDDSFFHRLEESFLVEEDKKHERHPIFGNIVDEV
AYHEKYPTIYHLRKKLVDSTDKADLRLIYLALAHMIKFRGHFLIEGDLNPDNSDVDKLFIQLVQTYNQLFEENPINASGVD
AKAILSARLSKSRRLENLIAQLPGEKKNGLFGNLIALSLGLTPNFKSNFDLAEDAKLQLSKDTYDDDLDNLLAQIGDQYAD
LFLAAKNLSDAILLSDILRVNTEITKAPLSASMIKRYDEHHQDLTLLKALVRQQLPEKYKEIFFDQSKNGYAGYIDGGASQ
EEFYKFIKPILEKMDGTEELLVKLNREDLLRKQRTFDNGSIPHQIHLGELHAILRRQEDFYPFLKDNREKIEKILTFRIPY
YVGPLARGNSRFAWMTRKSEETITPWNFEEVVDKGASAQSFIERMTNFDKNLPNEKVLPKHSLLYEYFTVYNELTKVKYVT
EGMRKPAFLSGEQKKAIVDLLFKTNRKVTVKQLKEDYFKKIECFDSVEISGVEDRFNASLGTYHDLLKIIKDKDFLDNEEN
EDILEDIVLTLTLFEDREMIEERLKTYAHLFDDKVMKQLKRRRYTGWGRLSRKLINGIRDKQSGKTILDFLKSDGFANRNF
MQLIHDDSLTFKEDIQKAQVSGQGDSLHEHIANLAGSPAIKKGILQTVKVVDELVKVMGRHKPENIVIEMARENQTTQKGQ
KNSRERMKRIEEGIKELGSQILKEHPVENTQLQNEKLYLYYLQNGRDMYVDQELDINRLSDYDVDHIVPQSFLKDDSIDNK
VLTRSDKNRGKSDNVPSEEVVKKMKNYWRQLLNAKLITQRKFDNLTKAERGGLSELDKAGFIKRQLVETRQITKHVAQILD
SRMNTKYDENDKLIREVKVITLKSKLVSDFRKDFQFYKVREINNYHHAHDAYLNAVVGTALIKKYPKLESEFVYGDYKVYD
VRKMIAKSEQEIGKATAKYFFYSNIMNFFKTEITLANGEIRKRPLIETNGETGEIVWDKGRDFATVRKVLSMPQVNIVKKT
EVQTGGFSKESILPKRNSDKLIARKKDWDPKKYGGFDSPTVAYSVLVVAKVEKGKSKKLKSVKELLGITIMERSSFEKNPI
DFLEAKGYKEVKKDLIIKLPKYSLFELENGRKRMLASAGELQKGNELALPSKYVNFLYLASHYEKLKGSPEDNEQKQLFVE
QHKHYLDEIIEQISEFSKRVILADANLDKVLSAYNKHRDKPIREQAENIIHLFTLTNLGAPAAFKYFDTTIDRKRYTSTKE
VLDATLIHQSITGLYETRIDLSQLGGDSGGSGGSGGSTNLSDIIEKETGKQLVIQESILMLPEEVEEVIGNKPESDILVHT
AYDESTDENVMLLTSDAPEYKPWALVIQDSNGENKIKMLSGGSGGSGGSTNLSDIIEKETGKQLVIQESILMLPEEVEEVI
GNKPESDILVHTAYDESTDENVMLLTSDAPEYKPWALVIQDSNGENKIKMLPKKKRKVEGADKRTADGSEFESPKKKRKV*

Supplementary Sequence 12: CABE-T3.1

MSEVEFSHEYWMRHALTLAKRARDERSVPVGAVLVLNNRVIGEGWNRAKGLHDPTAHAEIMALRQGGLVMQNYRLYDATLY
TTFEPCVMCAGAMIHSRIGRVVFGVRNAKTGAAGSLMDVLHHPGMNHRVEITEGILADECAALLCRFFRMPRRVFNAQKKA
QSSTDSGGSSGGSSGSETPGTSESATPESSGGSSGGSDKKYSIGLAIGTNSVGWAVITDEYKVPSKKFKVLGNTDRHSIKK
NLIGALLFDSGETAEATRLKRTARRRYTRRKNRICYLQEIFSNEMAKVDDSFFHRLEESFLVEEDKKHERHPIFGNIVDEV
AYHEKYPTIYHLRKKLVDSTDKADLRLIYLALAHMIKFRGHFLIEGDLNPDNSDVDKLFIQLVQTYNQLFEENPINASGVD
AKAILSARLSKSRRLENLIAQLPGEKKNGLFGNLIALSLGLTPNFKSNFDLAEDAKLQLSKDTYDDDLDNLLAQIGDQYAD
LFLAAKNLSDAILLSDILRVNTEITKAPLSASMIKRYDEHHQDLTLLKALVRQQLPEKYKEIFFDQSKNGYAGYIDGGASQ
EEFYKFIKPILEKMDGTEELLVKLNREDLLRKQRTFDNGSIPHQIHLGELHAILRRQEDFYPFLKDNREKIEKILTFRIPY
YVGPLARGNSRFAWMTRKSEETITPWNFEEVVDKGASAQSFIERMTNFDKNLPNEKVLPKHSLLYEYFTVYNELTKVKYVT
EGMRKPAFLSGEQKKAIVDLLFKTNRKVTVKQLKEDYFKKIECFDSVEISGVEDRFNASLGTYHDLLKIIKDKDFLDNEEN
EDILEDIVLTLTLFEDREMIEERLKTYAHLFDDKVMKQLKRRRYTGWGRLSRKLINGIRDKQSGKTILDFLKSDGFANRNF
MQLIHDDSLTFKEDIQKAQVSGQGDSLHEHIANLAGSPAIKKGILQTVKVVDELVKVMGRHKPENIVIEMARENQTTQKGQ
KNSRERMKRIEEGIKELGSQILKEHPVENTQLQNEKLYLYYLQNGRDMYVDQELDINRLSDYDVDHIVPQSFLKDDSIDNK
VLTRSDKNRGKSDNVPSEEVVKKMKNYWRQLLNAKLITQRKFDNLTKAERGGLSELDKAGFIKRQLVETRQITKHVAQILD
SRMNTKYDENDKLIREVKVITLKSKLVSDFRKDFQFYKVREINNYHHAHDAYLNAVVGTALIKKYPKLESEFVYGDYKVYD
VRKMIAKSEQEIGKATAKYFFYSNIMNFFKTEITLANGEIRKRPLIETNGETGEIVWDKGRDFATVRKVLSMPQVNIVKKT
EVQTGGFSKESILPKRNSDKLIARKKDWDPKKYGGFDSPTVAYSVLVVAKVEKGKSKKLKSVKELLGITIMERSSFEKNPI
DFLEAKGYKEVKKDLIIKLPKYSLFELENGRKRMLASAGELQKGNELALPSKYVNFLYLASHYEKLKGSPEDNEQKQLFVE
QHKHYLDEIIEQISEFSKRVILADANLDKVLSAYNKHRDKPIREQAENIIHLFTLTNLGAPAAFKYFDTTIDRKRYTSTKE
VLDATLIHQSITGLYETRIDLSQLGGDSGGSGGSGGSTNLSDIIEKETGKQLVIQESILMLPEEVEEVIGNKPESDILVHT
AYDESTDENVMLLTSDAPEYKPWALVIQDSNGENKIKMLSGGSGGSGGSTNLSDIIEKETGKQLVIQESILMLPEEVEEVI
GNKPESDILVHTAYDESTDENVMLLTSDAPEYKPWALVIQDSNGENKIKMLPKKKRKVEGADKRTADGSEFESPKKKRKV*

Supplementary Sequence 13: CABE-T3.153

MSEVEFSHEYWMRHALTLAKRARDERHVPVGAVLVLNNRVIGEGWNRAKGLHDPTAHAEIMALRQGGLVMQNYRLYDATLY
TTFEPCVMCAGAMIHSRIGRVVFGVRNAKTGAAMSLMDVLHHPGMNHRVEITEGILADECEALLCRFFRMPRRVFNAQKKA
QSSTDSGGSSGGSSGSETPGTSESATPESSGGSSGGSDKKYSIGLAIGTNSVGWAVITDEYKVPSKKFKVLGNTDRHSIKK
NLIGALLFDSGETAEATRLKRTARRRYTRRKNRICYLQEIFSNEMAKVDDSFFHRLEESFLVEEDKKHERHPIFGNIVDEV
AYHEKYPTIYHLRKKLVDSTDKADLRLIYLALAHMIKFRGHFLIEGDLNPDNSDVDKLFIQLVQTYNQLFEENPINASGVD
AKAILSARLSKSRRLENLIAQLPGEKKNGLFGNLIALSLGLTPNFKSNFDLAEDAKLQLSKDTYDDDLDNLLAQIGDQYAD
LFLAAKNLSDAILLSDILRVNTEITKAPLSASMIKRYDEHHQDLTLLKALVRQQLPEKYKEIFFDQSKNGYAGYIDGGASQ
EEFYKFIKPILEKMDGTEELLVKLNREDLLRKQRTFDNGSIPHQIHLGELHAILRRQEDFYPFLKDNREKIEKILTFRIPY
YVGPLARGNSRFAWMTRKSEETITPWNFEEVVDKGASAQSFIERMTNFDKNLPNEKVLPKHSLLYEYFTVYNELTKVKYVT
EGMRKPAFLSGEQKKAIVDLLFKTNRKVTVKQLKEDYFKKIECFDSVEISGVEDRFNASLGTYHDLLKIIKDKDFLDNEEN
EDILEDIVLTLTLFEDREMIEERLKTYAHLFDDKVMKQLKRRRYTGWGRLSRKLINGIRDKQSGKTILDFLKSDGFANRNF
MQLIHDDSLTFKEDIQKAQVSGQGDSLHEHIANLAGSPAIKKGILQTVKVVDELVKVMGRHKPENIVIEMARENQTTQKGQ
KNSRERMKRIEEGIKELGSQILKEHPVENTQLQNEKLYLYYLQNGRDMYVDQELDINRLSDYDVDHIVPQSFLKDDSIDNK
VLTRSDKNRGKSDNVPSEEVVKKMKNYWRQLLNAKLITQRKFDNLTKAERGGLSELDKAGFIKRQLVETRQITKHVAQILD
SRMNTKYDENDKLIREVKVITLKSKLVSDFRKDFQFYKVREINNYHHAHDAYLNAVVGTALIKKYPKLESEFVYGDYKVYD
VRKMIAKSEQEIGKATAKYFFYSNIMNFFKTEITLANGEIRKRPLIETNGETGEIVWDKGRDFATVRKVLSMPQVNIVKKT
EVQTGGFSKESILPKRNSDKLIARKKDWDPKKYGGFDSPTVAYSVLVVAKVEKGKSKKLKSVKELLGITIMERSSFEKNPI
DFLEAKGYKEVKKDLIIKLPKYSLFELENGRKRMLASAGELQKGNELALPSKYVNFLYLASHYEKLKGSPEDNEQKQLFVE
QHKHYLDEIIEQISEFSKRVILADANLDKVLSAYNKHRDKPIREQAENIIHLFTLTNLGAPAAFKYFDTTIDRKRYTSTKE
VLDATLIHQSITGLYETRIDLSQLGGDSGGSGGSGGSTNLSDIIEKETGKQLVIQESILMLPEEVEEVIGNKPESDILVHT
AYDESTDENVMLLTSDAPEYKPWALVIQDSNGENKIKMLSGGSGGSGGSTNLSDIIEKETGKQLVIQESILMLPEEVEEVI
GNKPESDILVHTAYDESTDENVMLLTSDAPEYKPWALVIQDSNGENKIKMLPKKKRKVEGADKRTADGSEFESPKKKRKV*

Supplementary Sequence 14: CABE-T3.154

MSEVEFSHEYWMRHALTLAKRARDERHVPVGAVLVLNNRVIGEGWNRAKGLHDPTAHAEIMALRQGGLVMQNYRLYDATLY
TTFEPCVMCAGAMIHSRIGRVVFGVCNAKTHAAMSLMDVLHHPGMNHRVEITEGILADECEALLCRFFRMPRRVFNAQKKA
QSSTDSGGSSGGSSGSETPGTSESATPESSGGSSGGSDKKYSIGLAIGTNSVGWAVITDEYKVPSKKFKVLGNTDRHSIKK
NLIGALLFDSGETAEATRLKRTARRRYTRRKNRICYLQEIFSNEMAKVDDSFFHRLEESFLVEEDKKHERHPIFGNIVDEV
AYHEKYPTIYHLRKKLVDSTDKADLRLIYLALAHMIKFRGHFLIEGDLNPDNSDVDKLFIQLVQTYNQLFEENPINASGVD
AKAILSARLSKSRRLENLIAQLPGEKKNGLFGNLIALSLGLTPNFKSNFDLAEDAKLQLSKDTYDDDLDNLLAQIGDQYAD
LFLAAKNLSDAILLSDILRVNTEITKAPLSASMIKRYDEHHQDLTLLKALVRQQLPEKYKEIFFDQSKNGYAGYIDGGASQ
EEFYKFIKPILEKMDGTEELLVKLNREDLLRKQRTFDNGSIPHQIHLGELHAILRRQEDFYPFLKDNREKIEKILTFRIPY
YVGPLARGNSRFAWMTRKSEETITPWNFEEVVDKGASAQSFIERMTNFDKNLPNEKVLPKHSLLYEYFTVYNELTKVKYVT
EGMRKPAFLSGEQKKAIVDLLFKTNRKVTVKQLKEDYFKKIECFDSVEISGVEDRFNASLGTYHDLLKIIKDKDFLDNEEN
EDILEDIVLTLTLFEDREMIEERLKTYAHLFDDKVMKQLKRRRYTGWGRLSRKLINGIRDKQSGKTILDFLKSDGFANRNF
MQLIHDDSLTFKEDIQKAQVSGQGDSLHEHIANLAGSPAIKKGILQTVKVVDELVKVMGRHKPENIVIEMARENQTTQKGQ
KNSRERMKRIEEGIKELGSQILKEHPVENTQLQNEKLYLYYLQNGRDMYVDQELDINRLSDYDVDHIVPQSFLKDDSIDNK
VLTRSDKNRGKSDNVPSEEVVKKMKNYWRQLLNAKLITQRKFDNLTKAERGGLSELDKAGFIKRQLVETRQITKHVAQILD
SRMNTKYDENDKLIREVKVITLKSKLVSDFRKDFQFYKVREINNYHHAHDAYLNAVVGTALIKKYPKLESEFVYGDYKVYD
VRKMIAKSEQEIGKATAKYFFYSNIMNFFKTEITLANGEIRKRPLIETNGETGEIVWDKGRDFATVRKVLSMPQVNIVKKT
EVQTGGFSKESILPKRNSDKLIARKKDWDPKKYGGFDSPTVAYSVLVVAKVEKGKSKKLKSVKELLGITIMERSSFEKNPI
DFLEAKGYKEVKKDLIIKLPKYSLFELENGRKRMLASAGELQKGNELALPSKYVNFLYLASHYEKLKGSPEDNEQKQLFVE
QHKHYLDEIIEQISEFSKRVILADANLDKVLSAYNKHRDKPIREQAENIIHLFTLTNLGAPAAFKYFDTTIDRKRYTSTKE
VLDATLIHQSITGLYETRIDLSQLGGDSGGSGGSGGSTNLSDIIEKETGKQLVIQESILMLPEEVEEVIGNKPESDILVHT
AYDESTDENVMLLTSDAPEYKPWALVIQDSNGENKIKMLSGGSGGSGGSTNLSDIIEKETGKQLVIQESILMLPEEVEEVI
GNKPESDILVHTAYDESTDENVMLLTSDAPEYKPWALVIQDSNGENKIKMLPKKKRKVEGADKRTADGSEFESPKKKRKV*

Supplementary Sequence 15: CABE-T3.155

MSEVEFSHEYWMRHALTLAKRARDERSVPVGAVLVLNNRVIGEGWNRAIGLHDPTAHAEIMALRQGGLVMQNYRLYDATLY
TTFEPCVMCAGAMIHSRIGRVVFGVRNAKTGAAGSLMDVLHHPGMNHRVEITEGILADECAALLCRFFRMPRRVFNAQKKA
QSSTDSGGSSGGSSGSETPGTSESATPESSGGSSGGSDKKYSIGLAIGTNSVGWAVITDEYKVPSKKFKVLGNTDRHSIKK
NLIGALLFDSGETAEATRLKRTARRRYTRRKNRICYLQEIFSNEMAKVDDSFFHRLEESFLVEEDKKHERHPIFGNIVDEV
AYHEKYPTIYHLRKKLVDSTDKADLRLIYLALAHMIKFRGHFLIEGDLNPDNSDVDKLFIQLVQTYNQLFEENPINASGVD
AKAILSARLSKSRRLENLIAQLPGEKKNGLFGNLIALSLGLTPNFKSNFDLAEDAKLQLSKDTYDDDLDNLLAQIGDQYAD
LFLAAKNLSDAILLSDILRVNTEITKAPLSASMIKRYDEHHQDLTLLKALVRQQLPEKYKEIFFDQSKNGYAGYIDGGASQ
EEFYKFIKPILEKMDGTEELLVKLNREDLLRKQRTFDNGSIPHQIHLGELHAILRRQEDFYPFLKDNREKIEKILTFRIPY
YVGPLARGNSRFAWMTRKSEETITPWNFEEVVDKGASAQSFIERMTNFDKNLPNEKVLPKHSLLYEYFTVYNELTKVKYVT
EGMRKPAFLSGEQKKAIVDLLFKTNRKVTVKQLKEDYFKKIECFDSVEISGVEDRFNASLGTYHDLLKIIKDKDFLDNEEN
EDILEDIVLTLTLFEDREMIEERLKTYAHLFDDKVMKQLKRRRYTGWGRLSRKLINGIRDKQSGKTILDFLKSDGFANRNF
MQLIHDDSLTFKEDIQKAQVSGQGDSLHEHIANLAGSPAIKKGILQTVKVVDELVKVMGRHKPENIVIEMARENQTTQKGQ
KNSRERMKRIEEGIKELGSQILKEHPVENTQLQNEKLYLYYLQNGRDMYVDQELDINRLSDYDVDHIVPQSFLKDDSIDNK
VLTRSDKNRGKSDNVPSEEVVKKMKNYWRQLLNAKLITQRKFDNLTKAERGGLSELDKAGFIKRQLVETRQITKHVAQILD
SRMNTKYDENDKLIREVKVITLKSKLVSDFRKDFQFYKVREINNYHHAHDAYLNAVVGTALIKKYPKLESEFVYGDYKVYD
VRKMIAKSEQEIGKATAKYFFYSNIMNFFKTEITLANGEIRKRPLIETNGETGEIVWDKGRDFATVRKVLSMPQVNIVKKT
EVQTGGFSKESILPKRNSDKLIARKKDWDPKKYGGFDSPTVAYSVLVVAKVEKGKSKKLKSVKELLGITIMERSSFEKNPI
DFLEAKGYKEVKKDLIIKLPKYSLFELENGRKRMLASAGELQKGNELALPSKYVNFLYLASHYEKLKGSPEDNEQKQLFVE
QHKHYLDEIIEQISEFSKRVILADANLDKVLSAYNKHRDKPIREQAENIIHLFTLTNLGAPAAFKYFDTTIDRKRYTSTKE
VLDATLIHQSITGLYETRIDLSQLGGDSGGSGGSGGSTNLSDIIEKETGKQLVIQESILMLPEEVEEVIGNKPESDILVHT
AYDESTDENVMLLTSDAPEYKPWALVIQDSNGENKIKMLSGGSGGSGGSTNLSDIIEKETGKQLVIQESILMLPEEVEEVI
GNKPESDILVHTAYDESTDENVMLLTSDAPEYKPWALVIQDSNGENKIKMLPKKKRKVEGADKRTADGSEFESPKKKRKV*

Supplementary Sequence 16: CBE-T1.2

MSEVEYSHEYWMRHALTLAKRARDERHVPVGAVLVLNNRVIGEGWNRAKGLHDPTAHAEIMALRQGGLVMQNYRLYGATLY
TTFEPCVMCAGAMIHSRIGRVVFGVCNAKTHAAMSLMDVLHHPGMNHRVEITEGILADECEALLCRFFRMPRRVFNAQKKA
QSSTDSGGSSGGSSGSETPGTSESATPESSGGSSGGSDKKYSIGLAIGTNSVGWAVITDEYKVPSKKFKVLGNTDRHSIKK
NLIGALLFDSGETAEATRLKRTARRRYTRRKNRICYLQEIFSNEMAKVDDSFFHRLEESFLVEEDKKHERHPIFGNIVDEV
AYHEKYPTIYHLRKKLVDSTDKADLRLIYLALAHMIKFRGHFLIEGDLNPDNSDVDKLFIQLVQTYNQLFEENPINASGVD
AKAILSARLSKSRRLENLIAQLPGEKKNGLFGNLIALSLGLTPNFKSNFDLAEDAKLQLSKDTYDDDLDNLLAQIGDQYAD
LFLAAKNLSDAILLSDILRVNTEITKAPLSASMIKRYDEHHQDLTLLKALVRQQLPEKYKEIFFDQSKNGYAGYIDGGASQ
EEFYKFIKPILEKMDGTEELLVKLNREDLLRKQRTFDNGSIPHQIHLGELHAILRRQEDFYPFLKDNREKIEKILTFRIPY
YVGPLARGNSRFAWMTRKSEETITPWNFEEVVDKGASAQSFIERMTNFDKNLPNEKVLPKHSLLYEYFTVYNELTKVKYVT
EGMRKPAFLSGEQKKAIVDLLFKTNRKVTVKQLKEDYFKKIECFDSVEISGVEDRFNASLGTYHDLLKIIKDKDFLDNEEN
EDILEDIVLTLTLFEDREMIEERLKTYAHLFDDKVMKQLKRRRYTGWGRLSRKLINGIRDKQSGKTILDFLKSDGFANRNF
MQLIHDDSLTFKEDIQKAQVSGQGDSLHEHIANLAGSPAIKKGILQTVKVVDELVKVMGRHKPENIVIEMARENQTTQKGQ
KNSRERMKRIEEGIKELGSQILKEHPVENTQLQNEKLYLYYLQNGRDMYVDQELDINRLSDYDVDHIVPQSFLKDDSIDNK
VLTRSDKNRGKSDNVPSEEVVKKMKNYWRQLLNAKLITQRKFDNLTKAERGGLSELDKAGFIKRQLVETRQITKHVAQILD
SRMNTKYDENDKLIREVKVITLKSKLVSDFRKDFQFYKVREINNYHHAHDAYLNAVVGTALIKKYPKLESEFVYGDYKVYD
VRKMIAKSEQEIGKATAKYFFYSNIMNFFKTEITLANGEIRKRPLIETNGETGEIVWDKGRDFATVRKVLSMPQVNIVKKT
EVQTGGFSKESILPKRNSDKLIARKKDWDPKKYGGFDSPTVAYSVLVVAKVEKGKSKKLKSVKELLGITIMERSSFEKNPI
DFLEAKGYKEVKKDLIIKLPKYSLFELENGRKRMLASAGELQKGNELALPSKYVNFLYLASHYEKLKGSPEDNEQKQLFVE
QHKHYLDEIIEQISEFSKRVILADANLDKVLSAYNKHRDKPIREQAENIIHLFTLTNLGAPAAFKYFDTTIDRKRYTSTKE
VLDATLIHQSITGLYETRIDLSQLGGDSGGSGGSGGSTNLSDIIEKETGKQLVIQESILMLPEEVEEVIGNKPESDILVHT
AYDESTDENVMLLTSDAPEYKPWALVIQDSNGENKIKMLSGGSGGSGGSTNLSDIIEKETGKQLVIQESILMLPEEVEEVI
GNKPESDILVHTAYDESTDENVMLLTSDAPEYKPWALVIQDSNGENKIKMLPKKKRKVEGADKRTADGSEFESPKKKRKV*

Supplementary Sequence 17: CBE-T1.3

MSEVEYSHEYWMRHALTLAKRARDERHVPVGAVLVLNNRVIGEGWNRAKGLHDPTAHAEIMALRQGGLVMQNYRLYDATLY
TTFEPCVMCAGAMIHSRIGRVVFGVCNAKTHACMSLMDVLHHPGMNHRVEITEGILADECEALLCRFFRMPRRVFNAQKKA
QSSTDSGGSSGGSSGSETPGTSESATPESSGGSSGGSDKKYSIGLAIGTNSVGWAVITDEYKVPSKKFKVLGNTDRHSIKK
NLIGALLFDSGETAEATRLKRTARRRYTRRKNRICYLQEIFSNEMAKVDDSFFHRLEESFLVEEDKKHERHPIFGNIVDEV
AYHEKYPTIYHLRKKLVDSTDKADLRLIYLALAHMIKFRGHFLIEGDLNPDNSDVDKLFIQLVQTYNQLFEENPINASGVD
AKAILSARLSKSRRLENLIAQLPGEKKNGLFGNLIALSLGLTPNFKSNFDLAEDAKLQLSKDTYDDDLDNLLAQIGDQYAD
LFLAAKNLSDAILLSDILRVNTEITKAPLSASMIKRYDEHHQDLTLLKALVRQQLPEKYKEIFFDQSKNGYAGYIDGGASQ
EEFYKFIKPILEKMDGTEELLVKLNREDLLRKQRTFDNGSIPHQIHLGELHAILRRQEDFYPFLKDNREKIEKILTFRIPY
YVGPLARGNSRFAWMTRKSEETITPWNFEEVVDKGASAQSFIERMTNFDKNLPNEKVLPKHSLLYEYFTVYNELTKVKYVT
EGMRKPAFLSGEQKKAIVDLLFKTNRKVTVKQLKEDYFKKIECFDSVEISGVEDRFNASLGTYHDLLKIIKDKDFLDNEEN
EDILEDIVLTLTLFEDREMIEERLKTYAHLFDDKVMKQLKRRRYTGWGRLSRKLINGIRDKQSGKTILDFLKSDGFANRNF
MQLIHDDSLTFKEDIQKAQVSGQGDSLHEHIANLAGSPAIKKGILQTVKVVDELVKVMGRHKPENIVIEMARENQTTQKGQ
KNSRERMKRIEEGIKELGSQILKEHPVENTQLQNEKLYLYYLQNGRDMYVDQELDINRLSDYDVDHIVPQSFLKDDSIDNK
VLTRSDKNRGKSDNVPSEEVVKKMKNYWRQLLNAKLITQRKFDNLTKAERGGLSELDKAGFIKRQLVETRQITKHVAQILD
SRMNTKYDENDKLIREVKVITLKSKLVSDFRKDFQFYKVREINNYHHAHDAYLNAVVGTALIKKYPKLESEFVYGDYKVYD
VRKMIAKSEQEIGKATAKYFFYSNIMNFFKTEITLANGEIRKRPLIETNGETGEIVWDKGRDFATVRKVLSMPQVNIVKKT
EVQTGGFSKESILPKRNSDKLIARKKDWDPKKYGGFDSPTVAYSVLVVAKVEKGKSKKLKSVKELLGITIMERSSFEKNPI
DFLEAKGYKEVKKDLIIKLPKYSLFELENGRKRMLASAGELQKGNELALPSKYVNFLYLASHYEKLKGSPEDNEQKQLFVE
QHKHYLDEIIEQISEFSKRVILADANLDKVLSAYNKHRDKPIREQAENIIHLFTLTNLGAPAAFKYFDTTIDRKRYTSTKE
VLDATLIHQSITGLYETRIDLSQLGGDSGGSGGSGGSTNLSDIIEKETGKQLVIQESILMLPEEVEEVIGNKPESDILVHT
AYDESTDENVMLLTSDAPEYKPWALVIQDSNGENKIKMLSGGSGGSGGSTNLSDIIEKETGKQLVIQESILMLPEEVEEVI
GNKPESDILVHTAYDESTDENVMLLTSDAPEYKPWALVIQDSNGENKIKMLPKKKRKVEGADKRTADGSEFESPKKKRKV*

Supplementary Sequence 18: CBE-T1.4

MSEVEYSHEYWMRHALTLAKRARDERHVPVGAVLVLNNRVIGEGWNRAKGLHDPTAHAEIMALRQGGLVMQNYRLYDATLY
TTFEPCVMCAGAMIHSRIGRVVFGVCNAKTHAAMSLMNVLHHPGMNHRVEITEGILADECEALLCRFFRMPRRVFNAQKKA
QSSTDSGGSSGGSSGSETPGTSESATPESSGGSSGGSDKKYSIGLAIGTNSVGWAVITDEYKVPSKKFKVLGNTDRHSIKK
NLIGALLFDSGETAEATRLKRTARRRYTRRKNRICYLQEIFSNEMAKVDDSFFHRLEESFLVEEDKKHERHPIFGNIVDEV
AYHEKYPTIYHLRKKLVDSTDKADLRLIYLALAHMIKFRGHFLIEGDLNPDNSDVDKLFIQLVQTYNQLFEENPINASGVD
AKAILSARLSKSRRLENLIAQLPGEKKNGLFGNLIALSLGLTPNFKSNFDLAEDAKLQLSKDTYDDDLDNLLAQIGDQYAD
LFLAAKNLSDAILLSDILRVNTEITKAPLSASMIKRYDEHHQDLTLLKALVRQQLPEKYKEIFFDQSKNGYAGYIDGGASQ
EEFYKFIKPILEKMDGTEELLVKLNREDLLRKQRTFDNGSIPHQIHLGELHAILRRQEDFYPFLKDNREKIEKILTFRIPY
YVGPLARGNSRFAWMTRKSEETITPWNFEEVVDKGASAQSFIERMTNFDKNLPNEKVLPKHSLLYEYFTVYNELTKVKYVT
EGMRKPAFLSGEQKKAIVDLLFKTNRKVTVKQLKEDYFKKIECFDSVEISGVEDRFNASLGTYHDLLKIIKDKDFLDNEEN
EDILEDIVLTLTLFEDREMIEERLKTYAHLFDDKVMKQLKRRRYTGWGRLSRKLINGIRDKQSGKTILDFLKSDGFANRNF
MQLIHDDSLTFKEDIQKAQVSGQGDSLHEHIANLAGSPAIKKGILQTVKVVDELVKVMGRHKPENIVIEMARENQTTQKGQ
KNSRERMKRIEEGIKELGSQILKEHPVENTQLQNEKLYLYYLQNGRDMYVDQELDINRLSDYDVDHIVPQSFLKDDSIDNK
VLTRSDKNRGKSDNVPSEEVVKKMKNYWRQLLNAKLITQRKFDNLTKAERGGLSELDKAGFIKRQLVETRQITKHVAQILD
SRMNTKYDENDKLIREVKVITLKSKLVSDFRKDFQFYKVREINNYHHAHDAYLNAVVGTALIKKYPKLESEFVYGDYKVYD
VRKMIAKSEQEIGKATAKYFFYSNIMNFFKTEITLANGEIRKRPLIETNGETGEIVWDKGRDFATVRKVLSMPQVNIVKKT
EVQTGGFSKESILPKRNSDKLIARKKDWDPKKYGGFDSPTVAYSVLVVAKVEKGKSKKLKSVKELLGITIMERSSFEKNPI
DFLEAKGYKEVKKDLIIKLPKYSLFELENGRKRMLASAGELQKGNELALPSKYVNFLYLASHYEKLKGSPEDNEQKQLFVE
QHKHYLDEIIEQISEFSKRVILADANLDKVLSAYNKHRDKPIREQAENIIHLFTLTNLGAPAAFKYFDTTIDRKRYTSTKE
VLDATLIHQSITGLYETRIDLSQLGGDSGGSGGSGGSTNLSDIIEKETGKQLVIQESILMLPEEVEEVIGNKPESDILVHT
AYDESTDENVMLLTSDAPEYKPWALVIQDSNGENKIKMLSGGSGGSGGSTNLSDIIEKETGKQLVIQESILMLPEEVEEVI
GNKPESDILVHTAYDESTDENVMLLTSDAPEYKPWALVIQDSNGENKIKMLPKKKRKVEGADKRTADGSEFESPKKKRKV*

Supplementary Sequence 19: CBE-T1.5

MSEVEYSHEYWMRHALTLAKRARDERHVPVGAVLVLNNRVIGEGWNRAKGLHDPTAHAEIMALRQGGLVMQNYRLYDATLY
TTFEPCVMCAGAMIHSRIGRVVFGVCNAKTHAAMSLMDVLGHPGMNHRVEITEGILADECEALLCRFFRMPRRVFNAQKKA
QSSTDSGGSSGGSSGSETPGTSESATPESSGGSSGGSDKKYSIGLAIGTNSVGWAVITDEYKVPSKKFKVLGNTDRHSIKK
NLIGALLFDSGETAEATRLKRTARRRYTRRKNRICYLQEIFSNEMAKVDDSFFHRLEESFLVEEDKKHERHPIFGNIVDEV
AYHEKYPTIYHLRKKLVDSTDKADLRLIYLALAHMIKFRGHFLIEGDLNPDNSDVDKLFIQLVQTYNQLFEENPINASGVD
AKAILSARLSKSRRLENLIAQLPGEKKNGLFGNLIALSLGLTPNFKSNFDLAEDAKLQLSKDTYDDDLDNLLAQIGDQYAD
LFLAAKNLSDAILLSDILRVNTEITKAPLSASMIKRYDEHHQDLTLLKALVRQQLPEKYKEIFFDQSKNGYAGYIDGGASQ
EEFYKFIKPILEKMDGTEELLVKLNREDLLRKQRTFDNGSIPHQIHLGELHAILRRQEDFYPFLKDNREKIEKILTFRIPY
YVGPLARGNSRFAWMTRKSEETITPWNFEEVVDKGASAQSFIERMTNFDKNLPNEKVLPKHSLLYEYFTVYNELTKVKYVT
EGMRKPAFLSGEQKKAIVDLLFKTNRKVTVKQLKEDYFKKIECFDSVEISGVEDRFNASLGTYHDLLKIIKDKDFLDNEEN
EDILEDIVLTLTLFEDREMIEERLKTYAHLFDDKVMKQLKRRRYTGWGRLSRKLINGIRDKQSGKTILDFLKSDGFANRNF
MQLIHDDSLTFKEDIQKAQVSGQGDSLHEHIANLAGSPAIKKGILQTVKVVDELVKVMGRHKPENIVIEMARENQTTQKGQ
KNSRERMKRIEEGIKELGSQILKEHPVENTQLQNEKLYLYYLQNGRDMYVDQELDINRLSDYDVDHIVPQSFLKDDSIDNK
VLTRSDKNRGKSDNVPSEEVVKKMKNYWRQLLNAKLITQRKFDNLTKAERGGLSELDKAGFIKRQLVETRQITKHVAQILD
SRMNTKYDENDKLIREVKVITLKSKLVSDFRKDFQFYKVREINNYHHAHDAYLNAVVGTALIKKYPKLESEFVYGDYKVYD
VRKMIAKSEQEIGKATAKYFFYSNIMNFFKTEITLANGEIRKRPLIETNGETGEIVWDKGRDFATVRKVLSMPQVNIVKKT
EVQTGGFSKESILPKRNSDKLIARKKDWDPKKYGGFDSPTVAYSVLVVAKVEKGKSKKLKSVKELLGITIMERSSFEKNPI
DFLEAKGYKEVKKDLIIKLPKYSLFELENGRKRMLASAGELQKGNELALPSKYVNFLYLASHYEKLKGSPEDNEQKQLFVE
QHKHYLDEIIEQISEFSKRVILADANLDKVLSAYNKHRDKPIREQAENIIHLFTLTNLGAPAAFKYFDTTIDRKRYTSTKE
VLDATLIHQSITGLYETRIDLSQLGGDSGGSGGSGGSTNLSDIIEKETGKQLVIQESILMLPEEVEEVIGNKPESDILVHT
AYDESTDENVMLLTSDAPEYKPWALVIQDSNGENKIKMLSGGSGGSGGSTNLSDIIEKETGKQLVIQESILMLPEEVEEVI
GNKPESDILVHTAYDESTDENVMLLTSDAPEYKPWALVIQDSNGENKIKMLPKKKRKVEGADKRTADGSEFESPKKKRKV*

Supplementary Sequence 20: CBE-T1.6

MSEVEYSHEYWMRHALTLAKRARDERHVPVGAVLVLNNRVIGEGWNRAKGLHDPTAHAEIMALRQGGLVMQNYRLYDATLY
TTFEPCVMCAGAMIHSRIGRVVFGVCNAKTHAAMSLMDVLHHPGMPHRVEITEGILADECEALLCRFFRMPRRVFNAQKKA
QSSTDSGGSSGGSSGSETPGTSESATPESSGGSSGGSDKKYSIGLAIGTNSVGWAVITDEYKVPSKKFKVLGNTDRHSIKK
NLIGALLFDSGETAEATRLKRTARRRYTRRKNRICYLQEIFSNEMAKVDDSFFHRLEESFLVEEDKKHERHPIFGNIVDEV
AYHEKYPTIYHLRKKLVDSTDKADLRLIYLALAHMIKFRGHFLIEGDLNPDNSDVDKLFIQLVQTYNQLFEENPINASGVD
AKAILSARLSKSRRLENLIAQLPGEKKNGLFGNLIALSLGLTPNFKSNFDLAEDAKLQLSKDTYDDDLDNLLAQIGDQYAD
LFLAAKNLSDAILLSDILRVNTEITKAPLSASMIKRYDEHHQDLTLLKALVRQQLPEKYKEIFFDQSKNGYAGYIDGGASQ
EEFYKFIKPILEKMDGTEELLVKLNREDLLRKQRTFDNGSIPHQIHLGELHAILRRQEDFYPFLKDNREKIEKILTFRIPY
YVGPLARGNSRFAWMTRKSEETITPWNFEEVVDKGASAQSFIERMTNFDKNLPNEKVLPKHSLLYEYFTVYNELTKVKYVT
EGMRKPAFLSGEQKKAIVDLLFKTNRKVTVKQLKEDYFKKIECFDSVEISGVEDRFNASLGTYHDLLKIIKDKDFLDNEEN
EDILEDIVLTLTLFEDREMIEERLKTYAHLFDDKVMKQLKRRRYTGWGRLSRKLINGIRDKQSGKTILDFLKSDGFANRNF
MQLIHDDSLTFKEDIQKAQVSGQGDSLHEHIANLAGSPAIKKGILQTVKVVDELVKVMGRHKPENIVIEMARENQTTQKGQ
KNSRERMKRIEEGIKELGSQILKEHPVENTQLQNEKLYLYYLQNGRDMYVDQELDINRLSDYDVDHIVPQSFLKDDSIDNK
VLTRSDKNRGKSDNVPSEEVVKKMKNYWRQLLNAKLITQRKFDNLTKAERGGLSELDKAGFIKRQLVETRQITKHVAQILD
SRMNTKYDENDKLIREVKVITLKSKLVSDFRKDFQFYKVREINNYHHAHDAYLNAVVGTALIKKYPKLESEFVYGDYKVYD
VRKMIAKSEQEIGKATAKYFFYSNIMNFFKTEITLANGEIRKRPLIETNGETGEIVWDKGRDFATVRKVLSMPQVNIVKKT
EVQTGGFSKESILPKRNSDKLIARKKDWDPKKYGGFDSPTVAYSVLVVAKVEKGKSKKLKSVKELLGITIMERSSFEKNPI
DFLEAKGYKEVKKDLIIKLPKYSLFELENGRKRMLASAGELQKGNELALPSKYVNFLYLASHYEKLKGSPEDNEQKQLFVE
QHKHYLDEIIEQISEFSKRVILADANLDKVLSAYNKHRDKPIREQAENIIHLFTLTNLGAPAAFKYFDTTIDRKRYTSTKE
VLDATLIHQSITGLYETRIDLSQLGGDSGGSGGSGGSTNLSDIIEKETGKQLVIQESILMLPEEVEEVIGNKPESDILVHT
AYDESTDENVMLLTSDAPEYKPWALVIQDSNGENKIKMLSGGSGGSGGSTNLSDIIEKETGKQLVIQESILMLPEEVEEVI
GNKPESDILVHTAYDESTDENVMLLTSDAPEYKPWALVIQDSNGENKIKMLPKKKRKVEGADKRTADGSEFESPKKKRKV*

Supplementary Sequence 21: CBE-T1.13

MSEVEYSHEYWMRHALTLAKRARDERHVPVGAVLVLNNRVIGEGWNRAKGLHDPTAHAEIMALRQGGLVMQNYRLWDATLY
TTFEPCVMCAGAMIHSRIGRVVFGVCNAKTHAAMSLMDVLHHPGMPHRVEITEGILADECEALLCRFFRMPRRVFNAQKKA
QSSTDSGGSSGGSSGSETPGTSESATPESSGGSSGGSDKKYSIGLAIGTNSVGWAVITDEYKVPSKKFKVLGNTDRHSIKK
NLIGALLFDSGETAEATRLKRTARRRYTRRKNRICYLQEIFSNEMAKVDDSFFHRLEESFLVEEDKKHERHPIFGNIVDEV
AYHEKYPTIYHLRKKLVDSTDKADLRLIYLALAHMIKFRGHFLIEGDLNPDNSDVDKLFIQLVQTYNQLFEENPINASGVD
AKAILSARLSKSRRLENLIAQLPGEKKNGLFGNLIALSLGLTPNFKSNFDLAEDAKLQLSKDTYDDDLDNLLAQIGDQYAD
LFLAAKNLSDAILLSDILRVNTEITKAPLSASMIKRYDEHHQDLTLLKALVRQQLPEKYKEIFFDQSKNGYAGYIDGGASQ
EEFYKFIKPILEKMDGTEELLVKLNREDLLRKQRTFDNGSIPHQIHLGELHAILRRQEDFYPFLKDNREKIEKILTFRIPY
YVGPLARGNSRFAWMTRKSEETITPWNFEEVVDKGASAQSFIERMTNFDKNLPNEKVLPKHSLLYEYFTVYNELTKVKYVT
EGMRKPAFLSGEQKKAIVDLLFKTNRKVTVKQLKEDYFKKIECFDSVEISGVEDRFNASLGTYHDLLKIIKDKDFLDNEEN
EDILEDIVLTLTLFEDREMIEERLKTYAHLFDDKVMKQLKRRRYTGWGRLSRKLINGIRDKQSGKTILDFLKSDGFANRNF
MQLIHDDSLTFKEDIQKAQVSGQGDSLHEHIANLAGSPAIKKGILQTVKVVDELVKVMGRHKPENIVIEMARENQTTQKGQ
KNSRERMKRIEEGIKELGSQILKEHPVENTQLQNEKLYLYYLQNGRDMYVDQELDINRLSDYDVDHIVPQSFLKDDSIDNK
VLTRSDKNRGKSDNVPSEEVVKKMKNYWRQLLNAKLITQRKFDNLTKAERGGLSELDKAGFIKRQLVETRQITKHVAQILD
SRMNTKYDENDKLIREVKVITLKSKLVSDFRKDFQFYKVREINNYHHAHDAYLNAVVGTALIKKYPKLESEFVYGDYKVYD
VRKMIAKSEQEIGKATAKYFFYSNIMNFFKTEITLANGEIRKRPLIETNGETGEIVWDKGRDFATVRKVLSMPQVNIVKKT
EVQTGGFSKESILPKRNSDKLIARKKDWDPKKYGGFDSPTVAYSVLVVAKVEKGKSKKLKSVKELLGITIMERSSFEKNPI
DFLEAKGYKEVKKDLIIKLPKYSLFELENGRKRMLASAGELQKGNELALPSKYVNFLYLASHYEKLKGSPEDNEQKQLFVE
QHKHYLDEIIEQISEFSKRVILADANLDKVLSAYNKHRDKPIREQAENIIHLFTLTNLGAPAAFKYFDTTIDRKRYTSTKE
VLDATLIHQSITGLYETRIDLSQLGGDSGGSGGSGGSTNLSDIIEKETGKQLVIQESILMLPEEVEEVIGNKPESDILVHT
AYDESTDENVMLLTSDAPEYKPWALVIQDSNGENKIKMLSGGSGGSGGSTNLSDIIEKETGKQLVIQESILMLPEEVEEVI
GNKPESDILVHTAYDESTDENVMLLTSDAPEYKPWALVIQDSNGENKIKMLPKKKRKVEGADKRTADGSEFESPKKKRKV*

Supplementary Sequence 22: CBE-T1.14

MSEVEYSHEYWMRHALTLAKRARDERHVPVGAVLVLNNRVIGEGWNRAKGLHDPTAHAEIMALRQGGLVMQNYRL
WDATLYTTFEPCVMCAGAMIHSRIGRVVFGVCNAKTHAAMSLMDVLHHPGMNHRVEITEGILADECEELLCRFFRMPRRVF
NAQKKAQSSTDSGGSSGGSSGSETPGTSESATPESSGGSSGGSDKKYSIGLAIGTNSVGWAVITDEYKVPSKKFKVLGNTD
RHSIKKNLIGALLFDSGETAEATRLKRTARRRYTRRKNRICYLQEIFSNEMAKVDDSFFHRLEESFLVEEDKKHERHPIFG
NIVDEVAYHEKYPTIYHLRKKLVDSTDKADLRLIYLALAHMIKFRGHFLIEGDLNPDNSDVDKLFIQLVQTYNQLFEENPI
NASGVDAKAILSARLSKSRRLENLIAQLPGEKKNGLFGNLIALSLGLTPNFKSNFDLAEDAKLQLSKDTYDDDLDNLLAQI
GDQYADLFLAAKNLSDAILLSDILRVNTEITKAPLSASMIKRYDEHHQDLTLLKALVRQQLPEKYKEIFFDQSKNGYAGYI
DGGASQEEFYKFIKPILEKMDGTEELLVKLNREDLLRKQRTFDNGSIPHQIHLGELHAILRRQEDFYPFLKDNREKIEKIL
TFRIPYYVGPLARGNSRFAWMTRKSEETITPWNFEEVVDKGASAQSFIERMTNFDKNLPNEKVLPKHSLLYEYFTVYNELT
KVKYVTEGMRKPAFLSGEQKKAIVDLLFKTNRKVTVKQLKEDYFKKIECFDSVEISGVEDRFNASLGTYHDLLKIIKDKDF
LDNEENEDILEDIVLTLTLFEDREMIEERLKTYAHLFDDKVMKQLKRRRYTGWGRLSRKLINGIRDKQSGKTILDFLKSDG
FANRNFMQLIHDDSLTFKEDIQKAQVSGQGDSLHEHIANLAGSPAIKKGILQTVKVVDELVKVMGRHKPENIVIEMARENQ
TTQKGQKNSRERMKRIEEGIKELGSQILKEHPVENTQLQNEKLYLYYLQNGRDMYVDQELDINRLSDYDVDHIVPQSFLKD
DSIDNKVLTRSDKNRGKSDNVPSEEVVKKMKNYWRQLLNAKLITQRKFDNLTKAERGGLSELDKAGFIKRQLVETRQITKH
VAQILDSRMNTKYDENDKLIREVKVITLKSKLVSDFRKDFQFYKVREINNYHHAHDAYLNAVVGTALIKKYPKLESEFVYG
DYKVYDVRKMIAKSEQEIGKATAKYFFYSNIMNFFKTEITLANGEIRKRPLIETNGETGEIVWDKGRDFATVRKVLSMPQV
NIVKKTEVQTGGFSKESILPKRNSDKLIARKKDWDPKKYGGFDSPTVAYSVLVVAKVEKGKSKKLKSVKELLGITIMERSS
FEKNPIDFLEAKGYKEVKKDLIIKLPKYSLFELENGRKRMLASAGELQKGNELALPSKYVNFLYLASHYEKLKGSPEDNEQ
KQLFVEQHKHYLDEIIEQISEFSKRVILADANLDKVLSAYNKHRDKPIREQAENIIHLFTLTNLGAPAAFKYFDTTIDRKR
YTSTKEVLDATLIHQSITGLYETRIDLSQLGGDSGGSGGSGGSTNLSDIIEKETGKQLVIQESILMLPEEVEEVIGNKPES
DILVHTAYDESTDENVMLLTSDAPEYKPWALVIQDSNGENKIKMLSGGSGGSGGSTNLSDIIEKETGKQLVIQESILMLPE
EVEEVIGNKPESDILVHTAYDESTDENVMLLTSDAPEYKPWALVIQDSNGENKIKMLPKKKRKVEGADKRTADGSEFESPK
KKRKV*

Supplementary Sequence 23: CBE-T1.21

MSEVEYSHEYWMRHALTLAKRARDERHVPVGAVLVLNNRVIGEGWNRAKGLHDPTAHAEIMALRQGGLVMQNYRLYGATLY
TTFEPCVMCAGAMIHSRIGRVVFGVCNAKTHAAMSLMDVLHHPGMNHRVEITEGILADECAELLCRFFRMPRRVFNAQKKA
QSSTDSGGSSGGSSGSETPGTSESATPESSGGSSGGSDKKYSIGLAIGTNSVGWAVITDEYKVPSKKFKVLGNTDRHSIKK
NLIGALLFDSGETAEATRLKRTARRRYTRRKNRICYLQEIFSNEMAKVDDSFFHRLEESFLVEEDKKHERHPIFGNIVDEV
AYHEKYPTIYHLRKKLVDSTDKADLRLIYLALAHMIKFRGHFLIEGDLNPDNSDVDKLFIQLVQTYNQLFEENPINASGVD
AKAILSARLSKSRRLENLIAQLPGEKKNGLFGNLIALSLGLTPNFKSNFDLAEDAKLQLSKDTYDDDLDNLLAQIGDQYAD
LFLAAKNLSDAILLSDILRVNTEITKAPLSASMIKRYDEHHQDLTLLKALVRQQLPEKYKEIFFDQSKNGYAGYIDGGASQ
EEFYKFIKPILEKMDGTEELLVKLNREDLLRKQRTFDNGSIPHQIHLGELHAILRRQEDFYPFLKDNREKIEKILTFRIPY
YVGPLARGNSRFAWMTRKSEETITPWNFEEVVDKGASAQSFIERMTNFDKNLPNEKVLPKHSLLYEYFTVYNELTKVKYVT
EGMRKPAFLSGEQKKAIVDLLFKTNRKVTVKQLKEDYFKKIECFDSVEISGVEDRFNASLGTYHDLLKIIKDKDFLDNEEN
EDILEDIVLTLTLFEDREMIEERLKTYAHLFDDKVMKQLKRRRYTGWGRLSRKLINGIRDKQSGKTILDFLKSDGFANRNF
MQLIHDDSLTFKEDIQKAQVSGQGDSLHEHIANLAGSPAIKKGILQTVKVVDELVKVMGRHKPENIVIEMARENQTTQKGQ
KNSRERMKRIEEGIKELGSQILKEHPVENTQLQNEKLYLYYLQNGRDMYVDQELDINRLSDYDVDHIVPQSFLKDDSIDNK
VLTRSDKNRGKSDNVPSEEVVKKMKNYWRQLLNAKLITQRKFDNLTKAERGGLSELDKAGFIKRQLVETRQITKHVAQILD
SRMNTKYDENDKLIREVKVITLKSKLVSDFRKDFQFYKVREINNYHHAHDAYLNAVVGTALIKKYPKLESEFVYGDYKVYD
VRKMIAKSEQEIGKATAKYFFYSNIMNFFKTEITLANGEIRKRPLIETNGETGEIVWDKGRDFATVRKVLSMPQVNIVKKT
EVQTGGFSKESILPKRNSDKLIARKKDWDPKKYGGFDSPTVAYSVLVVAKVEKGKSKKLKSVKELLGITIMERSSFEKNPI
DFLEAKGYKEVKKDLIIKLPKYSLFELENGRKRMLASAGELQKGNELALPSKYVNFLYLASHYEKLKGSPEDNEQKQLFVE
QHKHYLDEIIEQISEFSKRVILADANLDKVLSAYNKHRDKPIREQAENIIHLFTLTNLGAPAAFKYFDTTIDRKRYTSTKE
VLDATLIHQSITGLYETRIDLSQLGGDSGGSGGSGGSTNLSDIIEKETGKQLVIQESILMLPEEVEEVIGNKPESDILVHT
AYDESTDENVMLLTSDAPEYKPWALVIQDSNGENKIKMLSGGSGGSGGSTNLSDIIEKETGKQLVIQESILMLPEEVEEVI
GNKPESDILVHTAYDESTDENVMLLTSDAPEYKPWALVIQDSNGENKIKMLPKKKRKVEGADKRTADGSEFESPKKKRKV*

Supplementary Sequence 24: CBE-T1.28

MSEVEYSHEYWMRHALTLAKRARDERHVPVGAVLVLNNRVIGEGWNRAKGLHDPTAHAEIMALRQGGLVMQNYRLWGATLY
TTFEPCVMCAGAMIHSRIGRVVFGVCNAKTHACMSLMDVLHHPGMNHRVEITEGILADECAELLCRFFRMPRRVFNAQKKA
QSSTDSGGSSGGSSGSETPGTSESATPESSGGSSGGSDKKYSIGLAIGTNSVGWAVITDEYKVPSKKFKVLGNTDRHSIKK
NLIGALLFDSGETAEATRLKRTARRRYTRRKNRICYLQEIFSNEMAKVDDSFFHRLEESFLVEEDKKHERHPIFGNIVDEV
AYHEKYPTIYHLRKKLVDSTDKADLRLIYLALAHMIKFRGHFLIEGDLNPDNSDVDKLFIQLVQTYNQLFEENPINASGVD
AKAILSARLSKSRRLENLIAQLPGEKKNGLFGNLIALSLGLTPNFKSNFDLAEDAKLQLSKDTYDDDLDNLLAQIGDQYAD
LFLAAKNLSDAILLSDILRVNTEITKAPLSASMIKRYDEHHQDLTLLKALVRQQLPEKYKEIFFDQSKNGYAGYIDGGASQ
EEFYKFIKPILEKMDGTEELLVKLNREDLLRKQRTFDNGSIPHQIHLGELHAILRRQEDFYPFLKDNREKIEKILTFRIPY
YVGPLARGNSRFAWMTRKSEETITPWNFEEVVDKGASAQSFIERMTNFDKNLPNEKVLPKHSLLYEYFTVYNELTKVKYVT
EGMRKPAFLSGEQKKAIVDLLFKTNRKVTVKQLKEDYFKKIECFDSVEISGVEDRFNASLGTYHDLLKIIKDKDFLDNEEN
EDILEDIVLTLTLFEDREMIEERLKTYAHLFDDKVMKQLKRRRYTGWGRLSRKLINGIRDKQSGKTILDFLKSDGFANRNF
MQLIHDDSLTFKEDIQKAQVSGQGDSLHEHIANLAGSPAIKKGILQTVKVVDELVKVMGRHKPENIVIEMARENQTTQKGQ
KNSRERMKRIEEGIKELGSQILKEHPVENTQLQNEKLYLYYLQNGRDMYVDQELDINRLSDYDVDHIVPQSFLKDDSIDNK
VLTRSDKNRGKSDNVPSEEVVKKMKNYWRQLLNAKLITQRKFDNLTKAERGGLSELDKAGFIKRQLVETRQITKHVAQILD
SRMNTKYDENDKLIREVKVITLKSKLVSDFRKDFQFYKVREINNYHHAHDAYLNAVVGTALIKKYPKLESEFVYGDYKVYD
VRKMIAKSEQEIGKATAKYFFYSNIMNFFKTEITLANGEIRKRPLIETNGETGEIVWDKGRDFATVRKVLSMPQVNIVKKT
EVQTGGFSKESILPKRNSDKLIARKKDWDPKKYGGFDSPTVAYSVLVVAKVEKGKSKKLKSVKELLGITIMERSSFEKNPI
DFLEAKGYKEVKKDLIIKLPKYSLFELENGRKRMLASAGELQKGNELALPSKYVNFLYLASHYEKLKGSPEDNEQKQLFVE
QHKHYLDEIIEQISEFSKRVILADANLDKVLSAYNKHRDKPIREQAENIIHLFTLTNLGAPAAFKYFDTTIDRKRYTSTKE
VLDATLIHQSITGLYETRIDLSQLGGDSGGSGGSGGSTNLSDIIEKETGKQLVIQESILMLPEEVEEVIGNKPESDILVHT
AYDESTDENVMLLTSDAPEYKPWALVIQDSNGENKIKMLSGGSGGSGGSTNLSDIIEKETGKQLVIQESILMLPEEVEEVI
GNKPESDILVHTAYDESTDENVMLLTSDAPEYKPWALVIQDSNGENKIKMLPKKKRKVEGADKRTADGSEFESPKKKRKV*

Supplementary Sequence 25: CBE-T1.30

MSEVEYSHEYWMRHALTLAKRARDERHVPVGAVLVLNNRVIGEGWNRAKGLHDPTAHAEIMALRQGGLVMQNYRLWGATLY
TTFEPCVMCAGAMIHSRIGRVVFGVCNAKTHAAMSLMDVLGHPGMNHRVEITEGILADECEALLCRFFRMPRRVFNAQKKA
QSSTDSGGSSGGSSGSETPGTSESATPESSGGSSGGSDKKYSIGLAIGTNSVGWAVITDEYKVPSKKFKVLGNTDRHSIKK
NLIGALLFDSGETAEATRLKRTARRRYTRRKNRICYLQEIFSNEMAKVDDSFFHRLEESFLVEEDKKHERHPIFGNIVDEV
AYHEKYPTIYHLRKKLVDSTDKADLRLIYLALAHMIKFRGHFLIEGDLNPDNSDVDKLFIQLVQTYNQLFEENPINASGVD
AKAILSARLSKSRRLENLIAQLPGEKKNGLFGNLIALSLGLTPNFKSNFDLAEDAKLQLSKDTYDDDLDNLLAQIGDQYAD
LFLAAKNLSDAILLSDILRVNTEITKAPLSASMIKRYDEHHQDLTLLKALVRQQLPEKYKEIFFDQSKNGYAGYIDGGASQ
EEFYKFIKPILEKMDGTEELLVKLNREDLLRKQRTFDNGSIPHQIHLGELHAILRRQEDFYPFLKDNREKIEKILTFRIPY
YVGPLARGNSRFAWMTRKSEETITPWNFEEVVDKGASAQSFIERMTNFDKNLPNEKVLPKHSLLYEYFTVYNELTKVKYVT
EGMRKPAFLSGEQKKAIVDLLFKTNRKVTVKQLKEDYFKKIECFDSVEISGVEDRFNASLGTYHDLLKIIKDKDFLDNEEN
EDILEDIVLTLTLFEDREMIEERLKTYAHLFDDKVMKQLKRRRYTGWGRLSRKLINGIRDKQSGKTILDFLKSDGFANRNF
MQLIHDDSLTFKEDIQKAQVSGQGDSLHEHIANLAGSPAIKKGILQTVKVVDELVKVMGRHKPENIVIEMARENQTTQKGQ
KNSRERMKRIEEGIKELGSQILKEHPVENTQLQNEKLYLYYLQNGRDMYVDQELDINRLSDYDVDHIVPQSFLKDDSIDNK
VLTRSDKNRGKSDNVPSEEVVKKMKNYWRQLLNAKLITQRKFDNLTKAERGGLSELDKAGFIKRQLVETRQITKHVAQILD
SRMNTKYDENDKLIREVKVITLKSKLVSDFRKDFQFYKVREINNYHHAHDAYLNAVVGTALIKKYPKLESEFVYGDYKVYD
VRKMIAKSEQEIGKATAKYFFYSNIMNFFKTEITLANGEIRKRPLIETNGETGEIVWDKGRDFATVRKVLSMPQVNIVKKT
EVQTGGFSKESILPKRNSDKLIARKKDWDPKKYGGFDSPTVAYSVLVVAKVEKGKSKKLKSVKELLGITIMERSSFEKNPI
DFLEAKGYKEVKKDLIIKLPKYSLFELENGRKRMLASAGELQKGNELALPSKYVNFLYLASHYEKLKGSPEDNEQKQLFVE
QHKHYLDEIIEQISEFSKRVILADANLDKVLSAYNKHRDKPIREQAENIIHLFTLTNLGAPAAFKYFDTTIDRKRYTSTKE
VLDATLIHQSITGLYETRIDLSQLGGDSGGSGGSGGSTNLSDIIEKETGKQLVIQESILMLPEEVEEVIGNKPESDILVHT
AYDESTDENVMLLTSDAPEYKPWALVIQDSNGENKIKMLSGGSGGSGGSTNLSDIIEKETGKQLVIQESILMLPEEVEEVI
GNKPESDILVHTAYDESTDENVMLLTSDAPEYKPWALVIQDSNGENKIKMLPKKKRKVEGADKRTADGSEFESPKKKRKV*

Supplementary Sequence 26: CBE-T1.31

MSEVEYSHEYWMRHALTLAKRARDERHVPVGAVLVLNNRVIGEGWNRAKGLHDPTAHAEIMALRQGGLVMQNYRLWGATLY
TTFEPCVMCAGAMIHSRIGRVVFGVCNAKTHAAMSLMDVLHHPGMPHRVEITEGILADECEALLCRFFRMPRRVFNAQKKA
QSSTDSGGSSGGSSGSETPGTSESATPESSGGSSGGSDKKYSIGLAIGTNSVGWAVITDEYKVPSKKFKVLGNTDRHSIKK
NLIGALLFDSGETAEATRLKRTARRRYTRRKNRICYLQEIFSNEMAKVDDSFFHRLEESFLVEEDKKHERHPIFGNIVDEV
AYHEKYPTIYHLRKKLVDSTDKADLRLIYLALAHMIKFRGHFLIEGDLNPDNSDVDKLFIQLVQTYNQLFEENPINASGVD
AKAILSARLSKSRRLENLIAQLPGEKKNGLFGNLIALSLGLTPNFKSNFDLAEDAKLQLSKDTYDDDLDNLLAQIGDQYAD
LFLAAKNLSDAILLSDILRVNTEITKAPLSASMIKRYDEHHQDLTLLKALVRQQLPEKYKEIFFDQSKNGYAGYIDGGASQ
EEFYKFIKPILEKMDGTEELLVKLNREDLLRKQRTFDNGSIPHQIHLGELHAILRRQEDFYPFLKDNREKIEKILTFRIPY
YVGPLARGNSRFAWMTRKSEETITPWNFEEVVDKGASAQSFIERMTNFDKNLPNEKVLPKHSLLYEYFTVYNELTKVKYVT
EGMRKPAFLSGEQKKAIVDLLFKTNRKVTVKQLKEDYFKKIECFDSVEISGVEDRFNASLGTYHDLLKIIKDKDFLDNEEN
EDILEDIVLTLTLFEDREMIEERLKTYAHLFDDKVMKQLKRRRYTGWGRLSRKLINGIRDKQSGKTILDFLKSDGFANRNF
MQLIHDDSLTFKEDIQKAQVSGQGDSLHEHIANLAGSPAIKKGILQTVKVVDELVKVMGRHKPENIVIEMARENQTTQKGQ
KNSRERMKRIEEGIKELGSQILKEHPVENTQLQNEKLYLYYLQNGRDMYVDQELDINRLSDYDVDHIVPQSFLKDDSIDNK
VLTRSDKNRGKSDNVPSEEVVKKMKNYWRQLLNAKLITQRKFDNLTKAERGGLSELDKAGFIKRQLVETRQITKHVAQILD
SRMNTKYDENDKLIREVKVITLKSKLVSDFRKDFQFYKVREINNYHHAHDAYLNAVVGTALIKKYPKLESEFVYGDYKVYD
VRKMIAKSEQEIGKATAKYFFYSNIMNFFKTEITLANGEIRKRPLIETNGETGEIVWDKGRDFATVRKVLSMPQVNIVKKT
EVQTGGFSKESILPKRNSDKLIARKKDWDPKKYGGFDSPTVAYSVLVVAKVEKGKSKKLKSVKELLGITIMERSSFEKNPI
DFLEAKGYKEVKKDLIIKLPKYSLFELENGRKRMLASAGELQKGNELALPSKYVNFLYLASHYEKLKGSPEDNEQKQLFVE
QHKHYLDEIIEQISEFSKRVILADANLDKVLSAYNKHRDKPIREQAENIIHLFTLTNLGAPAAFKYFDTTIDRKRYTSTKE
VLDATLIHQSITGLYETRIDLSQLGGDSGGSGGSGGSTNLSDIIEKETGKQLVIQESILMLPEEVEEVIGNKPESDILVHT
AYDESTDENVMLLTSDAPEYKPWALVIQDSNGENKIKMLSGGSGGSGGSTNLSDIIEKETGKQLVIQESILMLPEEVEEVI
GNKPESDILVHTAYDESTDENVMLLTSDAPEYKPWALVIQDSNGENKIKMLPKKKRKVEGADKRTADGSEFESPKKKRKV*

Supplementary Sequence 27: CBE-T1.32

MSEVEYSHEYWMRHALTLAKRARDERHVPVGAVLVLNNRVIGEGWNRAKGLHDPTAHAEIMALRQGGLVMQNYRLWGATLY
TTFEPCVMCAGAMIHSRIGRVVFGVCNAKTHAAMSLMDVLHHPGMNHRVEITEGILADECEELLCRFFRMPRRVFNAQKKA
QSSTDSGGSSGGSSGSETPGTSESATPESSGGSSGGSDKKYSIGLAIGTNSVGWAVITDEYKVPSKKFKVLGNTDRHSIKK
NLIGALLFDSGETAEATRLKRTARRRYTRRKNRICYLQEIFSNEMAKVDDSFFHRLEESFLVEEDKKHERHPIFGNIVDEV
AYHEKYPTIYHLRKKLVDSTDKADLRLIYLALAHMIKFRGHFLIEGDLNPDNSDVDKLFIQLVQTYNQLFEENPINASGVD
AKAILSARLSKSRRLENLIAQLPGEKKNGLFGNLIALSLGLTPNFKSNFDLAEDAKLQLSKDTYDDDLDNLLAQIGDQYAD
LFLAAKNLSDAILLSDILRVNTEITKAPLSASMIKRYDEHHQDLTLLKALVRQQLPEKYKEIFFDQSKNGYAGYIDGGASQ
EEFYKFIKPILEKMDGTEELLVKLNREDLLRKQRTFDNGSIPHQIHLGELHAILRRQEDFYPFLKDNREKIEKILTFRIPY
YVGPLARGNSRFAWMTRKSEETITPWNFEEVVDKGASAQSFIERMTNFDKNLPNEKVLPKHSLLYEYFTVYNELTKVKYVT
EGMRKPAFLSGEQKKAIVDLLFKTNRKVTVKQLKEDYFKKIECFDSVEISGVEDRFNASLGTYHDLLKIIKDKDFLDNEEN
EDILEDIVLTLTLFEDREMIEERLKTYAHLFDDKVMKQLKRRRYTGWGRLSRKLINGIRDKQSGKTILDFLKSDGFANRNF
MQLIHDDSLTFKEDIQKAQVSGQGDSLHEHIANLAGSPAIKKGILQTVKVVDELVKVMGRHKPENIVIEMARENQTTQKGQ
KNSRERMKRIEEGIKELGSQILKEHPVENTQLQNEKLYLYYLQNGRDMYVDQELDINRLSDYDVDHIVPQSFLKDDSIDNK
VLTRSDKNRGKSDNVPSEEVVKKMKNYWRQLLNAKLITQRKFDNLTKAERGGLSELDKAGFIKRQLVETRQITKHVAQILD
SRMNTKYDENDKLIREVKVITLKSKLVSDFRKDFQFYKVREINNYHHAHDAYLNAVVGTALIKKYPKLESEFVYGDYKVYD
VRKMIAKSEQEIGKATAKYFFYSNIMNFFKTEITLANGEIRKRPLIETNGETGEIVWDKGRDFATVRKVLSMPQVNIVKKT
EVQTGGFSKESILPKRNSDKLIARKKDWDPKKYGGFDSPTVAYSVLVVAKVEKGKSKKLKSVKELLGITIMERSSFEKNPI
DFLEAKGYKEVKKDLIIKLPKYSLFELENGRKRMLASAGELQKGNELALPSKYVNFLYLASHYEKLKGSPEDNEQKQLFVE
QHKHYLDEIIEQISEFSKRVILADANLDKVLSAYNKHRDKPIREQAENIIHLFTLTNLGAPAAFKYFDTTIDRKRYTSTKE
VLDATLIHQSITGLYETRIDLSQLGGDSGGSGGSGGSTNLSDIIEKETGKQLVIQESILMLPEEVEEVIGNKPESDILVHT
AYDESTDENVMLLTSDAPEYKPWALVIQDSNGENKIKMLSGGSGGSGGSTNLSDIIEKETGKQLVIQESILMLPEEVEEVI
GNKPESDILVHTAYDESTDENVMLLTSDAPEYKPWALVIQDSNGENKIKMLPKKKRKVEGADKRTADGSEFESPKKKRKV*

Supplementary Sequence 28: CBE-T1.45

MSEVEYSHEYWMRHALTLAKRARDERHVPVGAVLVLNNRVIGEGWNRAKGLHDPTAHAEIMALRQGGLVMQNYRLWGATLY
TTFEPCVMCAGAMIHSRIGRVVFGVCNAKTHACMSLMNVLHHPGMNHRVEITEGILADECEALLCRFFRMPRRVFNAQKKA
QSSTDSGGSSGGSSGSETPGTSESATPESSGGSSGGSDKKYSIGLAIGTNSVGWAVITDEYKVPSKKFKVLGNTDRHSIKK
NLIGALLFDSGETAEATRLKRTARRRYTRRKNRICYLQEIFSNEMAKVDDSFFHRLEESFLVEEDKKHERHPIFGNIVDEV
AYHEKYPTIYHLRKKLVDSTDKADLRLIYLALAHMIKFRGHFLIEGDLNPDNSDVDKLFIQLVQTYNQLFEENPINASGVD
AKAILSARLSKSRRLENLIAQLPGEKKNGLFGNLIALSLGLTPNFKSNFDLAEDAKLQLSKDTYDDDLDNLLAQIGDQYAD
LFLAAKNLSDAILLSDILRVNTEITKAPLSASMIKRYDEHHQDLTLLKALVRQQLPEKYKEIFFDQSKNGYAGYIDGGASQ
EEFYKFIKPILEKMDGTEELLVKLNREDLLRKQRTFDNGSIPHQIHLGELHAILRRQEDFYPFLKDNREKIEKILTFRIPY
YVGPLARGNSRFAWMTRKSEETITPWNFEEVVDKGASAQSFIERMTNFDKNLPNEKVLPKHSLLYEYFTVYNELTKVKYVT
EGMRKPAFLSGEQKKAIVDLLFKTNRKVTVKQLKEDYFKKIECFDSVEISGVEDRFNASLGTYHDLLKIIKDKDFLDNEEN
EDILEDIVLTLTLFEDREMIEERLKTYAHLFDDKVMKQLKRRRYTGWGRLSRKLINGIRDKQSGKTILDFLKSDGFANRNF
MQLIHDDSLTFKEDIQKAQVSGQGDSLHEHIANLAGSPAIKKGILQTVKVVDELVKVMGRHKPENIVIEMARENQTTQKGQ
KNSRERMKRIEEGIKELGSQILKEHPVENTQLQNEKLYLYYLQNGRDMYVDQELDINRLSDYDVDHIVPQSFLKDDSIDNK
VLTRSDKNRGKSDNVPSEEVVKKMKNYWRQLLNAKLITQRKFDNLTKAERGGLSELDKAGFIKRQLVETRQITKHVAQILD
SRMNTKYDENDKLIREVKVITLKSKLVSDFRKDFQFYKVREINNYHHAHDAYLNAVVGTALIKKYPKLESEFVYGDYKVYD
VRKMIAKSEQEIGKATAKYFFYSNIMNFFKTEITLANGEIRKRPLIETNGETGEIVWDKGRDFATVRKVLSMPQVNIVKKT
EVQTGGFSKESILPKRNSDKLIARKKDWDPKKYGGFDSPTVAYSVLVVAKVEKGKSKKLKSVKELLGITIMERSSFEKNPI
DFLEAKGYKEVKKDLIIKLPKYSLFELENGRKRMLASAGELQKGNELALPSKYVNFLYLASHYEKLKGSPEDNEQKQLFVE
QHKHYLDEIIEQISEFSKRVILADANLDKVLSAYNKHRDKPIREQAENIIHLFTLTNLGAPAAFKYFDTTIDRKRYTSTKE
VLDATLIHQSITGLYETRIDLSQLGGDSGGSGGSGGSTNLSDIIEKETGKQLVIQESILMLPEEVEEVIGNKPESDILVHT
AYDESTDENVMLLTSDAPEYKPWALVIQDSNGENKIKMLSGGSGGSGGSTNLSDIIEKETGKQLVIQESILMLPEEVEEVI
GNKPESDILVHTAYDESTDENVMLLTSDAPEYKPWALVIQDSNGENKIKMLPKKKRKVEGADKRTADGSEFESPKKKRKV*

Supplementary Sequence 29: CBE-T1.46

MSEVEYSHEYWMRHALTLAKRARDERHVPVGAVLVLNNRVIGEGWNRAKGLHDPTAHAEIMALRQGGLVMQNYRLWGATLY
TTFEPCVMCAGAMIHSRIGRVVFGVCNAKTHACMSLMDVLGHPGMNHRVEITEGILADECEALLCRFFRMPRRVFNAQKKA
QSSTDSGGSSGGSSGSETPGTSESATPESSGGSSGGSDKKYSIGLAIGTNSVGWAVITDEYKVPSKKFKVLGNTDRHSIKK
NLIGALLFDSGETAEATRLKRTARRRYTRRKNRICYLQEIFSNEMAKVDDSFFHRLEESFLVEEDKKHERHPIFGNIVDEV
AYHEKYPTIYHLRKKLVDSTDKADLRLIYLALAHMIKFRGHFLIEGDLNPDNSDVDKLFIQLVQTYNQLFEENPINASGVD
AKAILSARLSKSRRLENLIAQLPGEKKNGLFGNLIALSLGLTPNFKSNFDLAEDAKLQLSKDTYDDDLDNLLAQIGDQYAD
LFLAAKNLSDAILLSDILRVNTEITKAPLSASMIKRYDEHHQDLTLLKALVRQQLPEKYKEIFFDQSKNGYAGYIDGGASQ
EEFYKFIKPILEKMDGTEELLVKLNREDLLRKQRTFDNGSIPHQIHLGELHAILRRQEDFYPFLKDNREKIEKILTFRIPY
YVGPLARGNSRFAWMTRKSEETITPWNFEEVVDKGASAQSFIERMTNFDKNLPNEKVLPKHSLLYEYFTVYNELTKVKYVT
EGMRKPAFLSGEQKKAIVDLLFKTNRKVTVKQLKEDYFKKIECFDSVEISGVEDRFNASLGTYHDLLKIIKDKDFLDNEEN
EDILEDIVLTLTLFEDREMIEERLKTYAHLFDDKVMKQLKRRRYTGWGRLSRKLINGIRDKQSGKTILDFLKSDGFANRNF
MQLIHDDSLTFKEDIQKAQVSGQGDSLHEHIANLAGSPAIKKGILQTVKVVDELVKVMGRHKPENIVIEMARENQTTQKGQ
KNSRERMKRIEEGIKELGSQILKEHPVENTQLQNEKLYLYYLQNGRDMYVDQELDINRLSDYDVDHIVPQSFLKDDSIDNK
VLTRSDKNRGKSDNVPSEEVVKKMKNYWRQLLNAKLITQRKFDNLTKAERGGLSELDKAGFIKRQLVETRQITKHVAQILD
SRMNTKYDENDKLIREVKVITLKSKLVSDFRKDFQFYKVREINNYHHAHDAYLNAVVGTALIKKYPKLESEFVYGDYKVYD
VRKMIAKSEQEIGKATAKYFFYSNIMNFFKTEITLANGEIRKRPLIETNGETGEIVWDKGRDFATVRKVLSMPQVNIVKKT
EVQTGGFSKESILPKRNSDKLIARKKDWDPKKYGGFDSPTVAYSVLVVAKVEKGKSKKLKSVKELLGITIMERSSFEKNPI
DFLEAKGYKEVKKDLIIKLPKYSLFELENGRKRMLASAGELQKGNELALPSKYVNFLYLASHYEKLKGSPEDNEQKQLFVE
QHKHYLDEIIEQISEFSKRVILADANLDKVLSAYNKHRDKPIREQAENIIHLFTLTNLGAPAAFKYFDTTIDRKRYTSTKE
VLDATLIHQSITGLYETRIDLSQLGGDSGGSGGSGGSTNLSDIIEKETGKQLVIQESILMLPEEVEEVIGNKPESDILVHT
AYDESTDENVMLLTSDAPEYKPWALVIQDSNGENKIKMLSGGSGGSGGSTNLSDIIEKETGKQLVIQESILMLPEEVEEVI
GNKPESDILVHTAYDESTDENVMLLTSDAPEYKPWALVIQDSNGENKIKMLPKKKRKVEGADKRTADGSEFESPKKKRKV*

Supplementary Sequence 30: CBE-T1.52

MSEVEYSHEYWMRHALTLAKRARDERHVPVGAVLVLNNRVIGEGWNRAKGLHDPTAHAEIMALRQGGLVMQNYRLWGATLY
TTFEPCVMCAGAMIHSRIGRVVFGVCNAKTHACMSLMDVLGHPGMPHRVEITEGILADECEALLCRFFRMPRRVFNAQKKA
QSSTDSGGSSGGSSGSETPGTSESATPESSGGSSGGSDKKYSIGLAIGTNSVGWAVITDEYKVPSKKFKVLGNTDRHSIKK
NLIGALLFDSGETAEATRLKRTARRRYTRRKNRICYLQEIFSNEMAKVDDSFFHRLEESFLVEEDKKHERHPIFGNIVDEV
AYHEKYPTIYHLRKKLVDSTDKADLRLIYLALAHMIKFRGHFLIEGDLNPDNSDVDKLFIQLVQTYNQLFEENPINASGVD
AKAILSARLSKSRRLENLIAQLPGEKKNGLFGNLIALSLGLTPNFKSNFDLAEDAKLQLSKDTYDDDLDNLLAQIGDQYAD
LFLAAKNLSDAILLSDILRVNTEITKAPLSASMIKRYDEHHQDLTLLKALVRQQLPEKYKEIFFDQSKNGYAGYIDGGASQ
EEFYKFIKPILEKMDGTEELLVKLNREDLLRKQRTFDNGSIPHQIHLGELHAILRRQEDFYPFLKDNREKIEKILTFRIPY
YVGPLARGNSRFAWMTRKSEETITPWNFEEVVDKGASAQSFIERMTNFDKNLPNEKVLPKHSLLYEYFTVYNELTKVKYVT
EGMRKPAFLSGEQKKAIVDLLFKTNRKVTVKQLKEDYFKKIECFDSVEISGVEDRFNASLGTYHDLLKIIKDKDFLDNEEN
EDILEDIVLTLTLFEDREMIEERLKTYAHLFDDKVMKQLKRRRYTGWGRLSRKLINGIRDKQSGKTILDFLKSDGFANRNF
MQLIHDDSLTFKEDIQKAQVSGQGDSLHEHIANLAGSPAIKKGILQTVKVVDELVKVMGRHKPENIVIEMARENQTTQKGQ
KNSRERMKRIEEGIKELGSQILKEHPVENTQLQNEKLYLYYLQNGRDMYVDQELDINRLSDYDVDHIVPQSFLKDDSIDNK
VLTRSDKNRGKSDNVPSEEVVKKMKNYWRQLLNAKLITQRKFDNLTKAERGGLSELDKAGFIKRQLVETRQITKHVAQILD
SRMNTKYDENDKLIREVKVITLKSKLVSDFRKDFQFYKVREINNYHHAHDAYLNAVVGTALIKKYPKLESEFVYGDYKVYD
VRKMIAKSEQEIGKATAKYFFYSNIMNFFKTEITLANGEIRKRPLIETNGETGEIVWDKGRDFATVRKVLSMPQVNIVKKT
EVQTGGFSKESILPKRNSDKLIARKKDWDPKKYGGFDSPTVAYSVLVVAKVEKGKSKKLKSVKELLGITIMERSSFEKNPI
DFLEAKGYKEVKKDLIIKLPKYSLFELENGRKRMLASAGELQKGNELALPSKYVNFLYLASHYEKLKGSPEDNEQKQLFVE
QHKHYLDEIIEQISEFSKRVILADANLDKVLSAYNKHRDKPIREQAENIIHLFTLTNLGAPAAFKYFDTTIDRKRYTSTKE
VLDATLIHQSITGLYETRIDLSQLGGDSGGSGGSGGSTNLSDIIEKETGKQLVIQESILMLPEEVEEVIGNKPESDILVHT
AYDESTDENVMLLTSDAPEYKPWALVIQDSNGENKIKMLSGGSGGSGGSTNLSDIIEKETGKQLVIQESILMLPEEVEEVI
GNKPESDILVHTAYDESTDENVMLLTSDAPEYKPWALVIQDSNGENKIKMLPKKKRKVEGADKRTADGSEFESPKKKRKV*

Supplementary Sequence 31: CBE-T1.53

MSEVEYSHEYWMRHALTLAKRARDERHVPVGAVLVLNNRVIGEGWNRAKGLHDPTAHAEIMALRQGGLVMQNYRLWDATLY
TTFEPCVMCAGAMIHSRIGRVVFGVCNAKTHACMSLMNVLHHPGMPHRVEITEGILADECEELLCRFFRMPRRVFNAQKKA
QSSTDSGGSSGGSSGSETPGTSESATPESSGGSSGGSDKKYSIGLAIGTNSVGWAVITDEYKVPSKKFKVLGNTDRHSIKK
NLIGALLFDSGETAEATRLKRTARRRYTRRKNRICYLQEIFSNEMAKVDDSFFHRLEESFLVEEDKKHERHPIFGNIVDEV
AYHEKYPTIYHLRKKLVDSTDKADLRLIYLALAHMIKFRGHFLIEGDLNPDNSDVDKLFIQLVQTYNQLFEENPINASGVD
AKAILSARLSKSRRLENLIAQLPGEKKNGLFGNLIALSLGLTPNFKSNFDLAEDAKLQLSKDTYDDDLDNLLAQIGDQYAD
LFLAAKNLSDAILLSDILRVNTEITKAPLSASMIKRYDEHHQDLTLLKALVRQQLPEKYKEIFFDQSKNGYAGYIDGGASQ
EEFYKFIKPILEKMDGTEELLVKLNREDLLRKQRTFDNGSIPHQIHLGELHAILRRQEDFYPFLKDNREKIEKILTFRIPY
YVGPLARGNSRFAWMTRKSEETITPWNFEEVVDKGASAQSFIERMTNFDKNLPNEKVLPKHSLLYEYFTVYNELTKVKYVT
EGMRKPAFLSGEQKKAIVDLLFKTNRKVTVKQLKEDYFKKIECFDSVEISGVEDRFNASLGTYHDLLKIIKDKDFLDNEEN
EDILEDIVLTLTLFEDREMIEERLKTYAHLFDDKVMKQLKRRRYTGWGRLSRKLINGIRDKQSGKTILDFLKSDGFANRNF
MQLIHDDSLTFKEDIQKAQVSGQGDSLHEHIANLAGSPAIKKGILQTVKVVDELVKVMGRHKPENIVIEMARENQTTQKGQ
KNSRERMKRIEEGIKELGSQILKEHPVENTQLQNEKLYLYYLQNGRDMYVDQELDINRLSDYDVDHIVPQSFLKDDSIDNK
VLTRSDKNRGKSDNVPSEEVVKKMKNYWRQLLNAKLITQRKFDNLTKAERGGLSELDKAGFIKRQLVETRQITKHVAQILD
SRMNTKYDENDKLIREVKVITLKSKLVSDFRKDFQFYKVREINNYHHAHDAYLNAVVGTALIKKYPKLESEFVYGDYKVYD
VRKMIAKSEQEIGKATAKYFFYSNIMNFFKTEITLANGEIRKRPLIETNGETGEIVWDKGRDFATVRKVLSMPQVNIVKKT
EVQTGGFSKESILPKRNSDKLIARKKDWDPKKYGGFDSPTVAYSVLVVAKVEKGKSKKLKSVKELLGITIMERSSFEKNPI
DFLEAKGYKEVKKDLIIKLPKYSLFELENGRKRMLASAGELQKGNELALPSKYVNFLYLASHYEKLKGSPEDNEQKQLFVE
QHKHYLDEIIEQISEFSKRVILADANLDKVLSAYNKHRDKPIREQAENIIHLFTLTNLGAPAAFKYFDTTIDRKRYTSTKE
VLDATLIHQSITGLYETRIDLSQLGGDSGGSGGSGGSTNLSDIIEKETGKQLVIQESILMLPEEVEEVIGNKPESDILVHT
AYDESTDENVMLLTSDAPEYKPWALVIQDSNGENKIKMLSGGSGGSGGSTNLSDIIEKETGKQLVIQESILMLPEEVEEVI
GNKPESDILVHTAYDESTDENVMLLTSDAPEYKPWALVIQDSNGENKIKMLPKKKRKVEGADKRTADGSEFESPKKKRKV*

Blue – Evolved $T_{AD}AC$ or $T_{AD}C$ deaminase
Green – linker
Black – S. pyogenes Cas9 nickase (D10A)
Orange – 2x UGI
Purple – BP-NLS nuclear localization tag

References:

1 Gaudelli, N. M. *et al.* Directed evolution of adenine base editors with increased activity and therapeutic application. *Nat Biotechnol* **38**, 892-900, doi:10.1038/s41587-020-0491-6 (2020).

2 Kim, J. *et al.* Structural and kinetic characterization of Escherichia coli TadA, the wobble-specific tRNA deaminase. *Biochemistry* **45**, 6407-6416, doi:10.1021/bi0522394 (2006).

3 Lapinaite, A. *et al.* DNA capture by a CRISPR-Cas9-guided adenine base editor. *Science* **369**, 566-571, doi:10.1126/science.abb1390 (2020).

4 Losey, H. C., Ruthenburg, A. J. & Verdine, G. L. Crystal structure of Staphylococcus aureus tRNA adenosine deaminase TadA in complex with RNA. *Nat Struct Mol Biol* **13**, 153-159, doi:10.1038/nsmb1047 (2006).

5 Jeong, Y. K. *et al.* Adenine base editor engineering reduces editing of bystander cytosines. *Nat Biotechnol* **39**, 1426-1433, doi:10.1038/s41587-021-00943-2 (2021).

6 Anzalone, A. V., Koblan, L. W. & Liu, D. R. Genome editing with CRISPR-Cas nucleases, base editors, transposases and prime editors. *Nat Biotechnol* **38**, 824-844, doi:10.1038/s41587-020-0561-9 (2020).

7 Yu, Y. *et al.* Cytosine base editors with minimized unguided DNA and RNA off-target events and high on-target activity. *Nat Commun* **11**, 2052, doi:10.1038/s41467-020-15887-5 (2020).
