# Supplementary material for: Improved cytosine base editors generated from TadA variants
Source: Nat Biotechnol. 2023 Jan 9;41(5):686–97. doi: 10.1038/s41587-022-01611-9 (PMC10188367; doi:10.1038/s41587-022-01611-9)
Supplement: Supplementary file 2 — Reporting Summary [file 41587_2022_1611_MOESM2_ESM.pdf]

nature research

Corresponding author(s): Nicole M. Gaudelli

Last updated by author(s): 10/07/2022

Reporting Summary

Nature Research wishes to improve the reproducibility of the work that we publish. This form provides structure for consistency and transparency in reporting. For further information on Nature Research policies, see Authors & Referees and the Editorial Policy Checklist.

Statistics

For all statistical analyses, confirm that the following items are present in the figure legend, table legend, main text, or Methods section.

n/a	Confirmed	
☐	☒	The exact sample size (*n*) for each experimental group/condition, given as a discrete number and unit of measurement
☐	☒	A statement on whether measurements were taken from distinct samples or whether the same sample was measured repeatedly
☐	☒	The statistical test(s) used AND whether they are one- or two-sided<br>*Only common tests should be described solely by name; describe more complex techniques in the Methods section.*
☒	☐	A description of all covariates tested
☒	☐	A description of any assumptions or corrections, such as tests of normality and adjustment for multiple comparisons
☐	☒	A full description of the statistical parameters including central tendency (e.g. means) or other basic estimates (e.g. regression coefficient) AND variation (e.g. standard deviation) or associated estimates of uncertainty (e.g. confidence intervals)
☐	☒	For null hypothesis testing, the test statistic (e.g. *F*, *t*, *r*) with confidence intervals, effect sizes, degrees of freedom and *P* value noted<br>*Give P values as exact values whenever suitable.*
☒	☐	For Bayesian analysis, information on the choice of priors and Markov chain Monte Carlo settings
☒	☐	For hierarchical and complex designs, identification of the appropriate level for tests and full reporting of outcomes
☒	☐	Estimates of effect sizes (e.g. Cohen's *d*, Pearson's *r*), indicating how they were calculated

Our web collection on statistics for biologists contains articles on many of the points above.

Software and code

Policy information about availability of computer code

Data collection	Illumina MiSeq Control Software (v3.1), Thermo Scientific Chromeleon (v7.2.9)
Data analysis	Illumina bcl2fastq (v2.20.0.422), trimmomatic (v0.39), bowtie2 (v2.35), samtools (v1.9), bam-readcounts (v0.8), R (v3.4.3), FlowJo (v10.6.1), bwa-mem2 (v2.2.1), Picard (v2.21.7), GATK (v4.1.4.1), LoFreq (v2.1.5), XDS (v2022Jan01), Aimless (v0.7.7), Phaser(v2.8), Phenix (v1.19.2-4158), Coot (v7766), PyMol (v2.5)

For manuscripts utilizing custom algorithms or software that are central to the research but not yet described in published literature, software must be made available to editors/reviewers. We strongly encourage code deposition in a community repository (e.g. GitHub). See the Nature Research guidelines for submitting code & software for further information.

Data

Policy information about availability of data

All manuscripts must include a data availability statement. This statement should provide the following information, where applicable:

- Accession codes, unique identifiers, or web links for publicly available datasets
- A list of figures that have associated raw data
- A description of any restrictions on data availability

Next-generation sequencing data underlying all experiments are deposited in the NCBI Sequence Read Archive (PRJNA869750). The atomic coordinates and structure factors have been deposited in the Protein Data Bank as entries: 8E2P, 8E2Q, 8E2R, and 8E2S.

Field-specific reporting

Please select the one below that is the best fit for your research. If you are not sure, read the appropriate sections before making your selection.

☒ Life sciences ☐ Behavioural & social sciences ☐ Ecological, evolutionary & environmental sciences

For a reference copy of the document with all sections, see nature.com/documents/nr-reporting-summary-flat.pdf

Life sciences study design

All studies must disclose on these points even when the disclosure is negative.

Sample size	No sample size calculations were performed in advance.
Data exclusions	No data were excluded from the study.
Replication	All measurements reported in the study were collected in at least three independent biological replicates, except for initial engineering screens
Randomization	Randomization was not considered, as all major experimental variables were fully under the control of the experimenters.
Blinding	Blinding was not considered, as all data analysis was performed following uniform automated processes.

Reporting for specific materials, systems and methods

We require information from authors about some types of materials, experimental systems and methods used in many studies. Here, indicate whether each material, system or method listed is relevant to your study. If you are not sure if a list item applies to your research, read the appropriate section before selecting a response.

Materials & experimental systems

n/a	Involved in the study
☐	☒ Antibodies
☐	☒ Eukaryotic cell lines
☒	☐ Palaeontology
☒	☐ Animals and other organisms
☒	☐ Human research participants
☒	☐ Clinical data

Methods

n/a	Involved in the study
☒	☐ ChIP-seq
☐	☒ Flow cytometry
☒	☐ MRI-based neuroimaging

Antibodies

Antibodies used	B2M: Supplied by Biolegend. Clone 2M2. Catalog #316306. Lot #B335217 (WGS) and #B339354 (T cells)<br>TCRa/b: Supplied by Biolegend. Clone IP26. Lot #B316067<br>PD-1: Supplied by Biolegend. Clone NAT105. Lot #B349676
Validation	All antibodies were validated by the suppliers by staining using isotype controls.

Eukaryotic cell lines

Policy information about cell lines

Cell line source(s)	Hek293T: American Type Cell Culture Collection (ATCC)
Authentication	Done by supplier
Mycoplasma contamination	Tested negative for mycoplasma contamination by the supplier, ATCC.
Commonly misidentified lines<br>(See ICLAC register)	The cell line used is not listed as commonly misidentified.

Flow Cytometry

Plots

Confirm that:

☒ The axis labels state the marker and fluorochrome used (e.g. CD4-FITC).

☒ The axis scales are clearly visible. Include numbers along axes only for bottom left plot of group (a 'group' is an analysis of identical markers).

☒ All plots are contour plots with outliers or pseudocolor plots.

☒ A numerical value for number of cells or percentage (with statistics) is provided.

Methodology

Sample preparation	To assess editing efficiency, one million cells were taken from culture five days post electroporation and stained with the primary anti-human antibodies listed in the section above. The cells were incubated with antibodies according to manufacturer's instructions for 15 min at room temperature, washed, then analyzed on the flow cytometer.
Instrument	Attune NxT Flow Cytometer
Software	FlowJo (v10.8.1)
Cell population abundance	Not applicable, as no sorted populations were analyzed separately
Gating strategy	Linear FSC-A versus linear SSC-A was used to select the lymphocyte fraction from bulk cell suspension. Within the lymphocyte gate, FSC-A versus FSC-H was used to gate on single cells. Then within the live cell gate, a histogram gate was utilized on a negative control population to delineate the boundary between positive and negative cells. The values in the histogram represent surface protein knockout in the context of gene editing experiments.

☒ Tick this box to confirm that a figure exemplifying the gating strategy is provided in the Supplementary Information.
